# Synthesis, Characterization and Biological Activity of Alkyl 3-(*N*-Butylacetamido)propanoates Against Ixodid Ticks

**DOI:** 10.3390/molecules31193570

**Published:** 2026-10-08

**Authors:** Sergey V. Andreev, Ekaterina I. Anosova, Alisa P. Terskova, Varvara A. Morenova, Evita A. Kostenko, Sergey A. Zverev, Andrey S. Kuzovlev

**Affiliations:** Disinfectology Institute of F.F. Erisman FSCH of Rospotrebnadzor, 18a Nauchny Proezd, Moscow 117246, Russia

**Keywords:** alkyl 3-(*N*-butylacetamido)propanoates, repellent, IR3535^®^, biological activity, taiga ticks, ixodid ticks

## Abstract

Chemicals are widely used to protect humans from biological threats. Ticks are among the most dangerous biological species, as they transmit dangerous diseases such as Q-fever, Crimean–Congo hemorrhagic fever, tick-borne encephalitis, and others. It is not feasible nor viable to eradicate all tick populations in areas where people spend their leisure time or work. Personal protective equipment in general, and repellents in particular, have therefore become an important means of protection against this biological threat. Previous studies have shown that despite the repellent activity of substances currently used for this purpose, their effective concentrations against the taiga tick are not fit for practical application. In this study, we synthesized a homologous series of alkyl 3-(*N*-butylacetamido)propanoates with alkyl groups ranging from C1 to C12 and characterized these compounds using nuclear magnetic resonance (NMR) spectroscopy, infrared (IR) spectroscopy, and mass spectrometry. We also investigated the biological activity of all the synthesized compounds against the *Ixodes persulcatus* ticks. We demonstrated that the methyl, propyl and butyl esters of 3-(*N*-butylacetamido)propanoate exhibit the highest biological activity with an activity level approximately six times higher than that of the commercially available repellent IR3535^®^.

## 1. Introduction

Controlling zoonotic diseases plays a crucial role in maintaining epidemiological well-being. Many dangerous pathogens exist in the environment and can infect humans through direct contact or via arthropods and rodents [1,2,3]. One of the most epidemiologically dangerous groups of arthropods is the Ixodidae family of ticks. The taiga tick (*Ixodes persulcatus*) poses the greatest threat due to its wide range and the pathogens it transmits: Crimean–Congo hemorrhagic fever, Lyme disease, relapsing fever (borreliosis), rickettsial diseases (e.g., spotted fever and Q fever), tick-borne encephalitis and tularemia [4,5,6,7].

Personal protective equipment remains essential for preventing tick bites, which pose the greatest risk to field workers and campers. For workers, protective clothing is usually the primary mean of protection [8]. Depending on the types of ticks found in the working area, this clothing may be treated with cypermethrin or permethrin. The main limitation of this clothing is its high density of fabric, which causes significant discomfort under hot and humid conditions. The general population typically relies on repellents such as *N*,*N*-diethyl-3-methylbenzamide (DEET), ethyl 3-(*N*-butylacetamido)propanoate (IR3535^®^), or icaridin to prevent tick bites [9,10], although these compounds are more commonly associated with mosquito protection. Previously, we have demonstrated that concentrations of DEET and IR3535^®^ of approximately 66 g/m^2^ are required, so that is possible to achieve high repellent activity against *I. persulcatus*, whereas icaridin showed no significant result. Therefore, traditional repellents are difficult to use and cannot provide a high level of protection [11].

One way to increase the activity of chemical compounds is to modify their structure. Previous studies have shown that biological activity depends nonlinearly on the length of the alkyl radical. Studies on benzalkonium chloride, for example, show that homologues with C8 and C10 alkyl chains exhibit weak bactericidal activity. The C12 and C14 homologues were found to be the most active, whereas increasing the carbon chain length to 16 or 18 carbon atoms resulted in a slight decrease in antimicrobial activity [12].

A study of the insecticidal activity of alkyl furans against *S. exigua* showed that the C14, C15 and C16 homologues exhibited the highest activity, whereas molecules with C13 and C17 alkyl groups exhibited much lower activity [13]. The authors of the study [14] investigated the synthesis and insecticidal activity of azidopyridyl-containing dichloropropene ether derivatives. They showed that insecticide pyridalyl derivatives with an average alkyl chain length exhibit the highest activity against the cabbage moth (*P. xylostella*).

The authors of article [15] synthesized thirty-eight novel derivatives of Chasmanthinine in which the lipophilic moiety had been varied and investigated their activity against *Spodoptera exigua*. The alkyl substituents, ranging from ethyl to pentadecyl, exhibited different levels of activity. The compound with palmitoyl fragment showed the strongest cytotoxicity to Sf9 cells among all derivatives. The mechanism of Sf9 cytotoxicity was explained by morphological observation, fluorescence staining and DNA ladder, which indicated that the mechanism of cytotoxicity may be related to apoptosis.

The authors of another study [16] found no significant effect of alkyl chain length on the herbicidal activity of ionic liquids containing the 2-(2,4-dichlorophenoxy)propanoate (dichlorprop) anion and the 1-alkyl-1-methylpiperidinium cation.

Thus, varying the length of the alkyl chain can increase the biological activity of the compounds. In this study, we synthesized a series of homologous alkyl 3-(*N*-butylacetamido)propanoate esters with alkyl groups ranging from C1 to C12. We then investigated the structure, physicochemical properties, and repellent activity of these esters against the taiga tick *I. persulcatus*.

## 2. Results and Discussion

### 2.1. Structural Characterization of Synthesized Alkyl 3-(N-Butylacetamido)propanoates

All synthesized alkyl 3-(*N*-butylacetamido)propanoate derivatives were characterized using ^1^H and ^13^C NMR spectroscopy. Analysis of the spectra revealed that the compounds exist as an equilibrium mixture of two rotamers in CDCl_3_ solution due to delocalized electron density between the nitrogen atom and the carbonyl group. This equilibrium is evident in the NMR spectra as the duplication and overlap of signal sets that are characteristic of the two stable molecular conformations (Table 1 and Table 2).

The overlap of ^1^H-NMR signals from the two conformational isomers produces complex multiplet patterns. A defining feature of rotamer equilibrium is the presence of two singlet signals emanating from the methyl group of the acetyl fragment. This type of spectrum confirms that the energy barrier for rotation around the amide bond is sufficiently high to permit the observation of distinct signals for each rotamer, consistent with data from the literature for similar molecules [17,18]. The rotamer ratio for all the investigated compounds, as determined by the integral intensity of signal from the protons of the methyl group in the acetyl fragment, is approximately 30% for minor rotamer (MinR) and 70% for major rotamer (MajR), with only slight dependence on the length of the alkyl radical (Table 3).

The ^13^C-NMR spectra confirm rotameric equilibrium by splitting the signals from key carbon atoms, particularly those in the amide fragment: two alkyl carbons near the nitrogen atom and the carbonyl carbon.

The observed chemical shifts are consistent with the proposed structures of the synthesized compounds. The spectral characteristics, including the splitting of signals in the ^13^C NMR spectra and the shape of the signal for the methyl group of the acetyl fragment in the ^1^H NMR spectra, provide evidence not only for the structure of the molecules, but also for the presence of rotameric equilibrium in solution.

HPLC-MS/MS analysis revealed the fragmentation pattern of alkyl 3-(*N*-butylacetamido)propanoates. The mass spectrum of each substance contains parent ions respectively (*m*/*z*): 202, 216, 230, 244, 258, 272, 286, 300, 314, 328, 342, and 356. All compounds have fragment (daughter) ions with *m*/*z* values of 170 (C_9_H_17_NO_2_^+^), 128 (C_7_H_14_NO^+^), and 86 (C_5_H_12_N^+^), which indicates a common fragmentation mechanism (Figure 1). The spectra also contain marker ions (*m*/*z*) emerging from the splitting of an acetyl fragment: 160, 174, 188, 202, 216, 230, 244, 258, 272, 286, 300, and 314.

The IR spectra of the 3-(*N*-butylacetamido)propanoate alkyl esters obtained reveal three vibrational groups corresponding to the three main structural moieties: ester, amide, and alkyl groups (Figure 2). The valence vibrations of the ester C=O group appear as an intense band at ~1730 cm^−1^ for all derivatives. All compounds contain a tertiary amide moiety, which appears as an intense amide I band (valent vibrations of the amide C=O group) at ~1640 cm^−1^. The amide II–V bands are absent, as these are characteristic of primary and secondary amides. The three absorption bands at 2960, 2932, and 2874 cm^−1^ are identical for all compounds and correspond to the asymmetrical and symmetrical stretching vibrations of the -CH_3_ and -CH_2_ groups. These groups predominate in the structure of the 3-(*N*-butylacetamido)propanoate alkyl esters. The band at ~1370 cm^−1^, which corresponds to aliphatic C–H bending vibrations, exhibits minor shifts of less than 5 cm^−1^ when the spectra of the obtained derivatives are compared.

### 2.2. Study of the Repellent Action

There are numerous methods to evaluate repellency against ticks [19]. The majority of methods are based on determining how far ticks will go from a source of repellent. These approaches are correlated with the term ‘repellency’ [20]. In this study, we used a Petri dish choice trial, which allows the repellent effect to be determined and enables substances to be compared with each other.

The test involves placing a tick in a Petri dish containing untreated and treated textile samples. In this case, repellency is determined by the probability of the tick locating itself on an untreated textile sample. For each concentration 30 females of *I. persulcatus* were used. Ticks were collected in the natural foci of tick viral-borne encephalitis in the Irkutsk region, near Lake Baikal. All tests were carried out on the ticks’ collecting day or on the next day.

Initially, the dose–response curve for the commercial repellent IR3553© was evaluated. The point of maximum value just before the plateau on the repellency curve was chosen as the comparison concentration (Figure 3). This point corresponds to an ethyl 3-(*N*-butylacetamido)propanoate concentration of ~25 g/m^2^ and a repellency value of 0.86. This concentration was used to compare the repellency of all synthesized substances. Figure 4 shows the data obtained.

It was found that methyl, propyl, and butyl 3-(*N*-butylacetamido)propanoates exhibited higher biological activity compared to IR3535^®^ (Figure 4). The data presented show that the repellent activity of the compounds against the taiga ticks generally decreases as the length of the alkyl chain increases.

No significant correlation was observed between biological activity and whether the alkyl chain was even or odd. It was shown using Fisher’s exact test that the repellent activity of the methyl and butyl 3-(*N*-butylacetamido)propanoate is statistically significantly higher at a concentration of 25 g/m^2^ than that of IR3535^®^. Further studies were carried out on these compounds, as well as the propyl ester, in order to evaluate the dose–response curves. The resulting curves are shown in Figure 5.

The repellent activity of ethyl 3-(*N*-butylacetamido)propanoate does not follow a sigmoid curve. Rather it increases gradually along with the concentration. At a contrast, dose–response curves of methyl, propyl, and butyl esters are typically sigmoidal. This could be explained with the very low toxicity of commercial IR3535^®^.

A repellency value of 0.90 is achieved at an IR3535^®^ concentration of 36.8 g/m^2^ (Figure 3). The same level of biological activity was observed for the methyl ester at 6.87 g/m^2^, the propyl ester at 9.04 g/m^2^, and the butyl ester at 6.07 g/m^2^ (Figure 5). Thus, the derivatives obtained have a 4–6 times higher repellent activity against *I. persulcatus* than IR3535^®^. The butyl ester exhibits the highest activity. The data obtained for the methyl ester correlate with previous work, when authors have shown that this compound is more active against *Aedes albopictus*, *Culex pipiens pallens* and *Ae*. *togoi* [21]. Further toxicological studies are advisable to evaluate the potential use of these substances as active ingredients in repellents, particularly both butyl and methyl 3-(*N*-butylacetamido)propanoate.

## 3. Materials and Methods

### 3.1. Chemicals

Ethyl alcohol, HPLC grade (Macklin, Shanghai, China), methyl alcohol, HPLC grade (Merck KGaA, Darmstadt, Germany), propyl alcohol ≥99.0% (Acros Organics, Geel, Belgium), butyl alcohol ≥99.0% (Acros Organics, Geel, Belgium), amyl alcohol ≥99.0% (Merck KGaA, Darmstadt, Germany), hexyl alcohol ≥99.0% (Merck KGaA, Darmstadt, Germany), heptyl alcohol ≥98.0% (Acros Organics, Geel, Belgium), octyl alcohol ≥99.0% (Acros Organics, Geel, Belgium), nonyl alcohol ≥98.0% (Sigma-Aldrich, Saint Louis, MO, USA), decyl alcohol ≥99.0%, undecyl alcohol ≥99.0%, dodecyl alcohol ≥99.0%, IR3535^®^ 99.8% (all Merck KGaA, Darmstadt, Germany), potassium hydroxide ≥85.5% (CDH, New Delhi, India), methyl tert-butyl ether ≥99.5% (Pallav Chemicals, Mumbai, India), sodium bicarbonate 99.5% (neoFroxx, Einhausen, Germany), sodium sulfate anhydrous 99.0% (CDH, New Delhi, India), sodium chloride 98.5% (HiMedia Laboratories, Mumbai, India), toluene ≥99.0% (Acros Organics, Geel, Belgium), p-Toluenesulfonic acid 99.0% (Acros Organics, Geel, Belgium), potassium permanganate 99% (Sigma-Aldrich, Saint Louis, MO, USA), hydrochloric acid 37% (CDH, New Delhi, India), sulfuric acid 98% (Sigma-Aldrich, Saint Louis, MO, USA), acetonitrile, HPLC Gradient grade, 99.99% (CDH, New Delhi, India), and Chloroform-d, NMR grade, 99.8% (neoFroxx, Einhausen, Germany), were used; ultra-pure water prepared with a Millipore Direct-Q 3 UV-R was used throughout this work. All materials and chemicals were of analytical grade and used as purchased without further purification.

### 3.2. Methods

#### 3.2.1. Synthesis of 3-(*N*-Butylacetamido)propionic Acid

In a round-bottom flask, ethyl 3-(*N*-butylacetamido)propanoate (200.0 g, 0.933 mol) was dissolved in ethanol (900 mL), then potassium hydroxide (78.4 g, 1.40 mol) was added to the solution. The resulting mixture was stirred at reflux in an oil bath (90 °C) for 48 h. The reaction mixture was evaporated *in vacuo* and dissolved in water (300 mL). The resulting solution was acidified with 220 mL of concentrated hydrochloric acid whilst stirring. A light-brown precipitate was formed. The precipitate was filtrated and washed with cold water (50 mL) and dried under reduced pressure overnight, yielding 3-(*N*-butylacetamido)propionic acid as a brownish solid. The aqueous layer was extracted with TBME (3 × 15 mL). The combined organic layers were washed with sat. aq. NaCl, dried over Na_2_SO_4_, filtered and concentrated *in vacuo* to afford 3-(*N*-butylacetamido)propionic acid as a brownish solid. After combining the precipitates after the sedimentation and the extraction, we obtained 143 g of the target product (82% yield).

^1^H NMR (600 MHz, DMSO) δ 3.49–3.38 (m, 2H), 3.25–3.18 (m, 2H), 2.50–2.33 (m, 2H), 2.00 (MinR, s, 1H), 1.96 (MajR, s, 2H), 1.52–1.36 (m, 2H), 1.28–1.17 (m, 2H), 0.93–0.84 (m, 3H). ^13^C NMR (151 MHz, DMSO) δ 172.9, 172.6, 169.3, 169.2, 60.0, 59.8, 48.26, 44.0, 43.6, 41.5, 33.3, 32.5, 30.5, 29.3, 21.3, 21.2, 19.6, 19.4, 13.7, 13.6. IR (neat): 2960, 2928, 2869, 1714, 1588, 1495, 1426, 1380, 1366, 1358, 1298, 1291, 1192, 1020 cm^−1^.

#### 3.2.2. Synthesis of Alkyl 3-(*N*-Butylacetamido)propanoates

The synthesis was carried out using two different schemes depending on the alkyl substituent group (Figure 6). The progress of the reactions was monitored using thin-layer chromatography (TLC) on a fixed layer of silica gel (TLC Silica Gel 60 F254, 25-60 Å, F254 plates, Merck KGaA, Darmstadt, Germany). The spots were visualized using potassium permanganate (KMnO_4_) and cerium ammonium molybdate (CAM) as developing solutions.

##### General Procedure 1: Esterification of 3-(*N*-Butylacetamido)propionic Acid to Furnish Methyl, Propyl, and Butyl 3-(*N*-Butylacetylamido)propanoates

In a round-bottom flask, 3-(*N*-butylacetamido)propionic acid (1 equiv.) was dissolved in the corresponding alcohol, then H_2_SO_4_ (1 equiv.) was added to the solution. The resulting mixture was stirred at reflux in an oil bath for 24 h (TLC control in an acetone:heptane 1:1 mixture). The reaction mixture was poured into water; extracted three times with TBME; washed with water, a saturated NaHCO_3_ solution, and brine; dried over Na_2_SO_4_; and concentrated *in vacuo*.

*Methyl 3-(N-butylacetylamido)propanoates*: Following **GP1**, 3-(*N*-butylacetamido)propionic acid (1.0 equiv., 10.0 g, 53.4 mmol) was dissolved in methanol (40 mL), then H_2_SO_4_ (1.0 equiv., 5.2 g, 53.4 mmol) was added to the solution. After the work-up the title compound was obtained as a yellowish oil (8.0 g, 73% yield). ^1^H NMR (600 MHz, CDCl_3_) δ 3.71–3.63 (m, 3H), 3.61–3.55 (m, 2H), 3.33–3.24 (m, 2H), 2.63–2.56 (m, 2H), 2.15 (MinR, s, 1H), 2.10 (MajR, s, 2H), 1.57–1.46 (m, 2H), 1.35–1.27 (m, 2H), 0.96–0.88 (m, 3H). ^13^C NMR (151 MHz, CDCl_3_) δ 172.7, 171.5, 170.6, 170.3, 52.0, 51.7, 49.6, 45.3, 44.2, 42.4, 33.7, 32.7, 31.2, 29.8, 21.5, 21.5, 20.2, 20.0, 13.9, 13.8. IR (neat): 2958, 2936, 2875, 1735, 1637, 1436, 1376, 1200, 1174, 1061, 1028 cm^−1^. MS (ESI) calcd. for C_10_H_19_NO_3_ [M + H^+^]: 202.1; found: 202.1.

*Propyl 3-(N-butylacetylamido)propanoates*: Following **GP1**, 3-(*N*-butylacetamido)propionic acid (1.0 equiv., 5.0 g, 26.7 mmol) was dissolved in 1-propanol (40 mL), then H_2_SO_4_ (1.0 equiv., 2.6 g, 26.7 mmol) was added to the solution. After the work-up the title compound was obtained as a yellowish oil (5.2 g, 90% yield). ^1^H NMR (600 MHz, CDCl_3_) δ 4.10–3.98 (m, 2H), 3.63–3.56 (m, 2H), 3.35–3.27 (m, 2H), 2.64–2.55 (m, 2H), 2.20 (MinR, s, 1H), 2.14 (MajR, s, 2H), 1.68–1.61 (m, 2H), 1.36–1.27 (m, 2H), 0.97–0.89 (m, 6H). ^13^C NMR (151 MHz, CDCl_3_) δ 172.4, 171.2, 170.5, 170.2, 66.6, 66.3, 49.6, 49.3, 45.3, 44.3, 42.4, 33.9, 33.0, 31.2, 29.9, 22.0, 22.0, 21.6, 21.6, 20.3, 20.1, 13.9, 13.9, 10.4, 10.4. IR (neat): 2964, 2936, 2876, 1731, 1644, 1423, 1377, 1263, 1228, 1179, 1063 cm^−1^. MS (ESI) calcd. for C_12_H_23_NO_3_ [M + H^+^]: 230.2; found: 230.1.

*Butyl 3-(N-butylacetylamido)propanoates*: Following **GP1**, 3-(*N*-butylacetamido)propionic acid (1.0 equiv., 30.0 g, 160 mmol) was dissolved in 1-butanol (120 mL), then H_2_SO_4_ (1.0 equiv., 15.7 g, 160 mmol) was added to the solution. After the work-up the title compound was obtained as a yellowish oil (27.6 g, 71% yield). ^1^H NMR (300 MHz, CDCl_3_) δ 4.10–4.01 (m, 2H), 3.61–3.51 (m, 2H), 3.30–3.21 (m, 2H), 2.64–2.52 (m, 2H), 2.10 (MinR, s, 1H), 2.06 (MajR, s, 2H), 1.58–1.25 (m, 6H), 0.95–0.87 (m, 5H). ^13^C NMR (151 MHz, CDCl_3_) δ 172.4, 171.2, 170.5, 170.3, 71.1, 70.8, 49.6, 45.3, 44.3, 42.4, 33.9, 33.0, 31.2, 29.9, 27.7, 21.6, 21.5, 20.3, 20.1, 19.1, 13.9, 13.9. IR (neat): 2960, 2936, 2875, 1731, 1646, 1460, 1421, 1377, 1255, 1229, 1176, 1965, 1023 cm^−1^. MS (ESI) calcd. for C_13_H_25_NO_3_ [M + H^+^]: 244.1; found: 244.2.

##### General Procedure 2: Esterification of 3-(*N*-Butylacetamido)propionic Acid to Furnish Amyl, Hexyl, Heptyl, Octyl, Nonyl, Decyl, Undecyl, and Dodecyl 3-(*N*-Butylacetamido)propanoates

In a round-bottom flask equipped with a Dean–Stark head, 3-(*N*-butylacetamido)propionic acid (1 equiv.) was dissolved in toluene, then the corresponding alcohol (1 equiv.) and *p*-toluenesulfonic acid (0.022 equiv.) were added to the solution. The resulting mixture was stirred at reflux in an oil bath for 18 h (TLC control in acetone:heptane 1:1 mixture). The reaction mixture was poured into water; extracted three times with TBME; washed with water, a saturated NaHCO_3_ solution, and brine; dried over Na_2_SO_4_; and concentrated *in vacuo*.

*Amyl 3-(N-butylacetamido)propanoates*: Following **GP2**, 3-(*N*-butylacetamido)propionic acid (1.0 equiv., 5.0 g, 26.7 mmol) was dissolved in toluene (50 mL), then 1-pentanol (1.0 equiv., 2.9 mL, 26.7 mmol,) and *p-*toluenesulfonic acid (0.022 equiv., 0.10 g, 0.60 mmol) were added to the solution. After the work-up the title compound was obtained as a yellowish oil (5.7 g, 83% yield). ^1^H NMR (300 MHz, CDCl_3_) δ 4.12–4.02 (m, 2H), 3.66–3.55 (m, 2H), 3.37–3.26 (m, 2H), 2.67–2.53 (m, 2H), 2.20 (MinR, s, 1H), 2.16 (MajR, s, 2H), 1.68–1.45 (m, 4H), 1.38–1.26 (m, 6H), 0.98–0.85 (m, 6H). ^13^C NMR (151 MHz, CDCl_3_) δ 172.4, 171.2, 170.5, 170.2, 65.2, 64.8, 49.6, 45.3, 44.3, 42.4, 33.9, 33.0, 31.2, 29.9, 28.3, 28.1, 22.4, 21.63, 21.5, 20.3, 14.0, 13.8. IR (neat): 2959, 2935, 2873, 1731, 1646, 1457, 1419, 1376, 1257, 1227, 1174, 1055, 1020 cm^−1^. MS (ESI) calcd. for C_14_H_27_NO_3_ [M + H^+^]: 258.4; found: 258.4.

*Hexyl 3-(N-butylacetamido)propanoates*: Following **GP2**, 3-(*N*-butylacetamido)propionic acid (1.0 equiv., 5.0 g, 26.70 mmol) was dissolved in toluene (50 mL), then 1-hexanol (1.0 equiv., 3.3 mL, 26.7 mmol) and *p-*toluenesulfonic acid (0.022 equiv., 0.1 g, 0.60 mmol) were added to the solution. After the work-up the title compound was obtained as a yellowish oil (5.1 g, 70% yield). ^1^H NMR (600 MHz, CDCl_3_) δ 4.15–4.02 (m, 2H), 3.67–3.56 (m, 2H), 3.39–3.27 (m, 2H), 2.69–2.56 (m, 2H), 2.26 (MinR, s, 1H), 2.20 (MajR, s, 2H), 1.65–1.49 (m, 4H), 1.37–1.23 (m, 8H), 1.00–0.81 (m, 6H). ^13^C NMR (151 MHz, CDCl_3_) δ 172.3, 171.2, 170.5, 170.2, 65.2, 64.8, 49.6, 45.3, 44.3, 42.4, 33.9, 33.0, 31.5, 31.4, 31.2, 29.9, 28.6, 28.6, 25.6, 25.6, 22.6, 21.6, 21.5, 20.2, 20.1, 14.0, 13.9, 13.8. IR (neat): 2958, 2933, 2872, 2859, 1735, 1646, 1458, 1420, 1373, 1252, 1227, 1174, 1062, 1021 cm^−1^. MS (ESI) calcd. for C_15_H_29_NO_3_ [M + H^+^]: 272.4; found: 272.2.

*Heptyl 3-(N-butylacetamido)propanoates*: Following **GP2**, 3-(*N*-butylacetamido)propionic acid (1.0 equiv., 5.0 g, 26.7 mmol) was dissolved in toluene (50 mL), then 1-heptanol (1.0 equiv., 3.7 mL, 26.7 mmol) and *p-*toluenesulfonic acid (0.022 equiv., 0.1 g, 0.60 mmol) were added to the solution. After the work-up the title compound was obtained as a yellowish oil (6.1 g, 80% yield). ^1^H NMR (600 MHz, CDCl_3_) δ 4.12–4.02 (m, 2H), 3.59–3.54 (m, 2H), 3.32–3.23 (m, 2H), 2.62–2.52 (m, 2H), 2.11 (MinR, s, 1H), 2.06 (MajR, s, 2H), 1.65–1.57 (m, 2H), 1.56–1.46 (m, 2H), 1.36–1.21 (m, 10H), 0.97–0.83 (m, 6H). ^13^C NMR (151 MHz, CDCl_3_) δ 172.4, 171.2, 170.5, 170.3, 65.2, 64.9, 49.6, 45.4, 44.3, 42.4, 33.0, 31.8, 31.2, 29.9, 29.0, 28.7, 28.6, 25.9, 22.6, 21.6, 21.5, 20.3, 20.1, 14.1, 13.9, 13.9. IR (neat): 2958, 2931, 2873, 2859, 1734, 1648, 1457, 1419, 1376, 1256, 1227, 1174, 1060, 1021 cm^−1^. MS (ESI) calcd. for C_16_H_31_NO_3_ [M + H^+^]: 286.2; found 286.4.

*Octyl 3-(N-butylacetamido)propanoates*: Following **GP2**, 3-(*N*-butylacetamido)propionic acid (1.0 equiv., 10.0 g, 53.4 mmol) was dissolved in toluene (50 mL), then 1-octanol (1.0 equiv., 8.4 mL, 53.4 mmol) and *p-*toluenesulfonic acid (0.022 equiv., 0.2 g, 1.1 mmol) were added to the solution. After the work-up the title compound was obtained as a yellowish oil (13.0 g, 81% yield). ^1^H NMR (600 MHz, CDCl_3_) δ 4.10–4.02 (m, 2H), 3.60–3.53 (m, 2H), 3.32–3.23 (m, 2H), 2.62–2.53 (m, 2H), 2.11 (MinR, s, 1H), 2.06 (MajR, s, 2H), 1.64–1.57 (m, 2H), 1.56–1.46 (m, 2H), 1.35–1.24 (m, 12H), 0.97–0.84 (m, 6H). ^13^C NMR (151 MHz, CDCl_3_) δ 172.4, 171.2, 170.5, 170.3, 65.2, 64.9, 49.6, 45.3, 44.3, 42.4, 34.0, 33.0, 31.8, 31.2, 29.9, 29.3, 29.2, 28.7, 28.6, 26.0, 26.0, 22.7, 21.6, 21.5, 20.3, 20.1, 14.1, 13.9, 13.9. IR (neat): 2961, 2929, 2873, 2858, 1734, 1647, 1457, 1420, 1374, 1255, 1226, 1173, 1059, 1020 cm^−1^. MS (ESI) calcd. for C_17_H_33_NO_3_ [M + H^+^]: 300.2; found: 300.2.

*Nonyl 3-(N-butylacetamido)propanoates*: Following **GP2**, 3-(*N*-butylacetamido)propionic acid (1.0 equiv., 55.4 g, 296 mmol) was dissolved in toluene (277 mL), then 1-nonanol (1.0 equiv., 51.6 mL, 296 mmol) and *p-*toluenesulfonic acid (0.022 equiv., 1.1 g, 6.40 mmol) were added to the solution. After the work-up the title compound was obtained as a yellowish oil (39.1 g, 42% yield). ^1^H NMR (600 MHz, CDCl_3_) δ 4.10–4.02 (m, 2H), 3.61–3.54 (m, 2H), 3.33–3.23 (m, 2H), 2.63–2.54 (m, 2H), 2.11 (MinR, s, 1H), 2.07 (MajR, s, 2H), 1.65–1.58 (m, 2H), 1.56–1.48 (m, 2H), 1.32–1.22 (m, 14H), 0.96–0.86 (m, 6H). ^13^C NMR (151 MHz, CDCl_3_) δ 172.4, 171.2, 170.5, 170.3, 65.3, 64.9, 49.6, 45.4, 44.3, 42.4, 34.0, 33.0, 31.9, 31.3, 29.9, 29.6, 29.3, 28.6, 26.0, 22.7, 21.6, 21.6, 20.3, 20.1, 14.2, 13.9, 13.9. IR (neat): 2957, 2929, 2873, 2857, 1733, 1647, 1458, 1420, 1375, 1254, 1230, 1174, 1061, 1024 cm^−1^. MS (ESI) calcd. for C_18_H_35_NO_3_ [M + H^+^]: 314.2; found: 314.3.

*Decyl 3-(N-butylacetamido)propanoates*: Following **GP2**, 3-(*N*-butylacetamido)propionic acid (1.0 equiv., 35.0 g, 187 mmol) was dissolved in toluene (175 mL), then 1-decanol (1.0 equiv., 35.5 mL, 187 mmol,) and *p-*toluenesulfonic acid (0.022 equiv., 0.70 g, 4.10 mmol) were added to the solution. After the work-up the title compound was obtained as a yellowish oil (47.2 g, 77% yield). ^1^H NMR (600 MHz, CDCl_3_) δ 4.11–4.01 (m, 2H), 3.64–3.51 (m, 2H), 3.32–3.22 (m, 2H), 2.63–2.52 (m, 2H), 2.11 (MinR, s, 1H), 2.07 (MajR, s, 2H), 1.66–1.48 (m, 4H), 1.33–1.24 (m, 17H), 0.96–0.84 (m, 6H). ^13^C NMR (151 MHz, CDCl_3_) δ 172.4, 171.2, 170.5, 170.3, 65.3, 64.9, 49.6, 45.4, 44.3, 42.4, 33.0, 32.0, 31.3, 29.9, 29.8, 29.6, 29.4, 29.3, 28.7, 28.6, 26.0, 22.8, 21.6, 21.5, 20.3, 20.1, 14.2, 13.9. IR (neat): 2959, 2925, 2873, 2856, 1734, 1647, 1458, 1420, 1375, 1251, 1231, 1176, 1060, 1023 cm^−1^. MS (ESI) calcd. for C_19_H_37_NO_3_ [M + H^+^]: 328.3; found: 328.3.

*Undecyl 3-(N-butylacetamido)propanoates*: Following **GP2**, 3-(*N*-butylacetamido)propionic acid (1.0 equiv., 35.0 g, 187 mmol) was dissolved in toluene (175 mL), then 1-undecanol (1.0 equiv., 38.8 mL, 187 mmol,) and *p-*toluenesulfonic acid (0.022 equiv., 0.7 g, 4.10 mmol) were added to the solution. After the work-up the title compound was obtained as a yellowish oil (44.7 g, 70% yield). ^1^H NMR (600 MHz, CDCl_3_) δ 4.11–4.02 (m, 2H), 3.61–3.54 (m, 2H), 3.33–3.23 (m, 2H), 2.63–2.53 (m, 2H), 2.11 (MinR, s, 1H), 2.07 (MajR, s, 3H), 1.63–1.49 (m, 4H), 1.31–1.24 (m, 18H), 0.95–0.84 (m, 6H). ^13^C NMR (151 MHz, CDCl_3_) δ 172.4, 171.2, 170.5, 170.3, 65.3, 64.9, 49.6, 45.4, 44.3, 42.4, 34.0, 33.0, 32.0, 31.3, 29.9, 29.8, 29.7, 29.7, 29.6, 29.4, 29.3, 28.7, 28.6, 26.0, 22.8, 21.6, 21.5, 20.3, 20.1, 14.2, 13.9. IR (neat): 2959, 2926, 2873, 2855, 1734, 1650, 1457, 1419, 1375, 1250, 1230, 1174, 1060, 1020 cm^−1^. MS (ESI) calcd. for C_20_H_39_NO_3_ [M + H^+^]: 342.2; found: 342.3.

*Dodecyl 3-(N-butylacetamido)propanoates*: Following **GP2**, 3-(*N*-butylacetamido)propionic acid (1.0 equiv., 10.0 g, 53.40 mmol) was dissolved in toluene (100 mL), then 1-dodecanol (1.0 equiv., 9.9 g, 53.40 mmol) and *p-*toluenesulfonic acid (0.022 equiv., 0.2 g, 1.00 mmol) were added to the solution. After the work-up the title compound was obtained as a yellowish oil (15.1 g, 80% yield). ^1^H NMR (600 MHz, CDCl_3_) δ 4.11–4.02 (m, 2H), 3.64–3.54 (m, 2H), 3.34–3.21 (m, 2H), 2.66–2.51 (m, 2H), 2.12 (MinR, s, 1H), 2.08 (MajR, s, 2H), 1.65–1.58 (m, 2H), 1.58–1.46 (m, 2H), 1.26 (s, 20H), 0.96–0.86 (m, 6H). ^13^C NMR (151 MHz, CDCl_3_) δ 172.4, 171.2, 170.5, 170.3, 65.3, 64.9, 49.6, 45.4, 44.3, 42.4, 34.0, 33.0, 32.0, 31.3, 29.9, 29.8, 29.7, 29.7, 29.7, 29.6, 29.4, 29.3, 28.7, 28.6, 26.0, 22.8, 21.5, 20.3, 20.1, 14.2, 13.9. IR (neat): 2961, 2926, 2872, 2855, 1735, 1650, 1458, 1420, 1375, 1251, 1232, 1174, 1060, 1022 cm^−1^. MS (ESI) calcd. for C_21_H_41_NO_3_ [M + H^+^]: 356.3; found: 356.4.

#### 3.2.3. Characterization of Synthesized Compounds

IR spectra were recorded using a Lumex (Saint Petersburg, Russia) InfraLUM FT-08 Fourier transformation spectrometer equipped with a diamond-crystal disturbed total internal reflection (DTIR) accessory. Measurements were performed in the 1000–3100 cm^−1^ spectral range at a resolution of 4 cm^−1^. The compounds under analysis were applied as a thin film, completely covering the crystal surface. The obtained spectra were processed using OMNIC 8.3 software (Thermo Fisher Scientific, Waltham, MA, USA). All IR spectra obtained are in Supplementary Figure S1.

The ^1^H and ^13^C NMR spectra were recorded on a Bruker Avance III spectrometer (Bruker, Germany) operating at frequencies of 600.1 MHz and 150.9 MHz, respectively. The samples were dissolved in deuterated chloroform (CDCl_3_) and transferred to 178 mm DURAN Group NMR vials with diameters of 4.95 mm and 4.2 mm. The spectra obtained were processed using MestReNova software, version 16.0.0 (Mestrelab Research S.L.U., Santiago de Compostela, Spain). All NMR spectra obtained are in Supplementary Figure S2.

Mass spectra were obtained using high-performance liquid chromatography coupled with tandem mass spectrometry (HPLC-MS/MS) on an S6000Plus chromatograph (Achrom-Tech, Beijing, China), which was equipped with a triple-quadrupole detector Sciex 3500. The target compounds were separated using an Acclaim™ 120 Dionex bonded silica-based column (150 mm × 2.1 mm, 3 µm, C18) under the following chromatographic conditions: mobile phase (0.1% trifluoroacetic acid in acetonitrile); column temperature (35 °C); sample volume (10 µL); and flow rate (0.27 mL/min). An electrospray ionization (ESI) source was used for sample ionization. Ions were detected in multiple reaction monitoring (MRM) mode. The obtained spectra were analyzed using SCIEX OS 4.2 software (AB Sciex Pte. Ltd., Marlborough, MA, USA). The uncertainty for both precursor and fragment mass determination was ±0.300 Da. All mass spectra obtained are in Supplementary Figure S3.

#### 3.2.4. Preparing of Textile Samples

Samples of white calico (Shuiskie Sitsy Ltd., Shuya, Russia) in the shape of a half of a 90 mm-diameter Petri dish were used in the tests. The fabric was immersed in an alcohol solution until fully saturated to achieve the desired concentration of the tested substance. Preliminary laboratory tests showed that a soaking time of five minutes was optimal for all compounds, regardless of their concentration. The concentrations of substances on the textile (*C*, g/m^2^) were calculated using the following formula:

(1) $$C=\frac{m_{s}\cdot X}{S\cdot 100\%},$$

where $m_{s}$ is the mass (in g) of the alcohol solution absorbed by the textile (the maximum absorbency of alcohol solutions containing different concentrations of test substances was previously determined under laboratory conditions);

X—substance content in the solution (*w*/*w*), %;
*S*—textile surface area, m^2^.

After soaking in the solution, the textile samples were dried for 3 h at 22–25 °C. This time frame was chosen based on preliminary findings, which have indicated that the mass of all samples becomes consistent during this period. The impregnated textiles were used on the day of their preparation.

The active component concentrations on the tissues were selected based on the findings of our earlier investigations [11]. The standard deviation of the concentration after impregnation was <5% (*n* = 10).

#### 3.2.5. Choice Trial

Taiga ticks were collected along forest roads and forest edges near the Taltsa River, Irkutsk Oblast, Russia (52°00′49.2″ N 104°40′26.0″ E), in May–June 2025, during a warm and dry weather period (air temperatures of 13–23 °C and relative humidity of 50–70%), under mixed coniferous broad-leaved forest canopy. Ticks were collected on white cotton cloth (flags) with no chemicals applied, and transported to the laboratory in a damp gauze tape on the same day they were collected. Female ticks were identified by their body size and by the presence of red alloscuta on their backs [22]. The identification of ticks was carried out after the experiment by means of microscope in laboratory conditions. Ticks were used on the day of collection or the next day. Wet gauze was used to store them at 4–8 °C. One to two hours before testing, ticks were placed at room temperature.

Behavioral bioassays were conducted at 23–25 °C and 50–60% relative humidity. There was a choice between untreated and treated calico fabrics for ticks. In this experiment, two 31.8 cm^2^ semi-circular pieces of calico fabric were layered into a 63.6 cm^2^ Petri dish lid. One fabric piece was soaked in a solution that contained the active ingredient diluted in ethanol, while the other section (control) was soaked in pure ethanol. They were allowed to dry for 3 h at room temperature. Five ticks were then placed along the line formed by the intersection of the treated and untreated fabrics. After ticks were introduced into the test area, their distribution with respect to the line was recorded every 5 min, up to 30 min total. The experiment was repeated six times (number of ticks *n* = 30).

#### 3.2.6. Data Analysis

During the experiment, the location of the tick on one of two fabric samples was determined. If the tick was on the repellent-treated sample at the 30 min mark, the result was recorded as “0”; if it was on the untreated sample, the result was recorded as “1”. The repellency was then calculated using a dose–response curve to determine the relative frequency of the tick being found on the untreated half.

Root mean square deviation of frequencies was used as a measure of variability. Statistical comparisons were performed using Fisher’s exact test: first, to compare materials containing the repellent with the control, and second, to assess all synthesized compounds against the commercial standard IR3535^®^. These calculations were based on the formulas presented in [23] and performed using OriginPro software 2024 (OriginLab Corp., Northampton, MA, USA). A significance level of 0.05 was adopted for all calculations.

## 4. Conclusions

In this study, we synthesized and investigated a series of alkyl 3-(*N*-butylacetamido)propanoates with alkyl chain lengths from C1 to C12. The esterification of 3-(*N*-butylacetamido)propionic acid was found to proceed differently for alcohols with alkyl groups of ≤C4 and ≥C5. The existence of two rotamers in solution was demonstrated for all synthesized compounds, which may be due to the high energy barrier for rotation about the amide bond. Mass spectrometric studies revealed that all synthesized compounds exhibit the same fragment (daughter) ions at *m*/*z* 170 (C_9_H_17_NO_2_^+^), 128 (C_7_H_14_NO^+^), and 86 (C_5_H_12_N^+^), suggesting a shared fragmentation mechanism. Therefore, chromatography coupled with tandem mass spectrometry is the optimal method for analyzing these compounds.

All synthesized compounds were evaluated for repellent activity *against Ixodes persulcatus*. It was shown that there is no pattern linking the length of the alkyl radical to biological activity for the homologous series under consideration. At the same time, however, a statistically significant influence of the alkyl fragment of the molecule on repellent activity was established. The methyl, propyl and butyl esters exhibited the best repellent properties, with a repellent effect against the taiga tick that is approximately six times greater than that of the commercially available IR3535^®^. Therefore, the synthesized compounds represent promising candidates for repellent development.

## Figures and Tables

**Figure 1:**
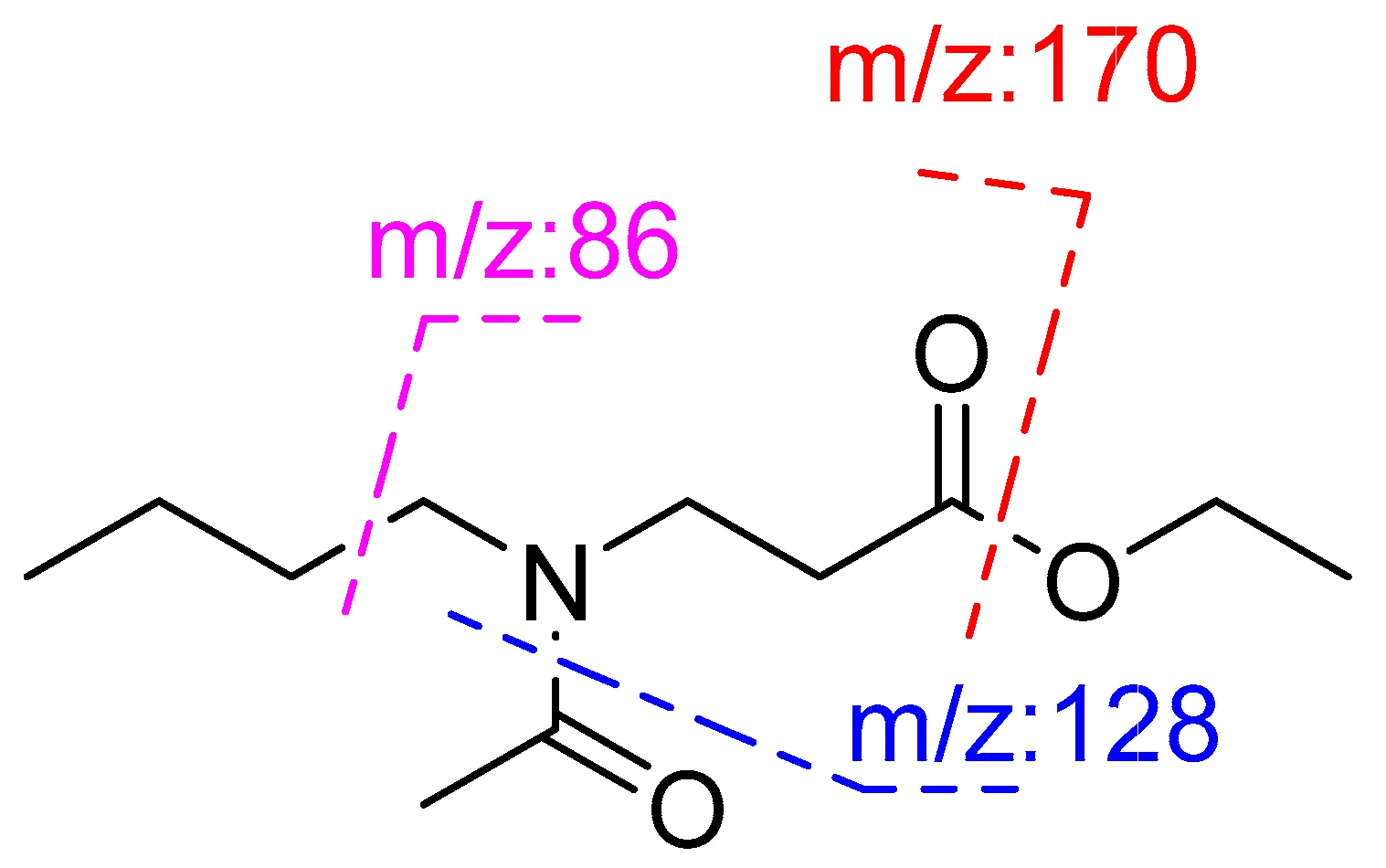
Common fragmentation ions of alkyl 3-(*N*-butylacetamido)propanoates.

**Figure 2:**
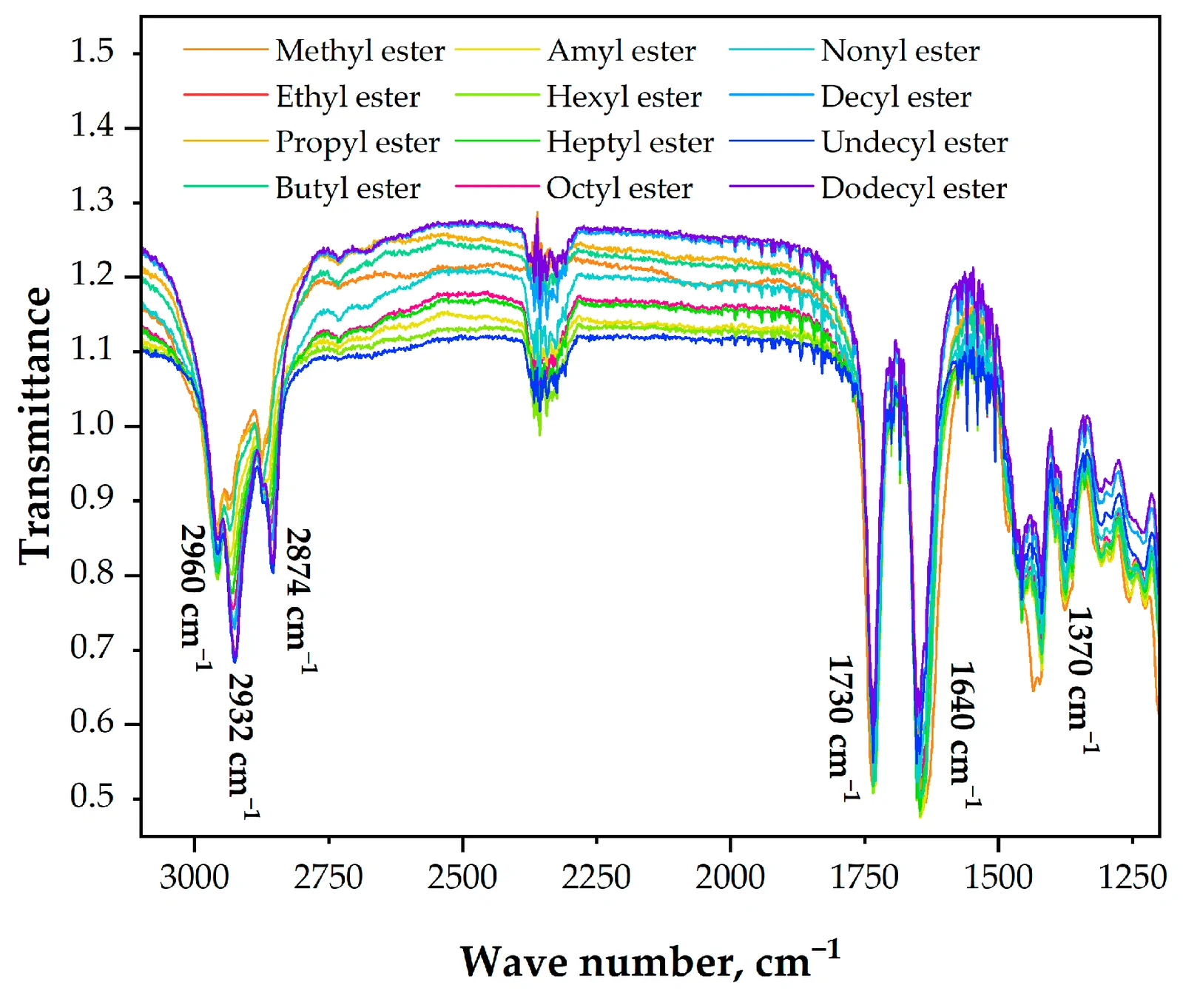
IR spectra of alkyl 3-(*N*-butylacetamido)propanoates.

**Figure 3:**
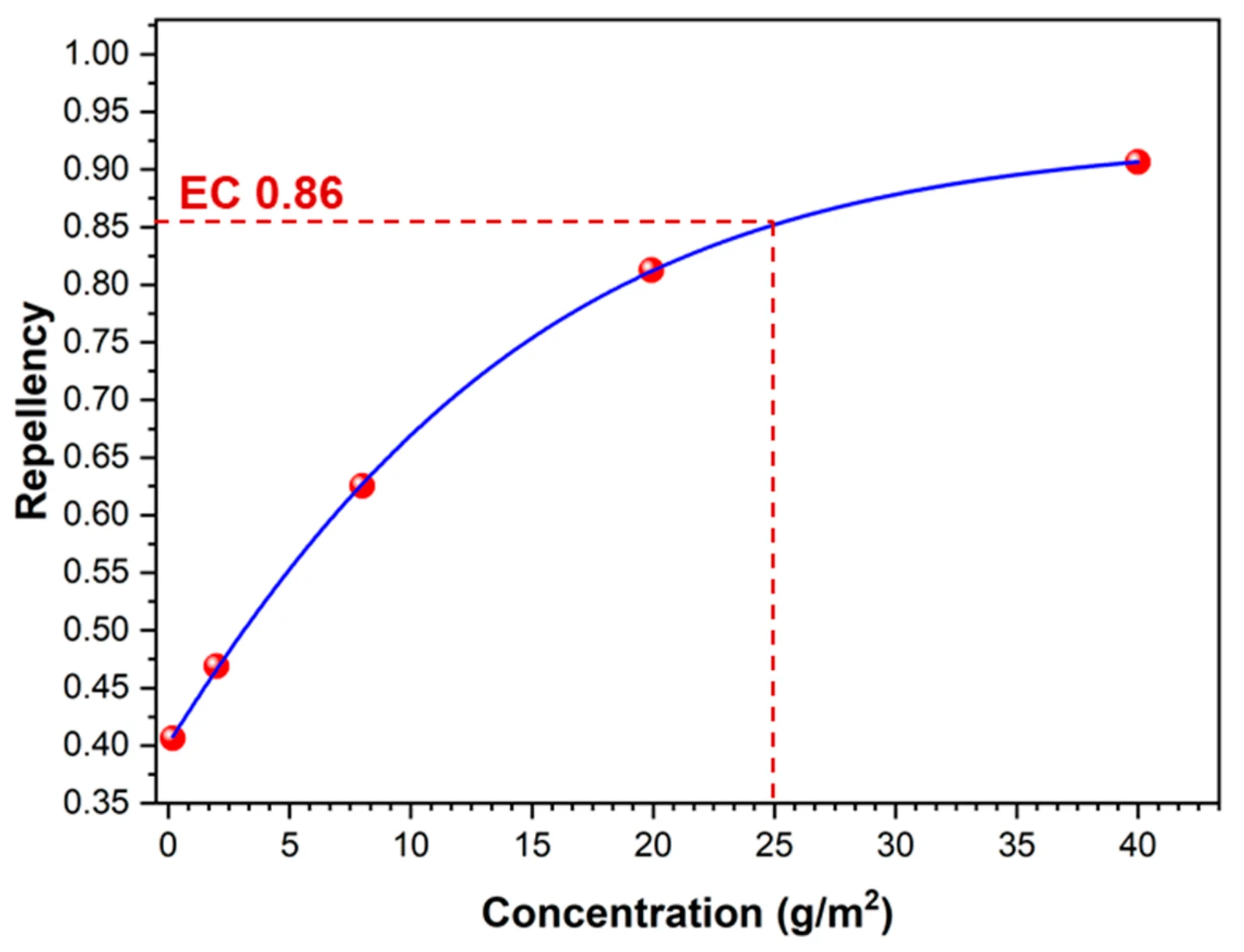
Dose–response curve showing the dependence of the repellent activity of the commercially available compound IR3535^®^ (*n* = 30).

**Figure 4:**
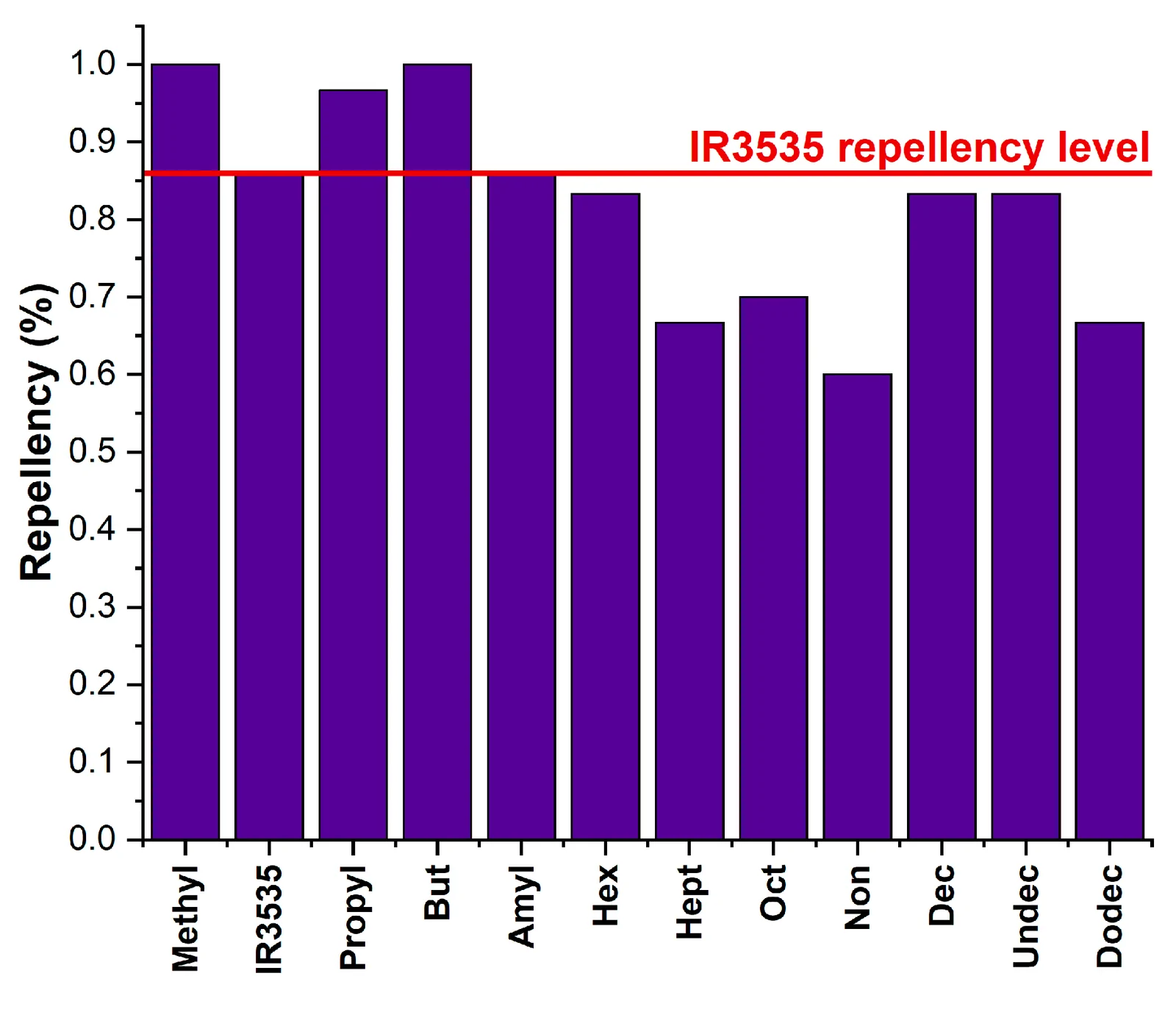
Comparison of repellent activity against the taiga tick *Ixodes persulcatus* between synthesized derivatives and the commercially available IR3535^®^. The concentration of all substances was 25 g/m^2^.

**Figure 5:**
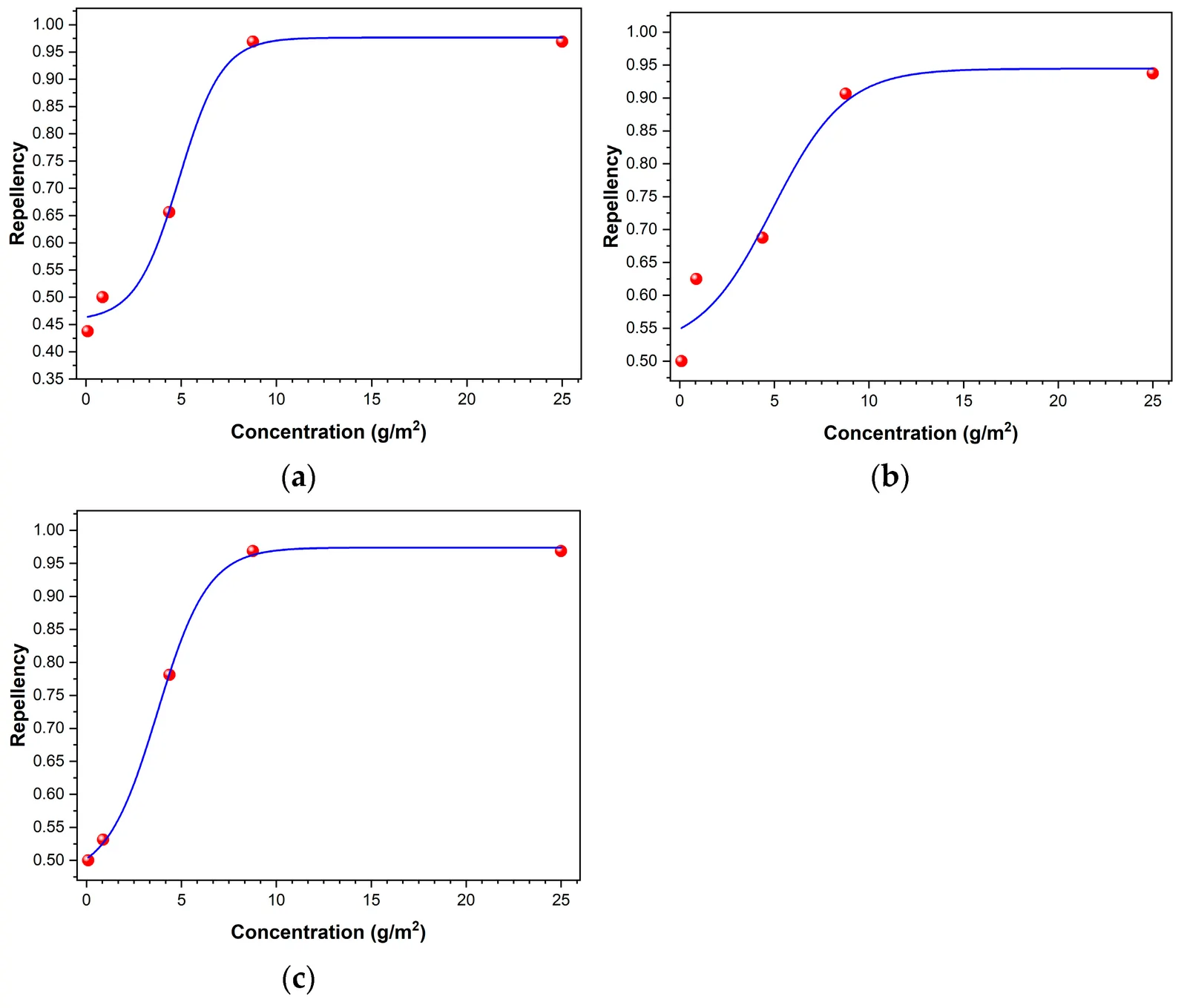
Dose–response curves showing the dependence of the repellent activity of substances on concentration, as tested on the taiga tick *Ixodes persulcatus*. (**a**) methyl 3-(*N*-butylacetamido)propanoate; (**b**) propyl 3-(*N*-butylacetamido)propanoate; (**c**) butyl 3-(*N*-butylacetamido)propanoate. For all compounds, 30 ticks were used to determine each data point.

**Figure 6:**
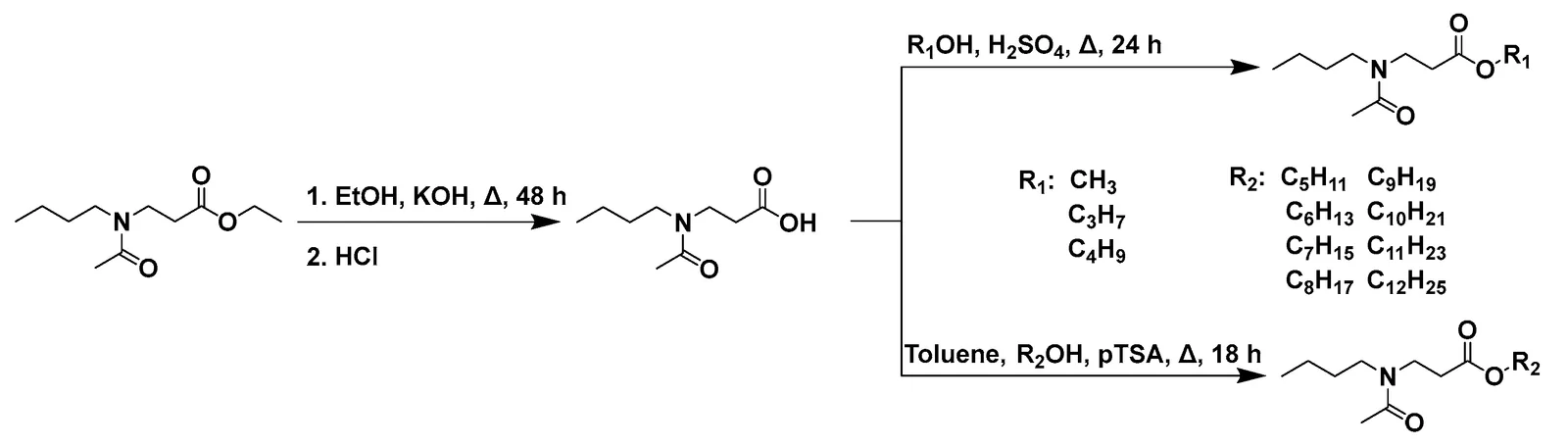
Two different pathways of alkyl 3-(*N*-butylacetamido)propanoates synthesis.

**Table 1:** Selected signals (in ppm) from ^1^H NMR spectra.

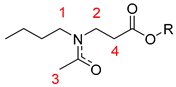	1	2	3		4
R			MinR	MajR	
Methyl	3.33–3.24	3.62–3.55	2.15	2.10	2.63–2.55
IR3535^®^ (Ethyl)	3.32–3.24	3.61–3.54	2.16	2.11	2.62–2.55
Propyl	3.34–3.27	3.63–3.56	2.20	2.14	2.64–2.55
Butyl	3.32–3.21	3.61–3.51	2.10	2.06	2.64–2.52
Amyl	3.37–3.26	3.66–3.55	2.20	2.16	2.67–2.53
Hexyl	3.39–3.27	3.67–3.56	2.26	2.20	2.69–2.56
Heptyl	3.32–3.21	3.61–3.51	2.11	2.06	2.64–2.50
Octyl	3.32–3.23	3.60–3.53	2.11	2.06	2.62–2.53
Nonyl	3.33–3.23	3.61–3.54	2.11	2.07	2.63–2.54
Decyl	3.32–3.22	3.64–3.51	2.11	2.07	2.63–2.52
Undecyl	3.33–3.23	3.61–3.54	2.11	2.07	2.63–2.53
Dodecyl	3.34–3.21	3.64–3.54	2.12	2.08	2.66–2.51

**Table 2:** Selected signals (in ppm) from ^13^C NMR spectra.

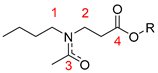	1		2		3		4	
R	MajR	MinR	MajR	MinR	MajR	MinR	MajR	MinR
Methyl	49.6	45.3	42.4	44.2	172.7	171.5	170.6	170.3
IR3535^®^ (Ethyl)	49.6	45.4	42.4	44.3	172.4	171.2	170.5	170.3
Propyl	49.6	45.3	42.4	44.3	172.4	171.2	170.5	170.2
Butyl	49.6	45.3	42.4	44.3	172.4	171.2	170.5	170.3
Amyl	49.6	45.3	42.4	44.3	172.4	171.2	170.5	170.2
Hexyl	49.6	45.3	42.4	44.3	172.3	171.2	170.5	170.2
Heptyl	49.6	45.4	42.4	44.3	172.4	171.2	170.5	170.3
Octyl	49.6	45.3	42.4	44.3	172.4	171.2	170.5	170.3
Nonyl	49.6	45.4	42.4	44.3	172.4	171.2	170.5	170.3
Decyl	49.6	45.4	42.4	44.3	172.4	171.2	170.5	170.3
Undecyl	49.6	45.4	42.4	44.3	172.4	171.2	170.5	170.3
Dodecyl	49.6	45.4	42.4	44.3	172.4	171.2	170.5	170.3

**Table 3:** Rotamer ratio for the obtained alkyl 3-(*N*-butylacetamido)propanoates.

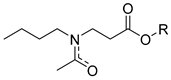 R	Minor Rotamer %	Major Rotamer %
Methyl	30.7	69.3
IR3535^®^ (Ethyl)	31.1	68.9
Propyl	30.7	69.3
Butyl	31.9	68.1
Amyl	31.0	69.0
Hexyl	30.0	70.0
Heptyl	31.5	68.5
Octyl	32.0	68.0
Nonyl	32.0	68.0
Decyl	32.8	67.4
Undecyl	32.6	67.4
Dodecyl	30.3	69.7

## Data Availability

The manuscript reports the complete dataset. If needed, the corresponding author can be contacted via email for further calculations.

## Supplementary Materials

The following supporting information can be downloaded at https://www.mdpi.com/article/10.3390/molecules31193570/s1: Figure S1: IR spectra; Figure S2: NMR spectra; Figure S3: MS spectra.

## Abbreviations

DEET	*N*,*N*-diethyl-3-methylbenzamide
HPLC-MS/MS	high-performance liquid chromatography–tandem mass spectrometry
IR	infrared
IR3535^®^	ethyl 3-(*N*-butylacetamido)propanoate
NMR	nuclear magnetic resonance
