# Supplementary material for: Synthesis, Characterization and Biological Activity of Alkyl 3-(N-Butylacetamido)propanoates Against Ixodid Ticks
Source: Molecules. 2026 Oct 8;31(19):3570. doi: 10.3390/molecules31193570 (PMC13666575; doi:10.3390/molecules31193570)
Supplement: Supplementary file 1 [file molecules-31-03570-s001.zip › Supplementary FigureS1_IRspectra.pdf]

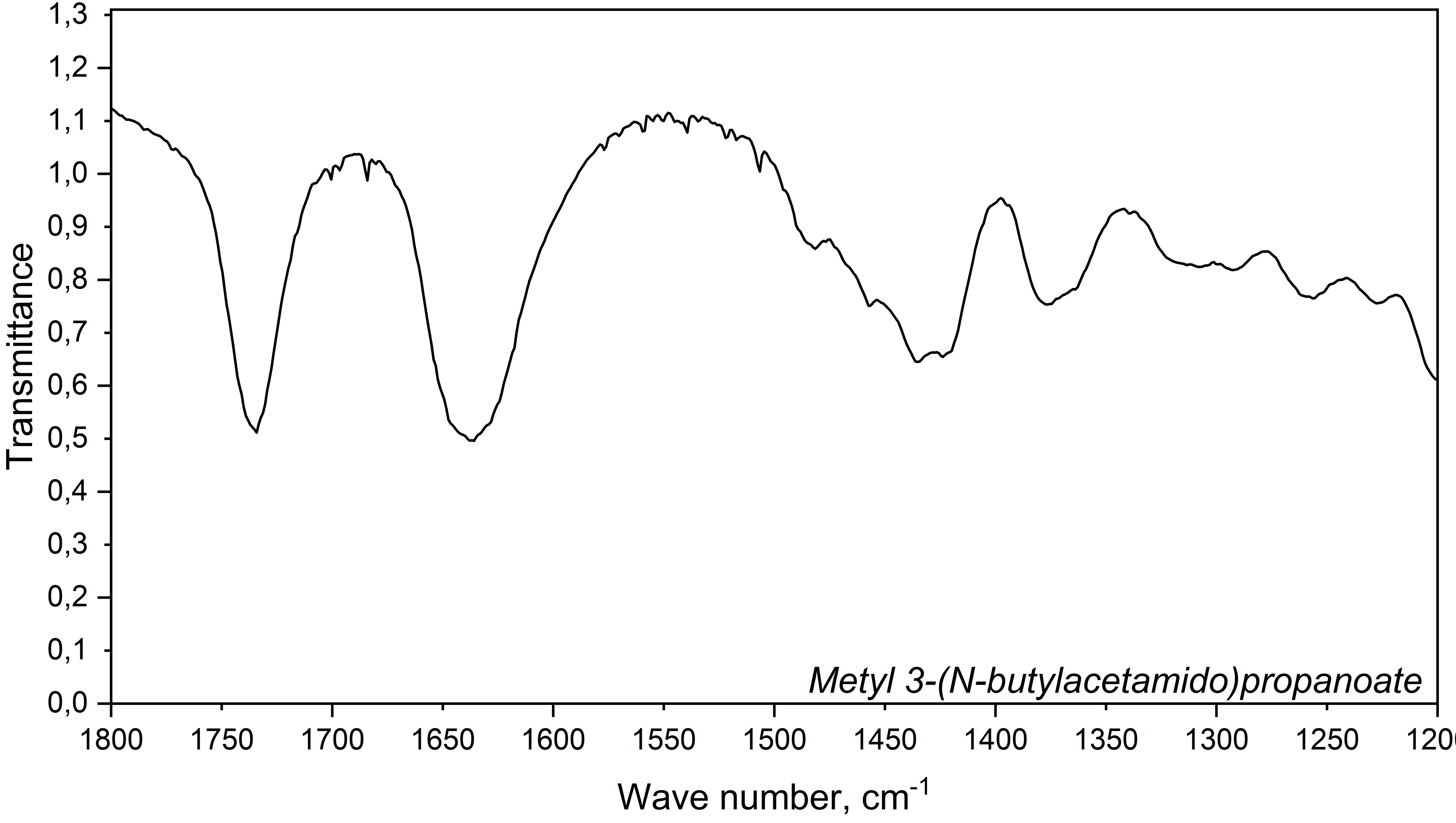

1,3
1,2
1,1
1,0
0,9
0,8
0,7
0,6
0,5
0,4
0,3
0,2
0,1
0,0
Transmittance
1800
1750
1700
1650
1600
1550
1500
1450
1400
1350
1300
1250
1200
Wave number, cm-1
Metyl 3-(N-butylacetamido)propanoate

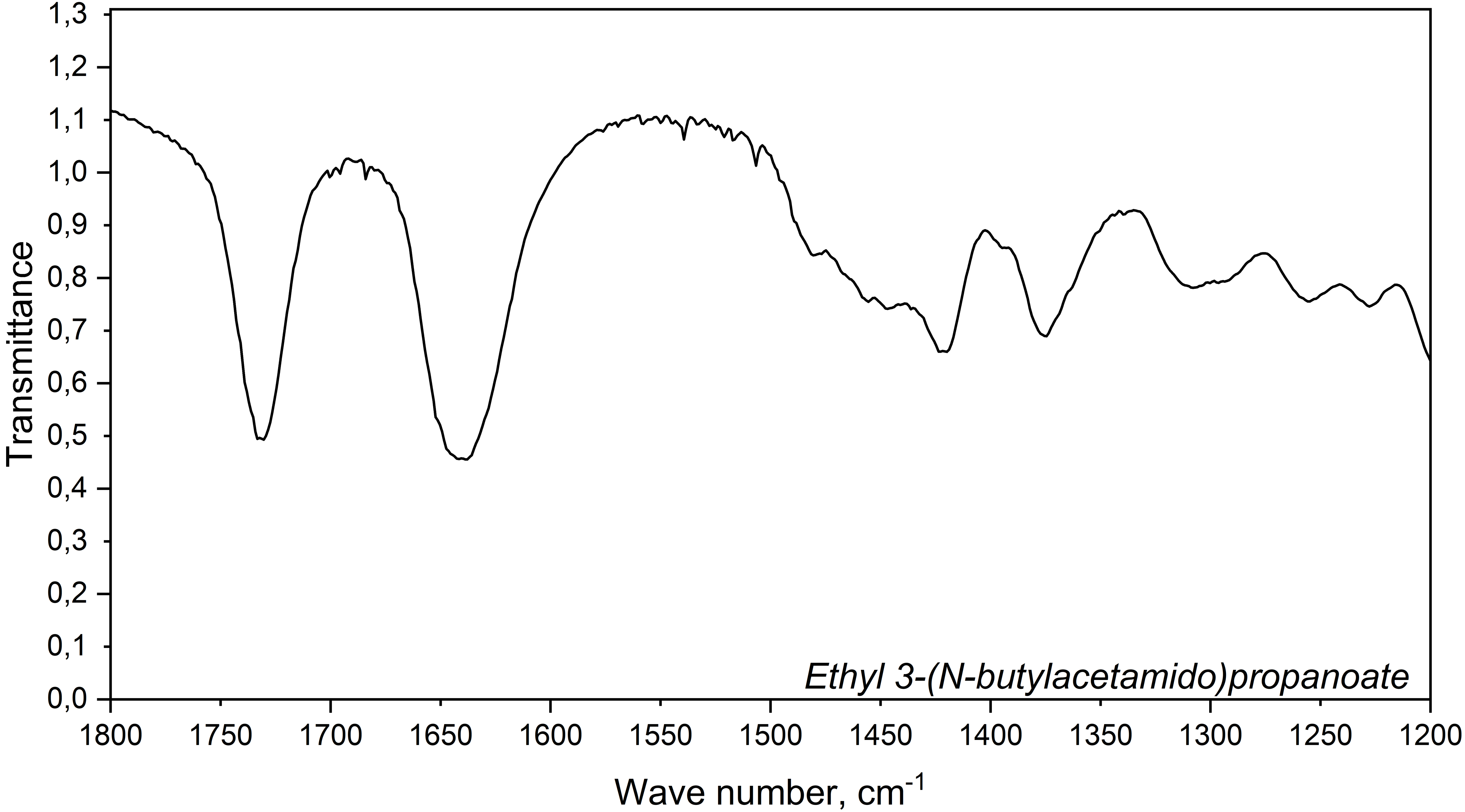

Transmittance
1,3
1,2
1,1
1,0
0,9
0,8
0,7
0,6
0,5
0,4
0,3
0,2
0,1
0,0
1800
1750
1700
1650
1600
1550
1500
1450
1400
1350
1300
1250
1200
Wave number, cm-1
Ethyl 3-(N-butylacetamido)propanoate

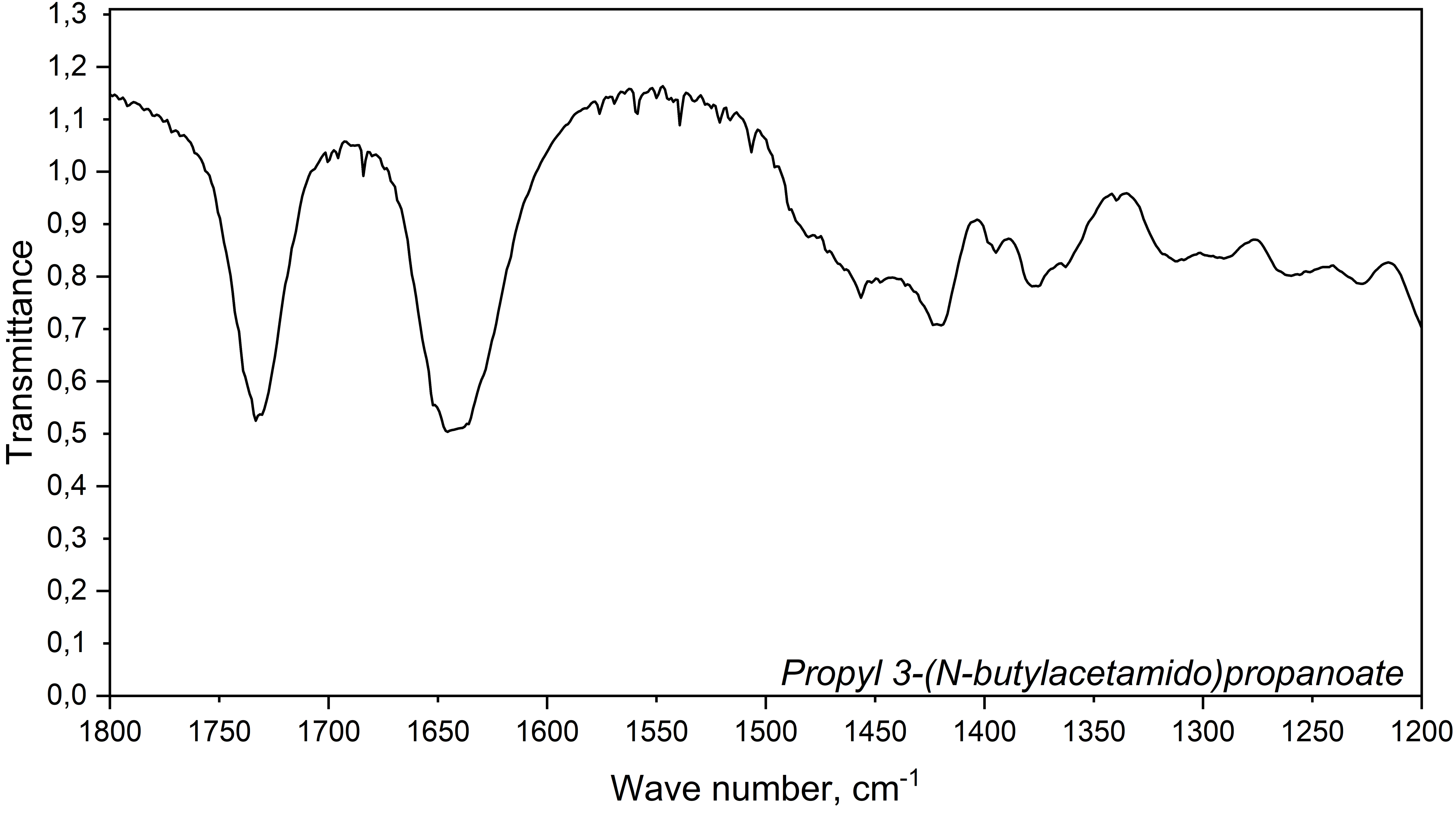

Transmittance
1,3
1,2
1,1
1,0
0,9
0,8
0,7
0,6
0,5
0,4
0,3
0,2
0,1
0,0
1800
1750
1700
1650
1600
1550
1500
1450
1400
1350
1300
1250
1200
Wave number, cm-1
Propyl 3-(N-butylacetamido)propanoate

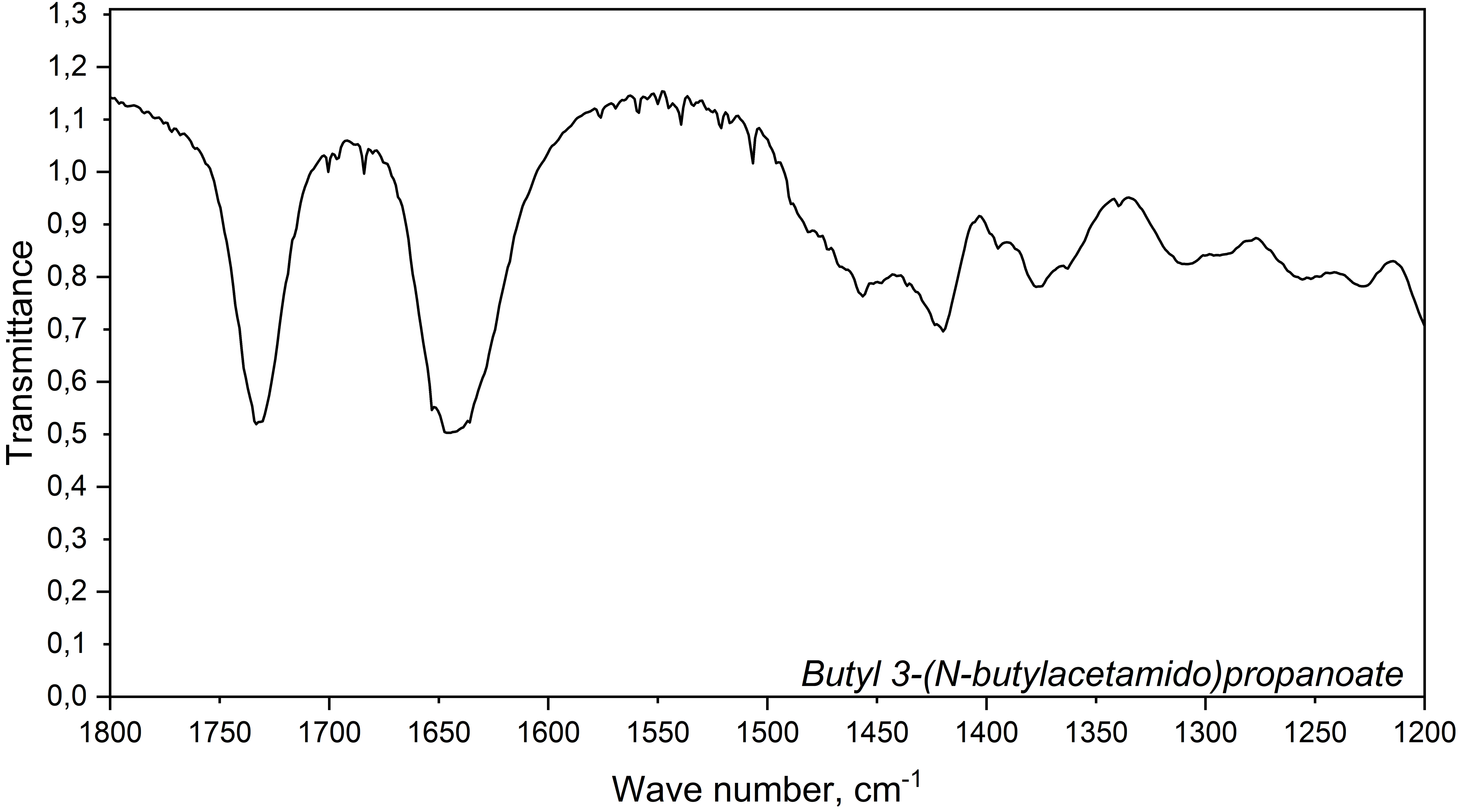

Transmittance
1,3
1,2
1,1
1,0
0,9
0,8
0,7
0,6
0,5
0,4
0,3
0,2
0,1
0,0
1800
1750
1700
1650
1600
1550
1500
1450
1400
1350
1300
1250
1200
Wave number, cm-1
Butyl 3-(N-butylacetamido)propanoate

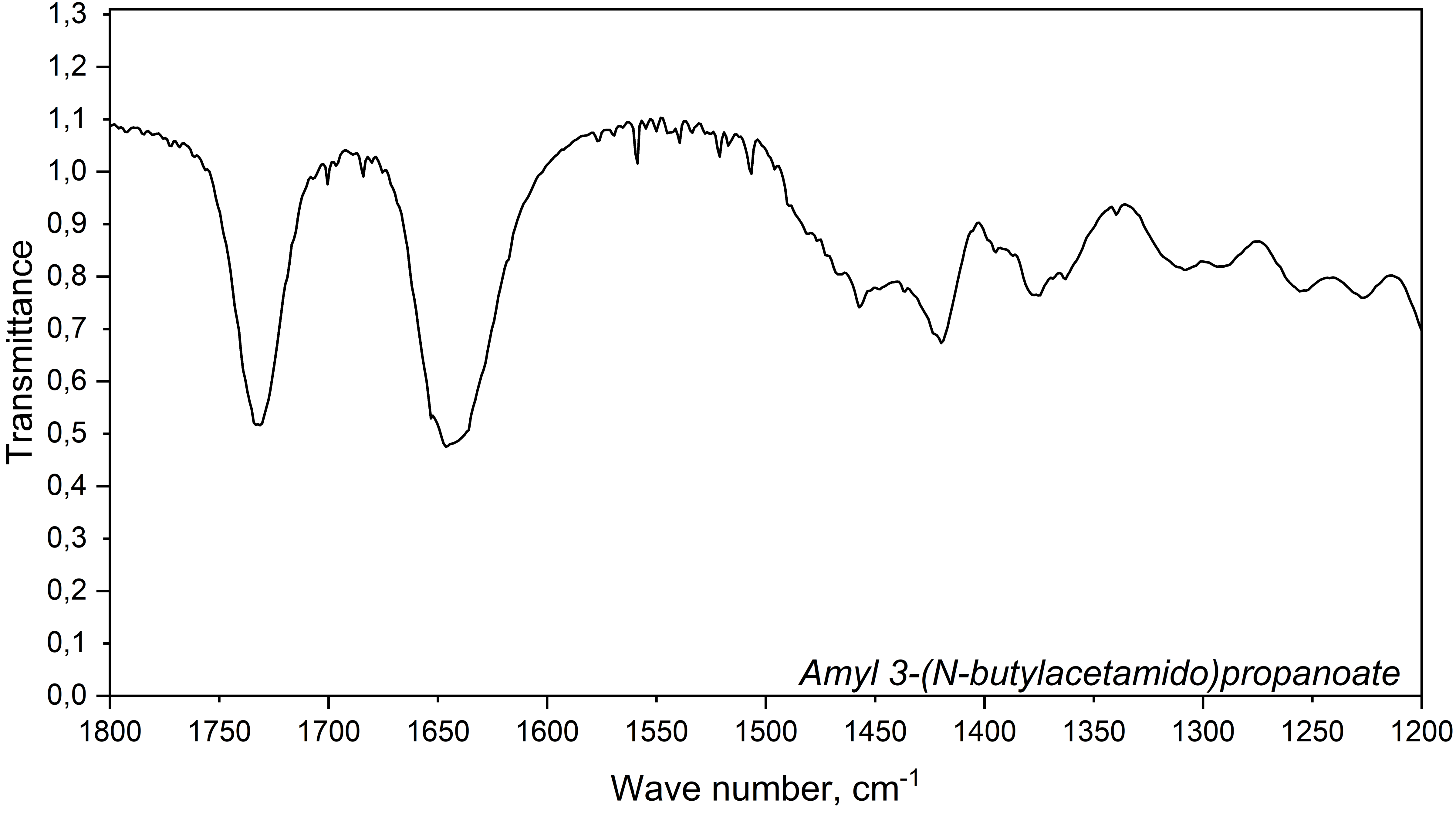

Transmittance
Wave number, cm^{-1}
Amyl 3-(N-butylacetamido)propanoate

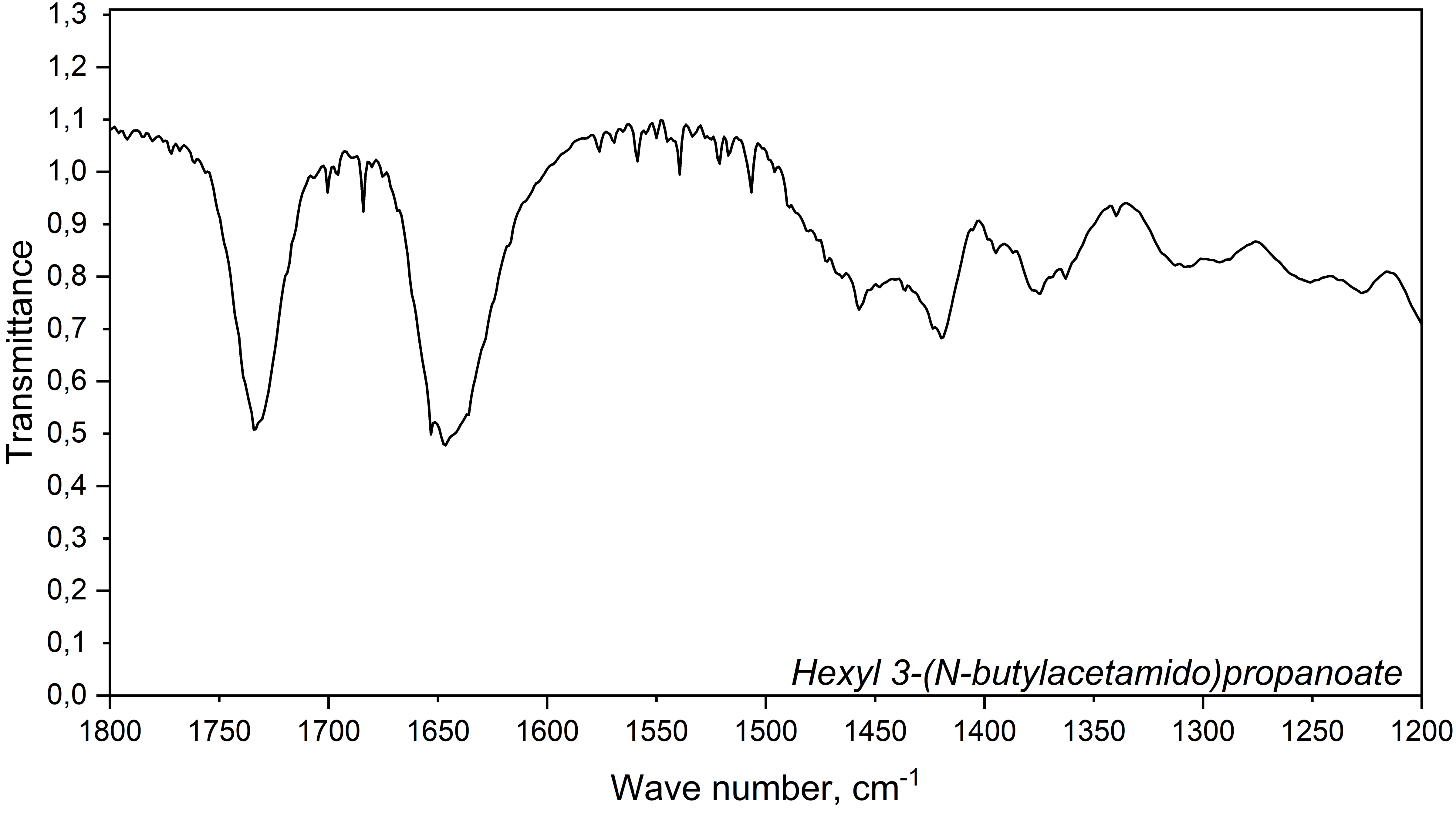

Transmittance
1,3
1,2
1,1
1,0
0,9
0,8
0,7
0,6
0,5
0,4
0,3
0,2
0,1
0,0
1800
1750
1700
1650
1600
1550
1500
1450
1400
1350
1300
1250
1200
Wave number, cm-1
Hexyl 3-(N-butylacetamido)propanoate

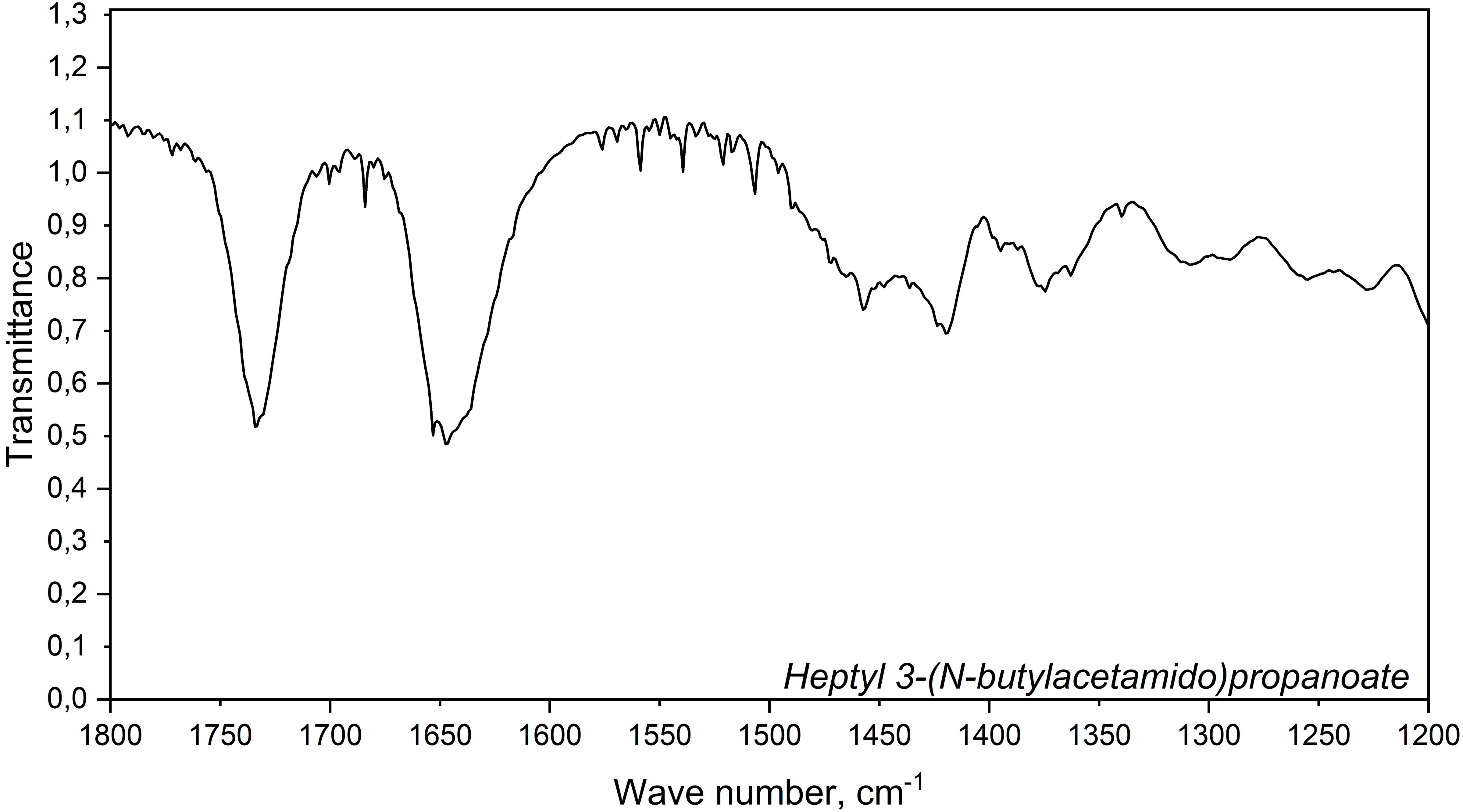

Heptyl 3-(N-butylacetamido)propanoate
Transmittance
1,3
1,2
1,1
1,0
0,9
0,8
0,7
0,6
0,5
0,4
0,3
0,2
0,1
0,0
1800
1750
1700
1650
1600
1550
1500
1450
1400
1350
1300
1250
1200
Wave number, cm-1

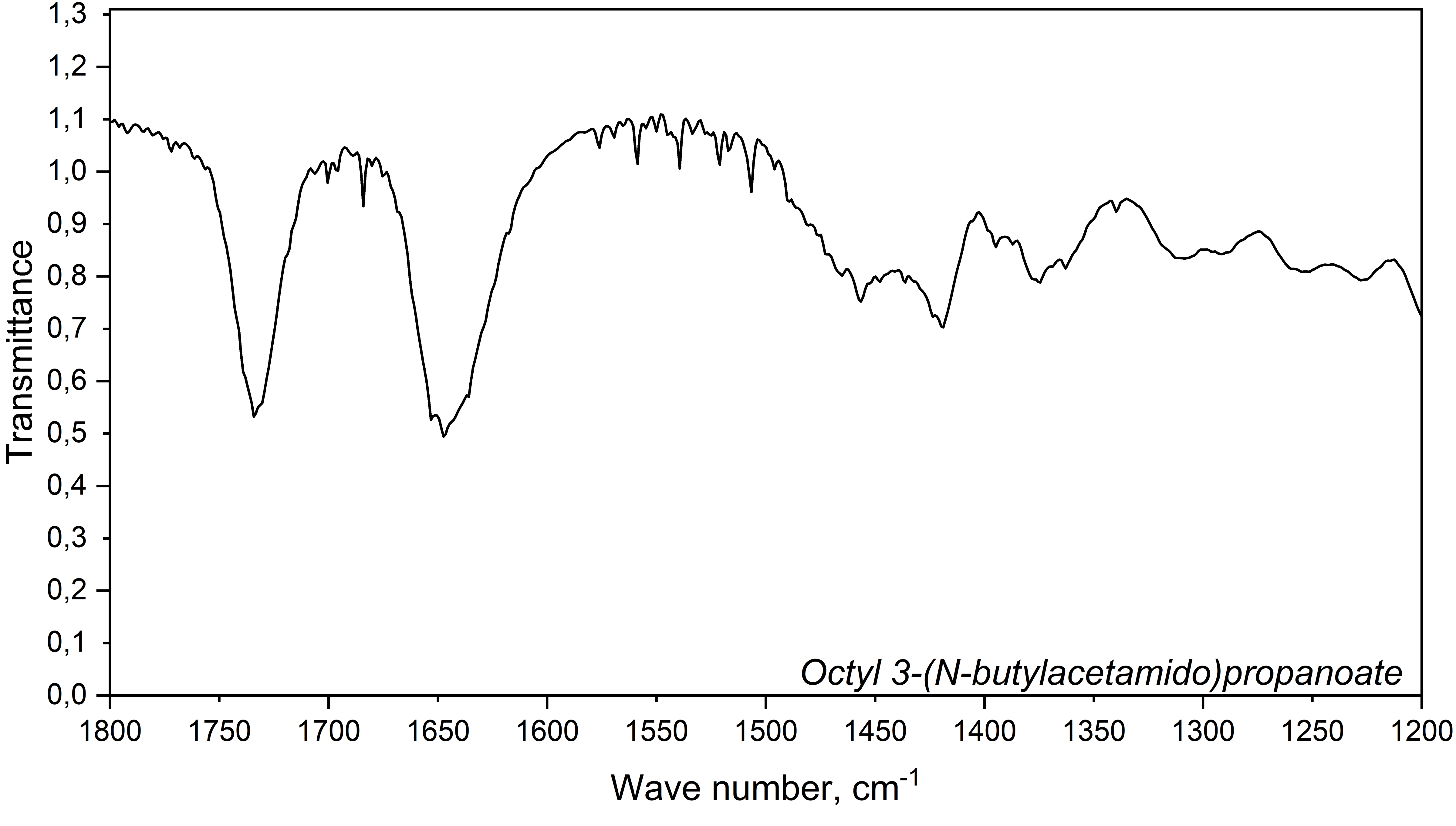

Transmittance
1,3
1,2
1,1
1,0
0,9
0,8
0,7
0,6
0,5
0,4
0,3
0,2
0,1
0,0
Octyl 3-(N-butylacetamido)propanoate
1800
1750
1700
1650
1600
1550
1500
1450
1400
1350
1300
1250
1200
Wave number, cm-1

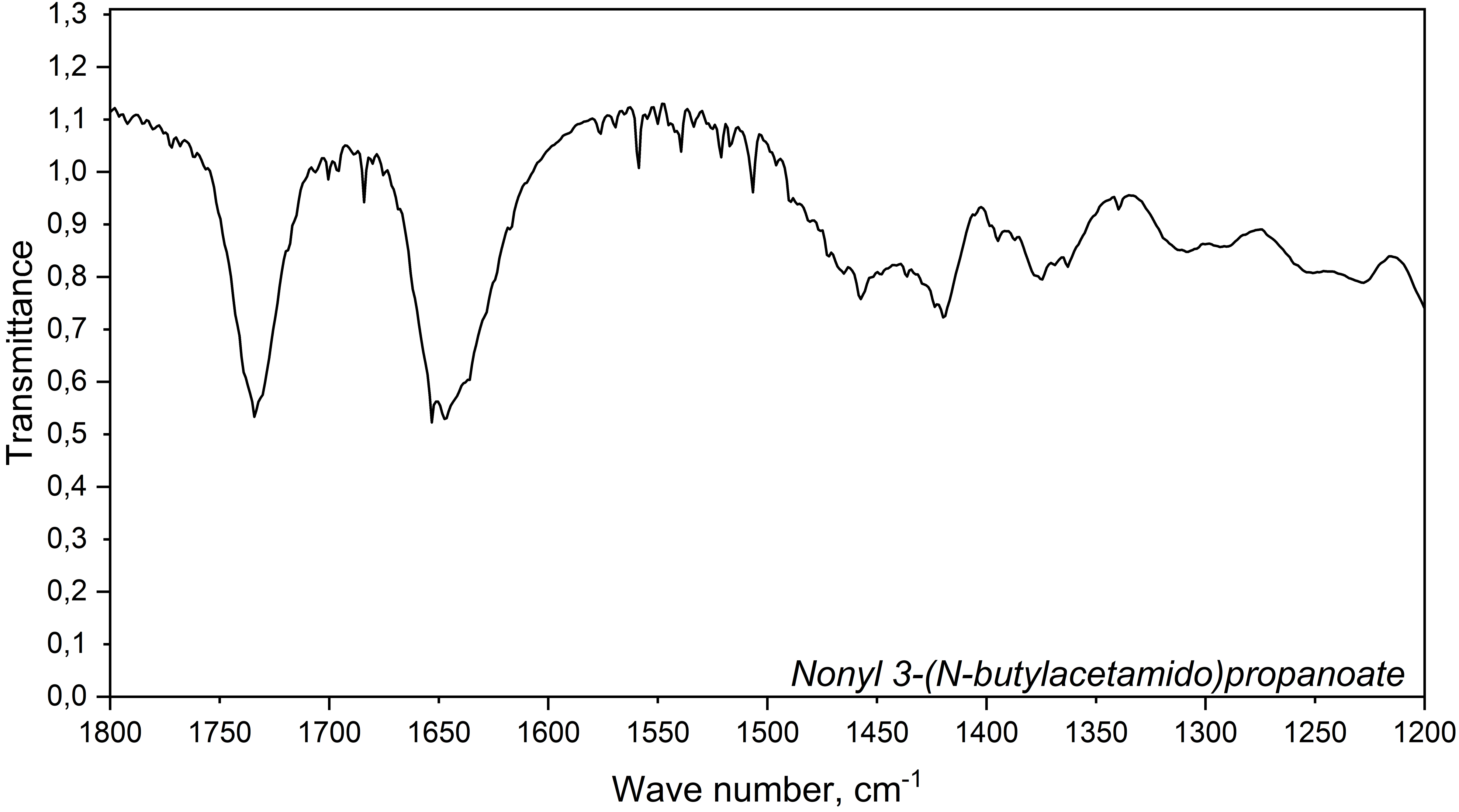

Transmittance
1,3
1,2
1,1
1,0
0,9
0,8
0,7
0,6
0,5
0,4
0,3
0,2
0,1
0,0
Nonyl 3-(N-butylacetamido)propanoate
1800
1750
1700
1650
1600
1550
1500
1450
1400
1350
1300
1250
1200
Wave number, cm-1

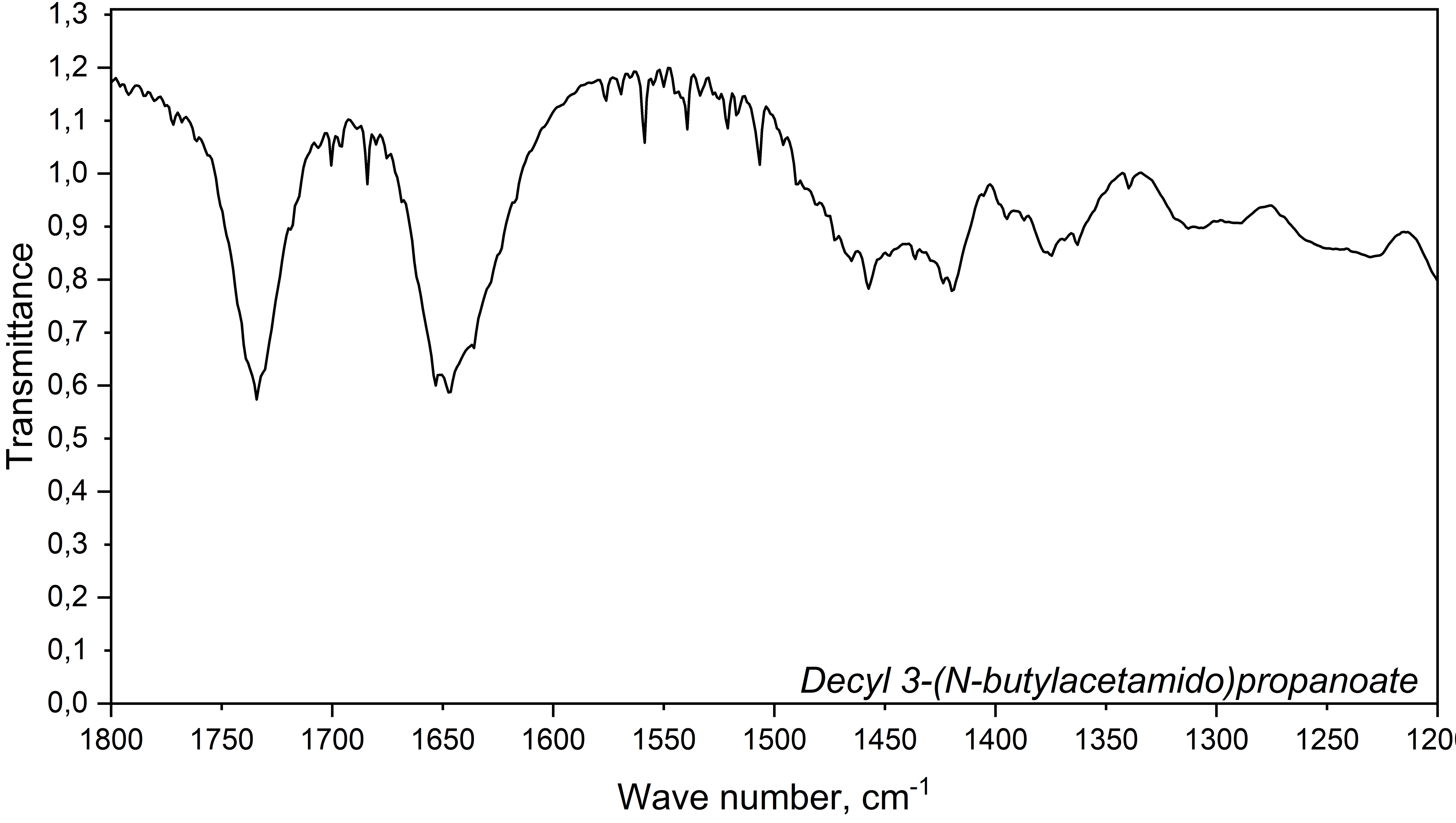

Transmittance
1,3
1,2
1,1
1,0
0,9
0,8
0,7
0,6
0,5
0,4
0,3
0,2
0,1
0,0
1800
1750
1700
1650
1600
1550
1500
1450
1400
1350
1300
1250
1200
Wave number, cm-1
Decyl 3-(N-butylacetamido)propanoate

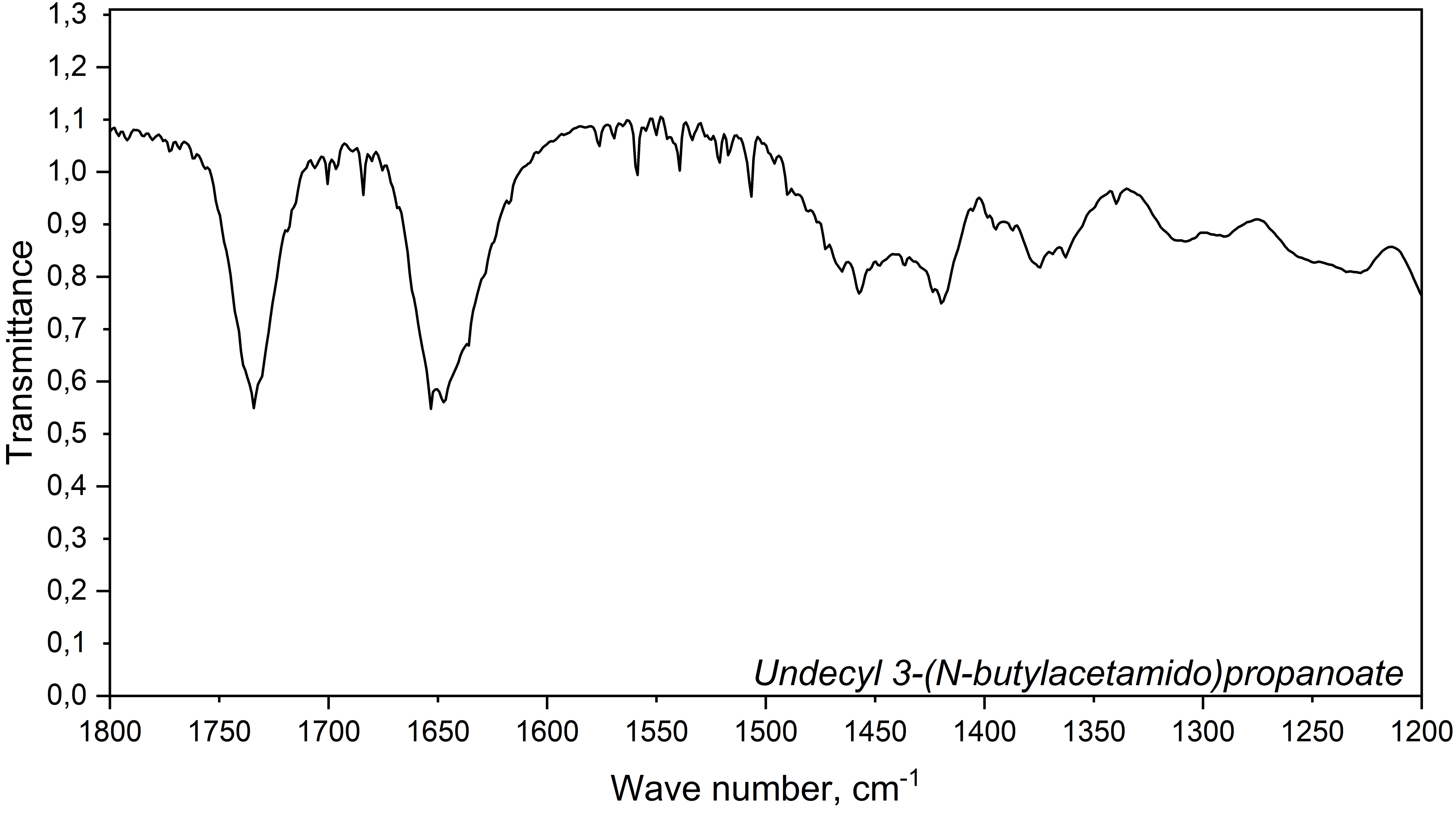
1,3
1,2
1,1
1,0
0,9
0,8
0,7
0,6
0,5
0,4
0,3
0,2
0,1
0,0
Transmittance
Undecyl 3-(N-butylacetamido)propanoate
1800
1750
1700
1650
1600
1550
1500
1450
1400
1350
1300
1250
1200
Wave number, cm-1

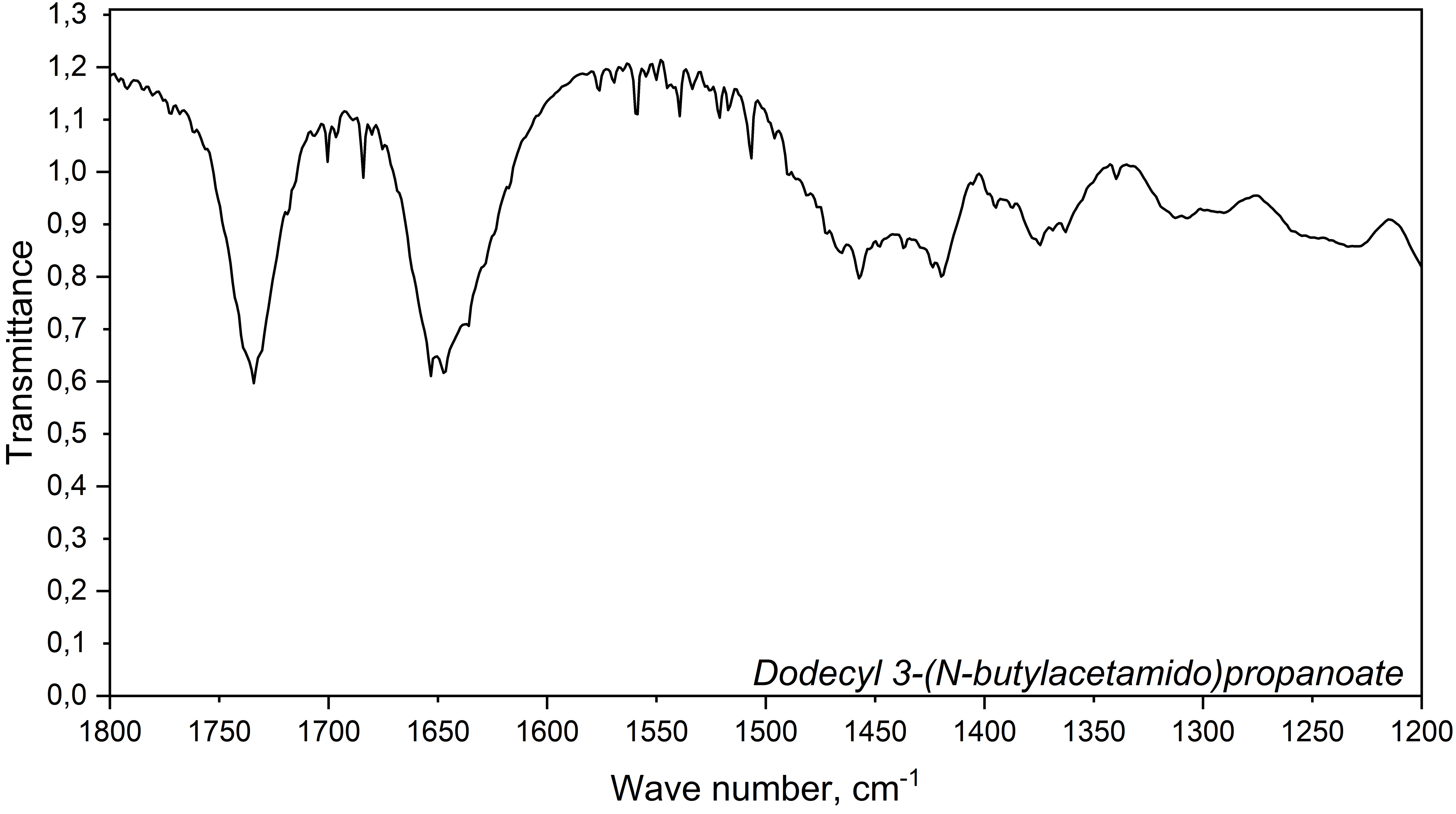
1,3
1,2
1,1
1,0
0,9
0,8
0,7
0,6
0,5
0,4
0,3
0,2
0,1
0,0
Transmittance
Dodecyl 3-(N-butylacetamido)propanoate
1800
1750
1700
1650
1600
1550
1500
1450
1400
1350
1300
1250
1200
Wave number, cm-1
