# Supplementary material for: Synthesis, Characterization and Biological Activity of Alkyl 3-(N-Butylacetamido)propanoates Against Ixodid Ticks
Source: Molecules. 2026 Oct 8;31(19):3570. doi: 10.3390/molecules31193570 (PMC13666575; doi:10.3390/molecules31193570)

*Methyl 3-(N-butylacetamido)propanoate*

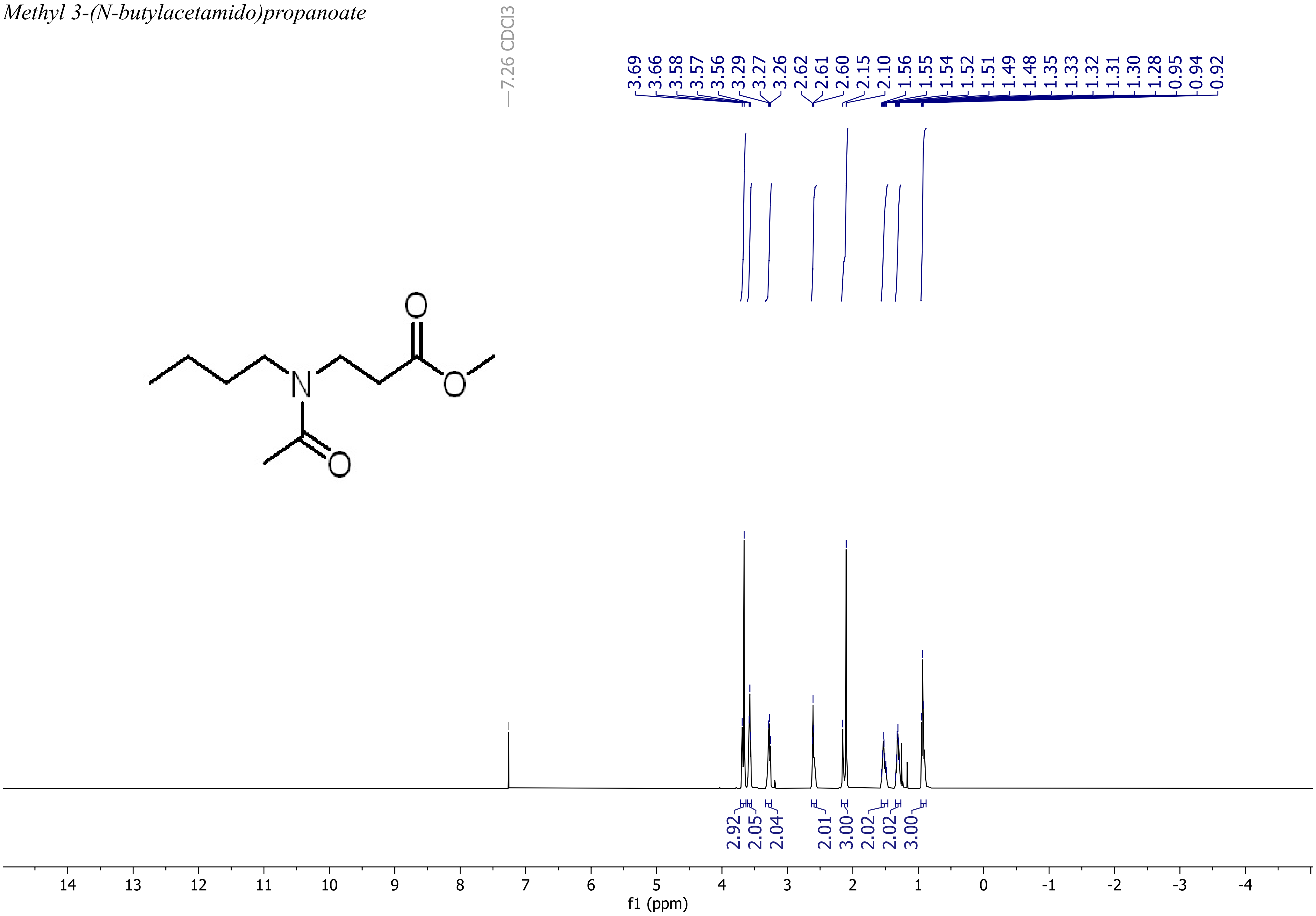

*Methyl 3-(N-butylacetamido)propanoate*

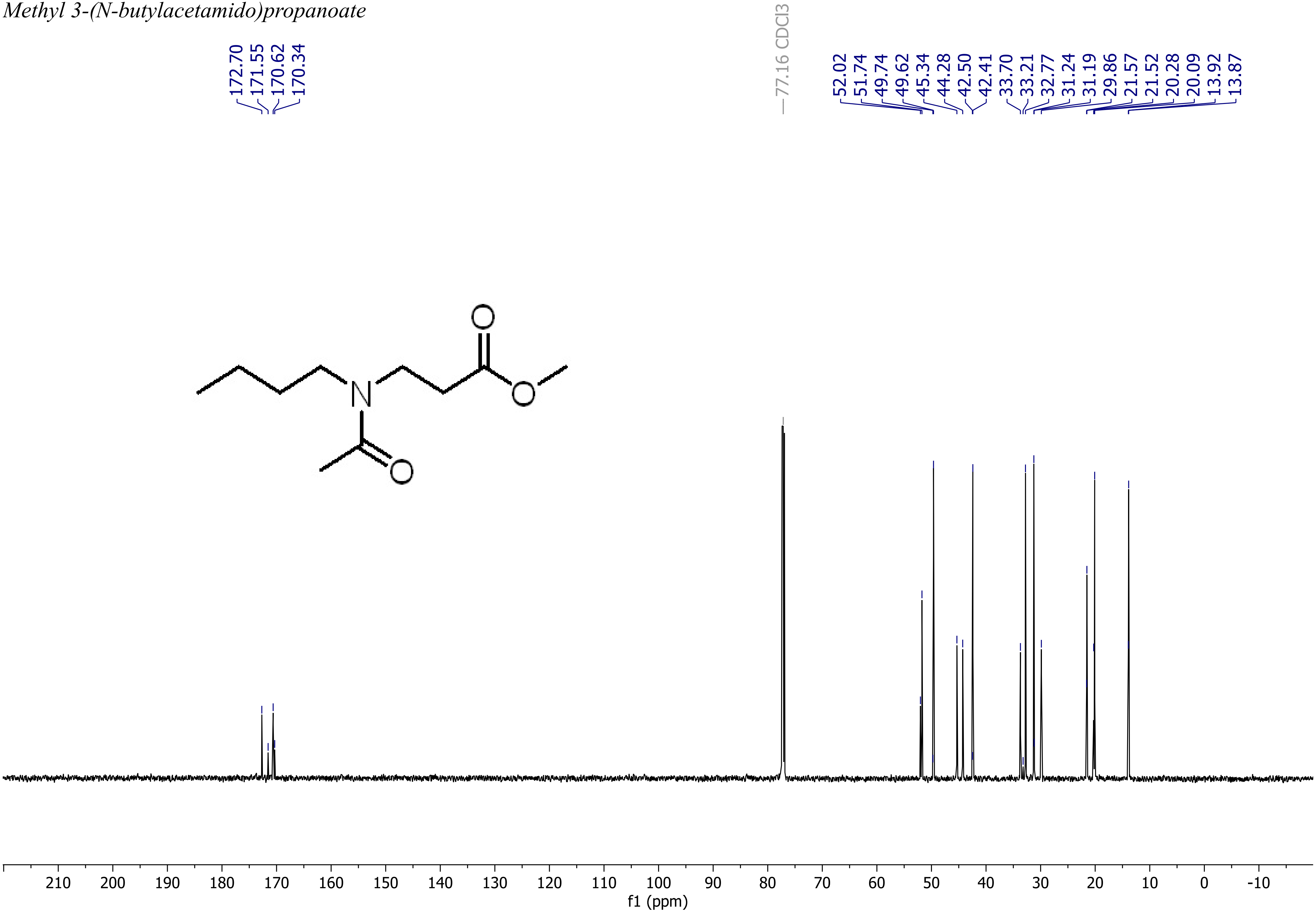

*Propyl 3-(N-butylacetamido)propanoate*

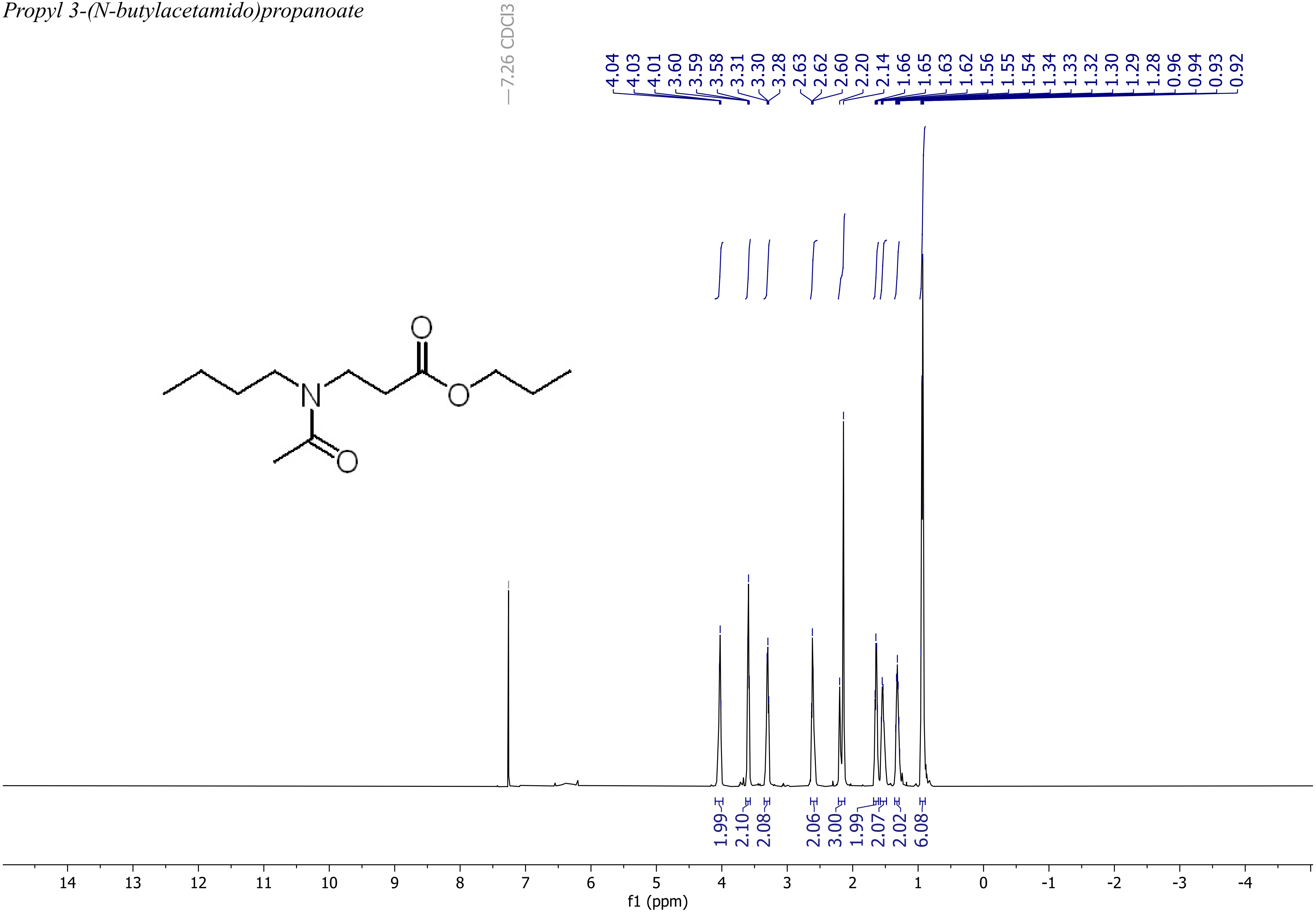

*Propyl 3-(N-butylacetamido)propanoate*

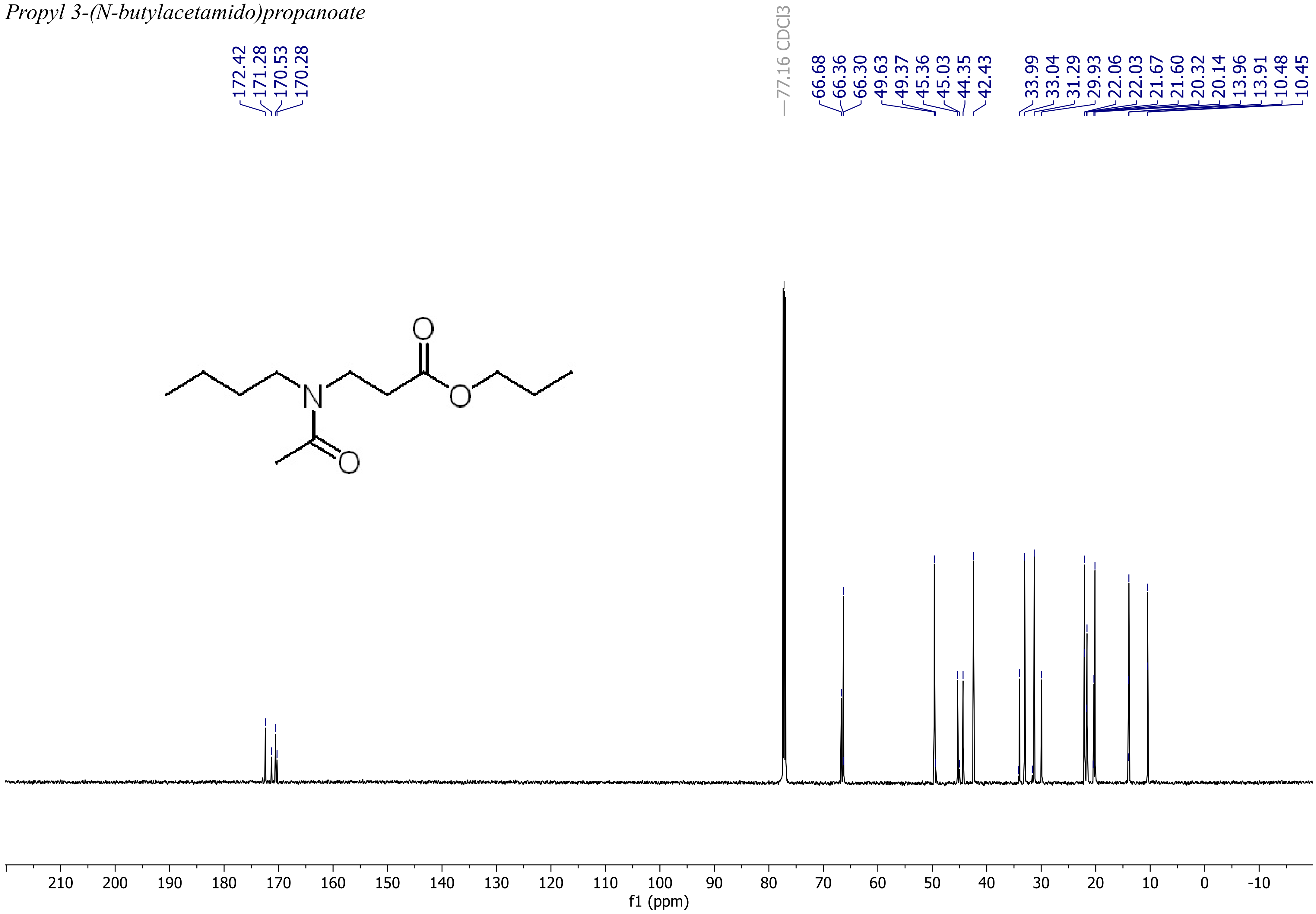

*Butyl 3-(N-butylacetamido)propanoate*

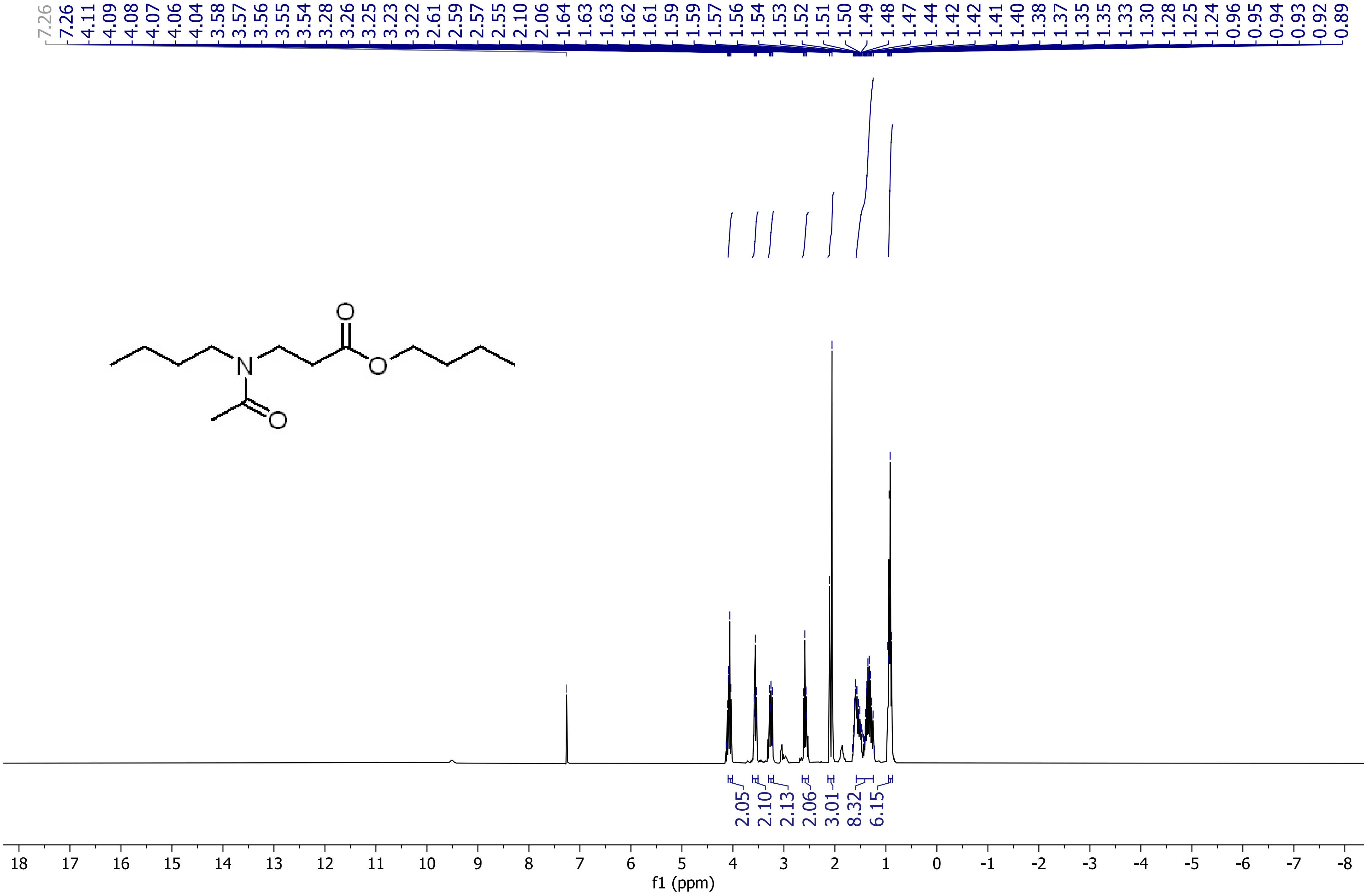

*Butyl 3-(N-butylacetamido)propanoate*

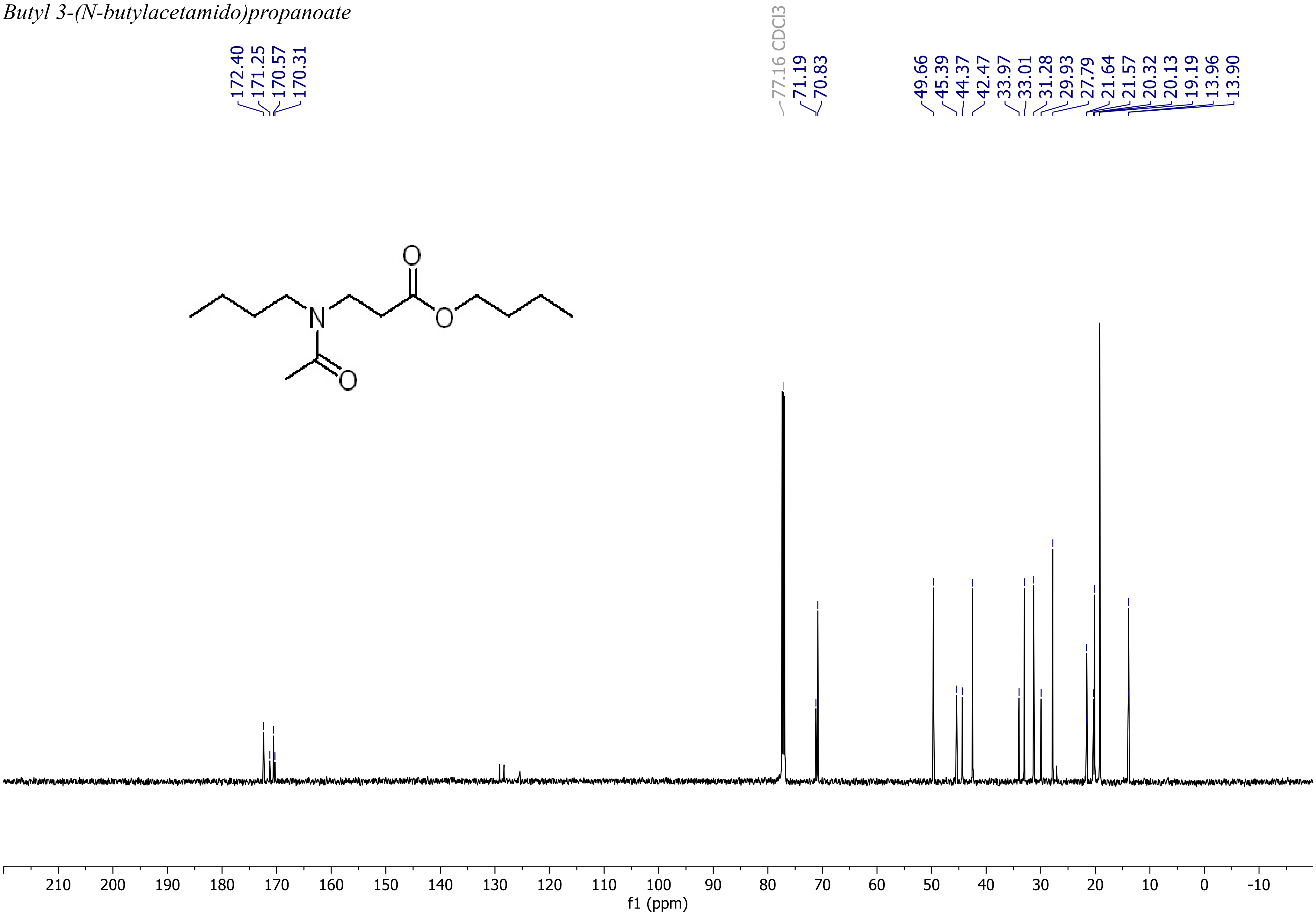

*amyl 3-(N-butylacetamido)propanoate*

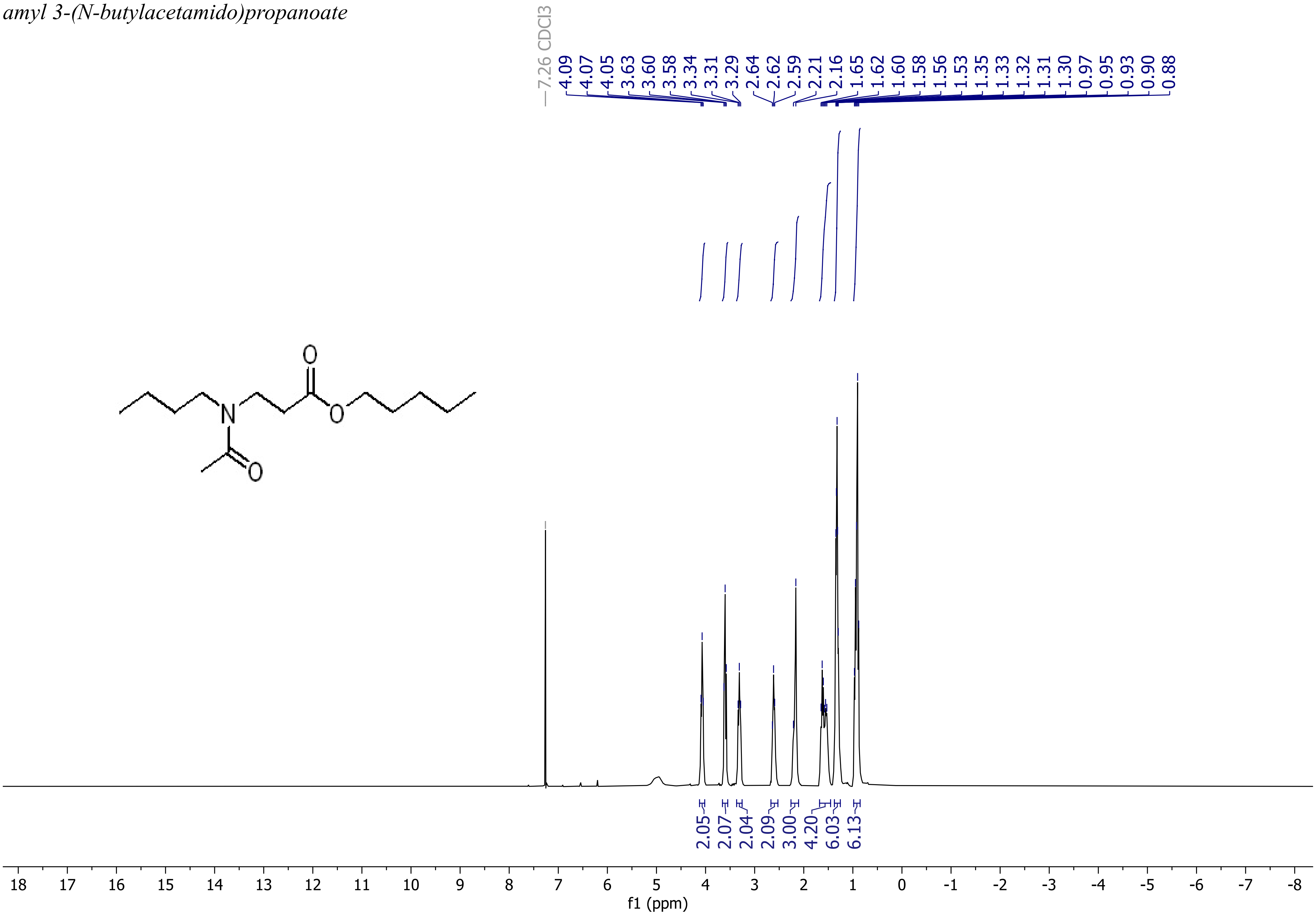

*amyl 3-(N-butylacetamido)propanoate*

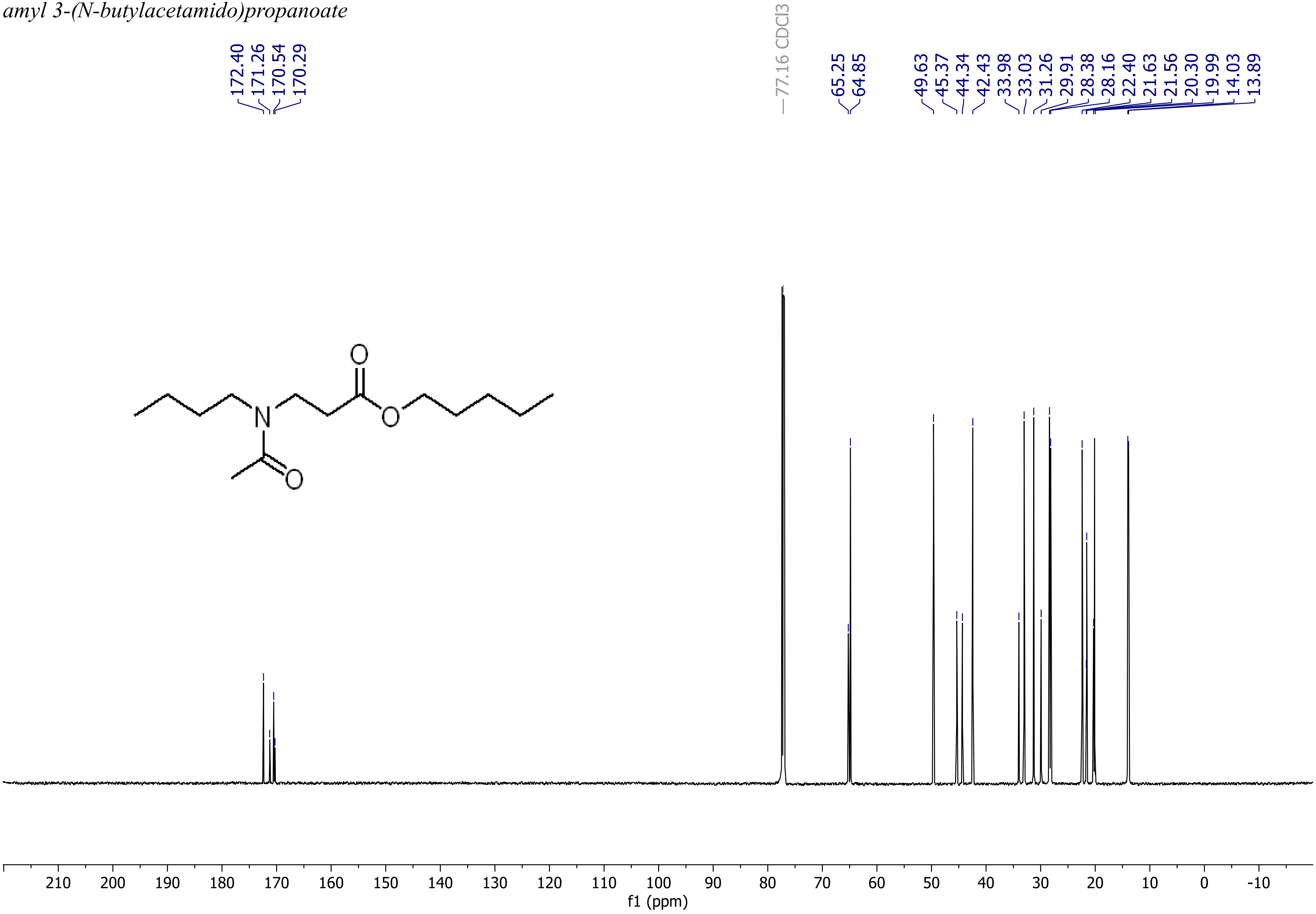

*Hexyl 3-(N-butylacetamido)propanoate*

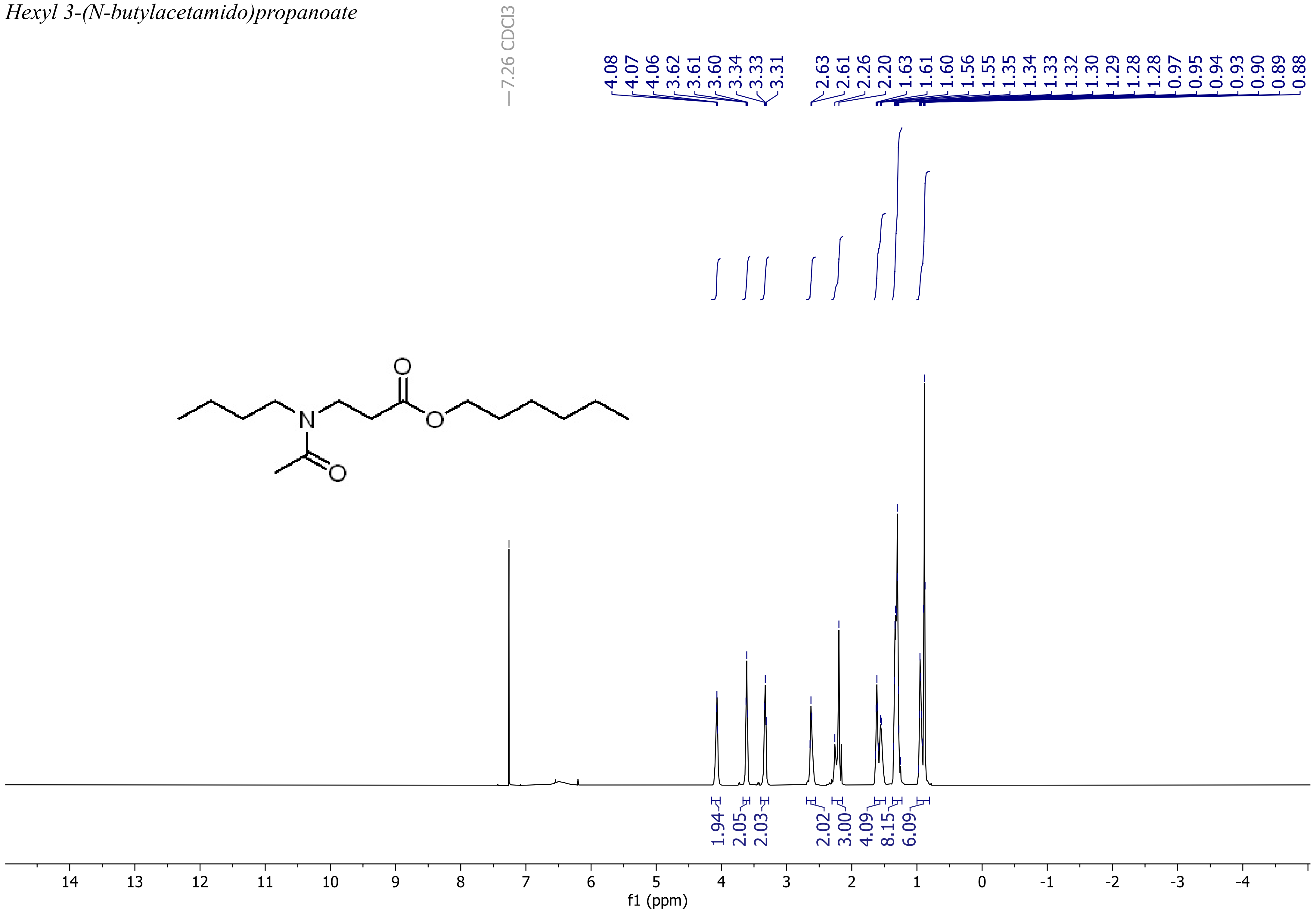

*Hexyl 3-(N-butylacetamido)propanoate*

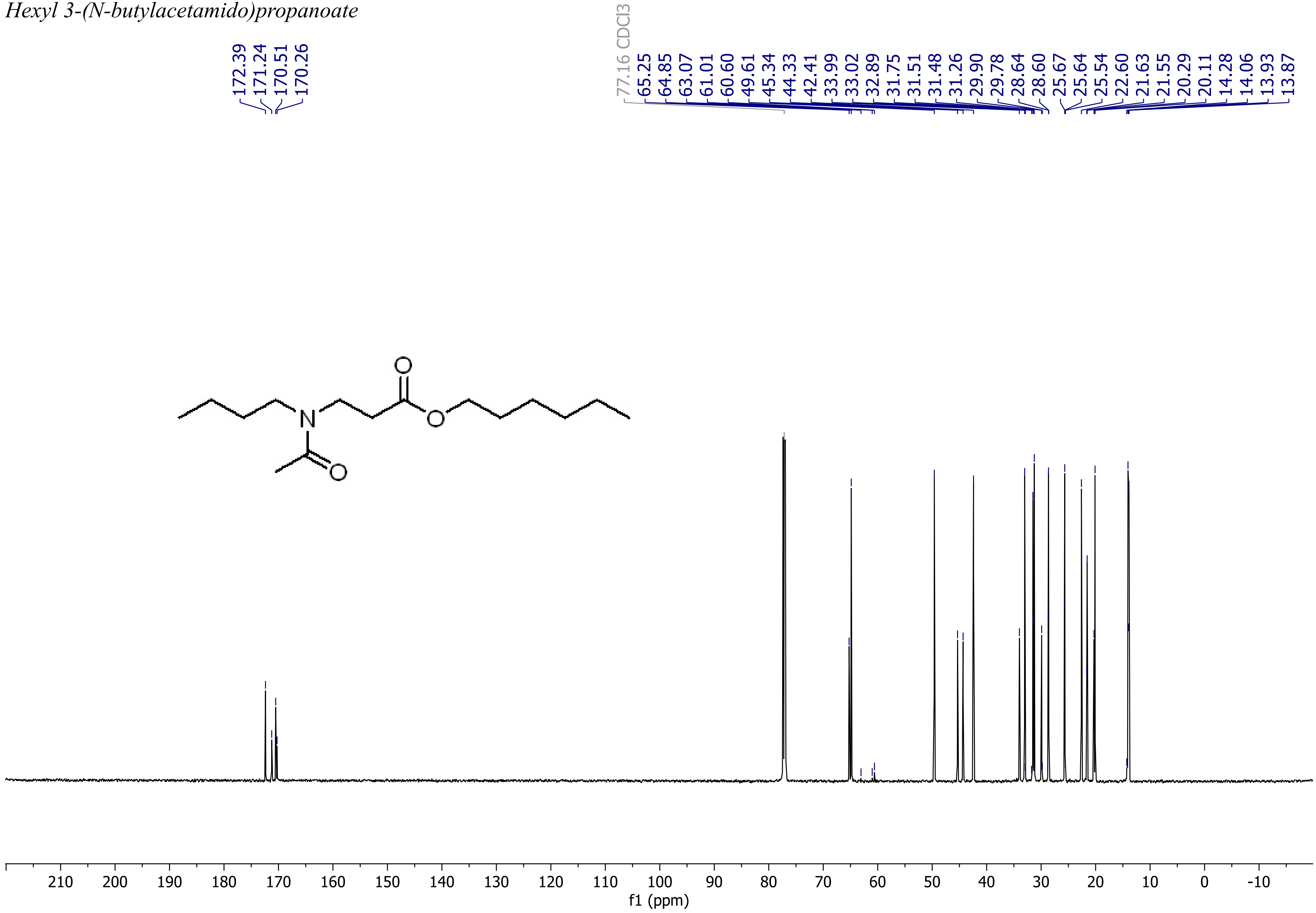

*Heptyl 3-(N-butylacetamido)propanoate*

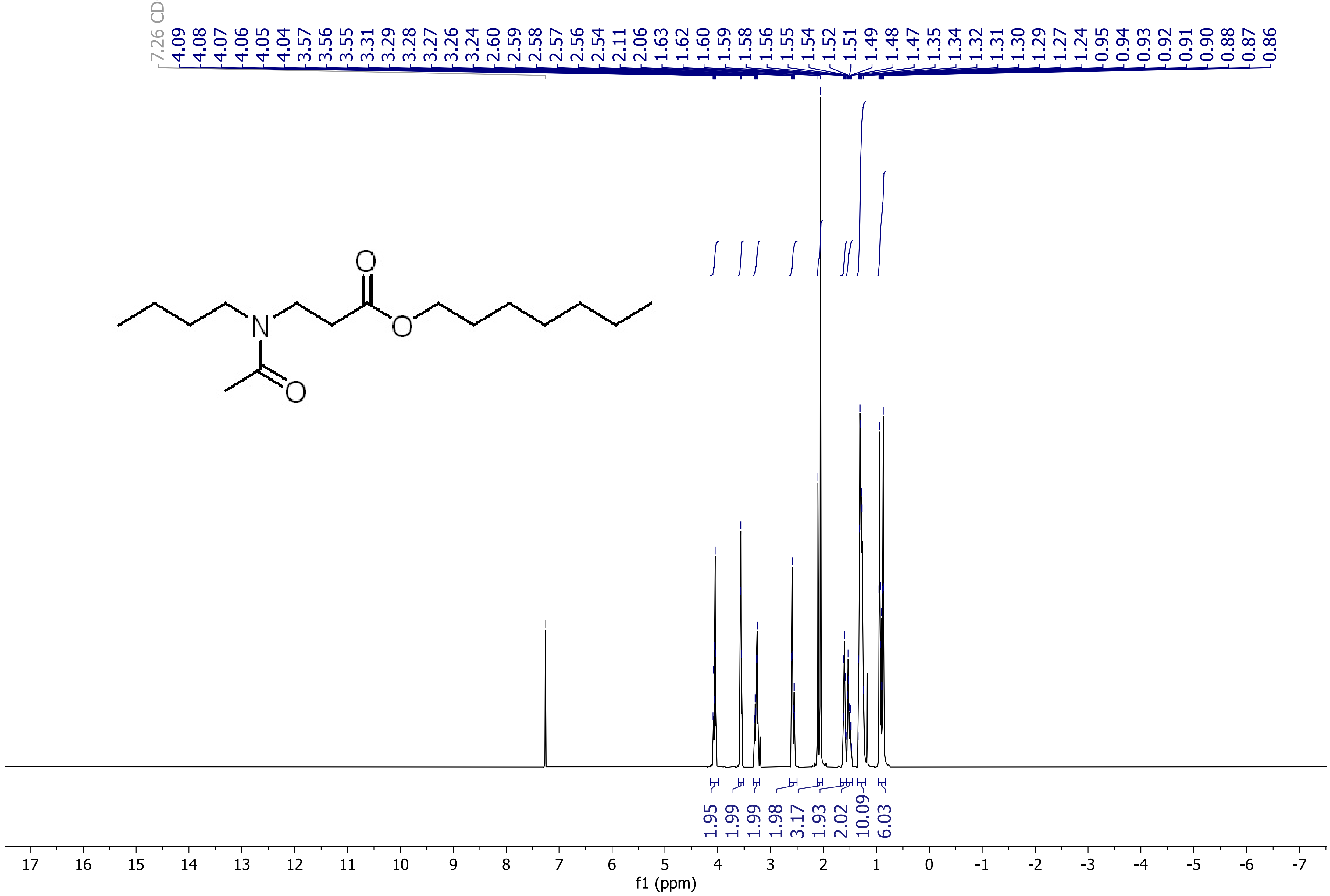

*Heptyl 3-(N-butylacetamido)propanoate*

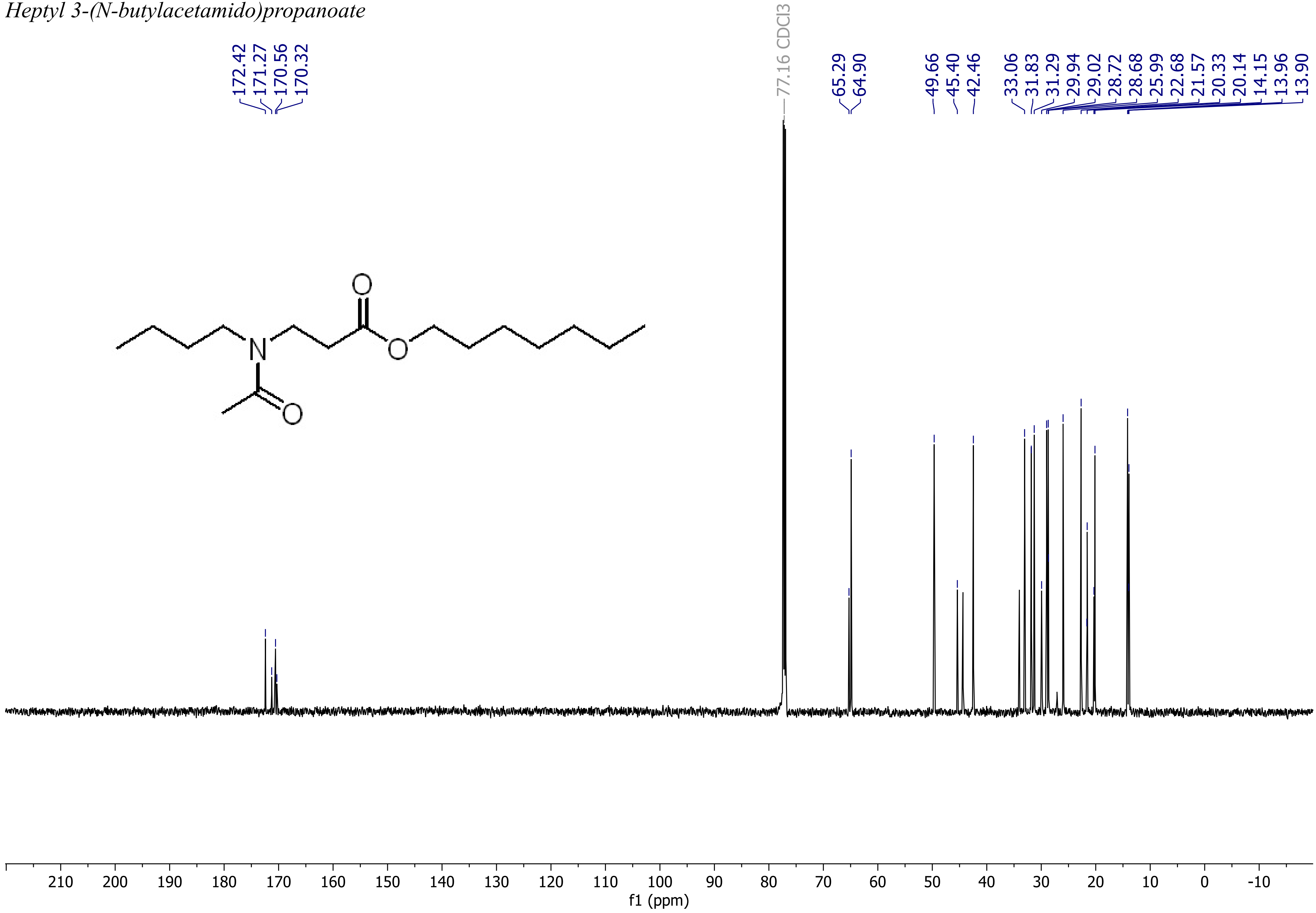

*Octyl 3-(N-butylacetamido)propanoate*

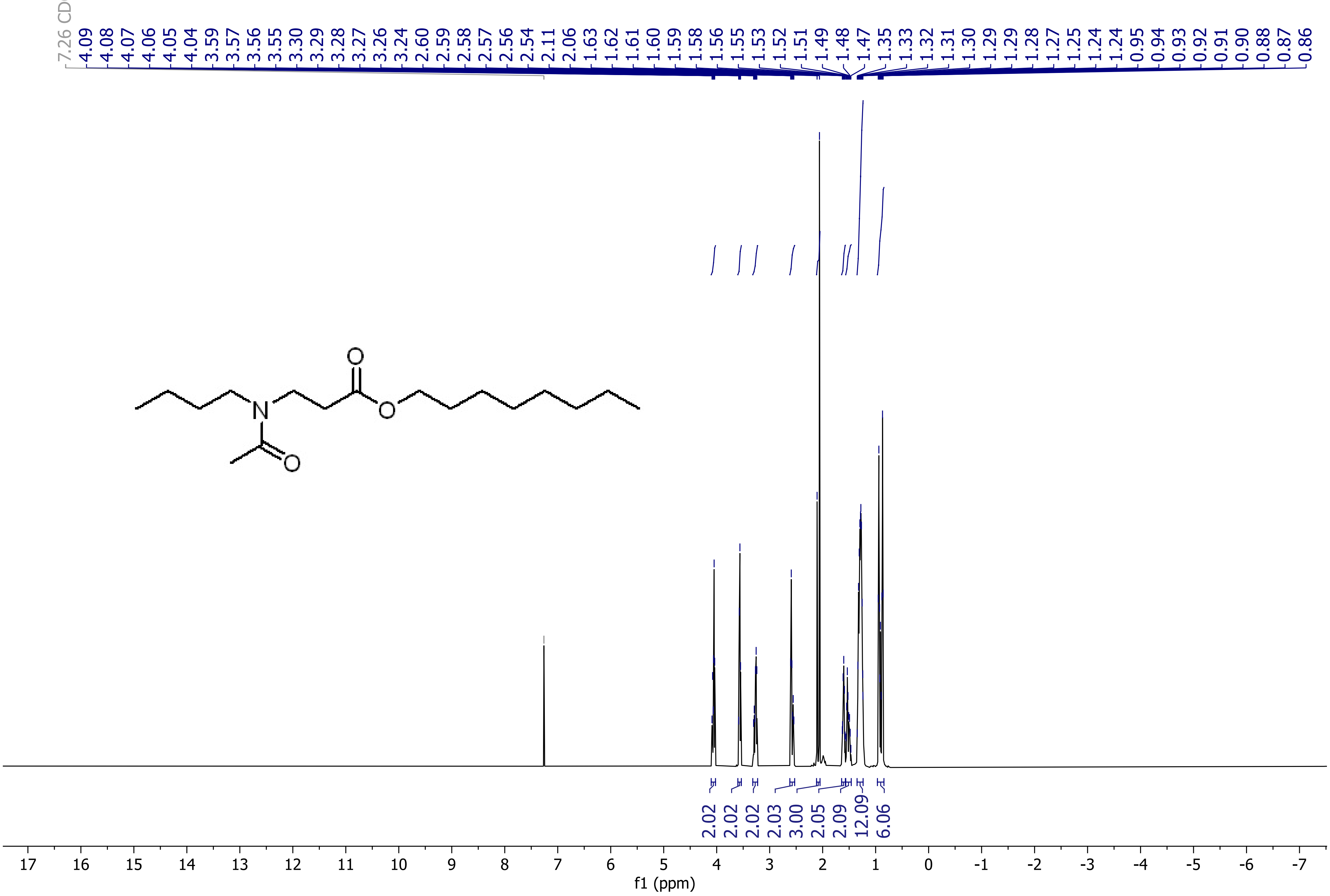

*Octyl 3-(N-butylacetamido)propanoate*

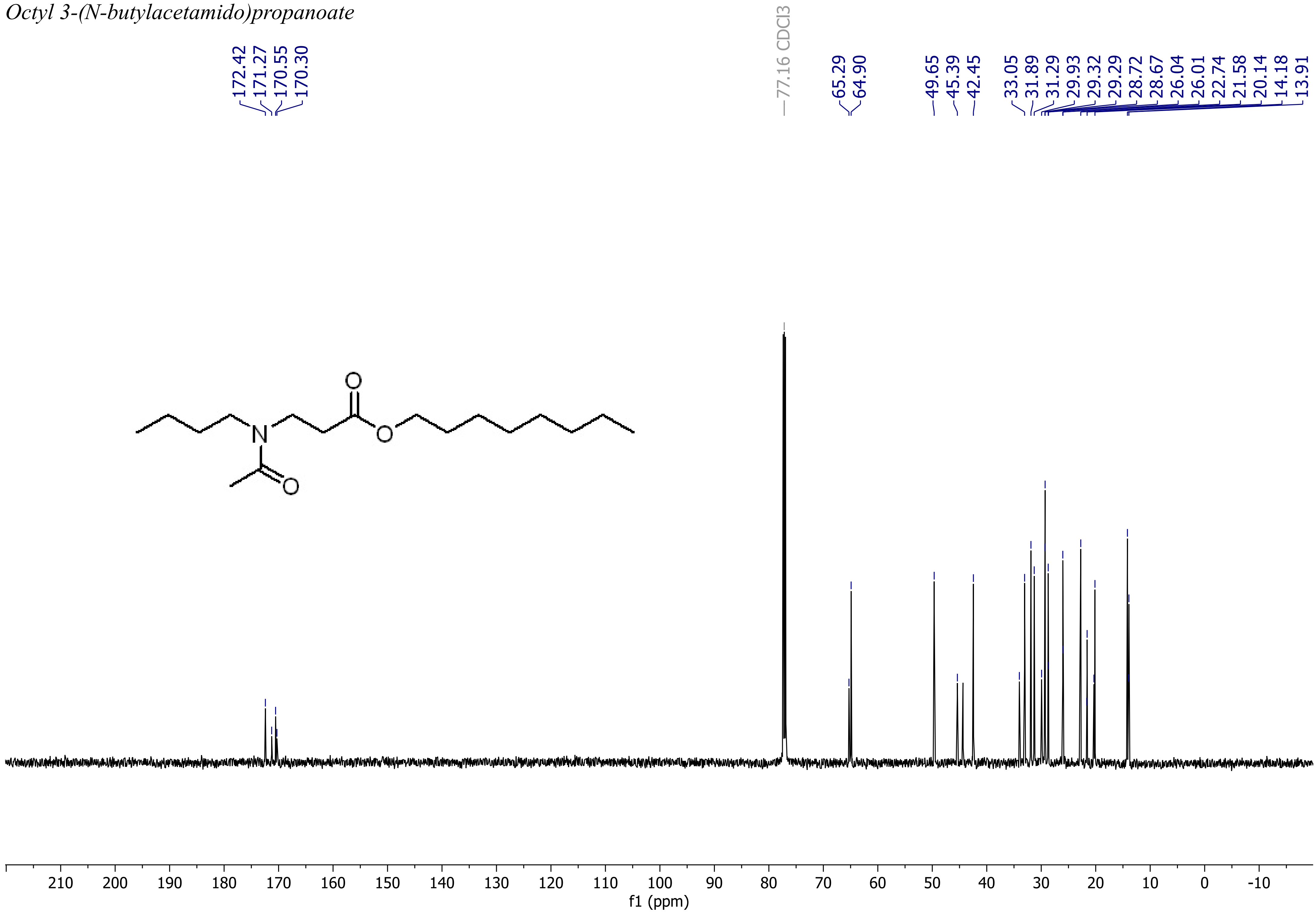

*Nonyl 3-(N-butylacetamido)propanoate*

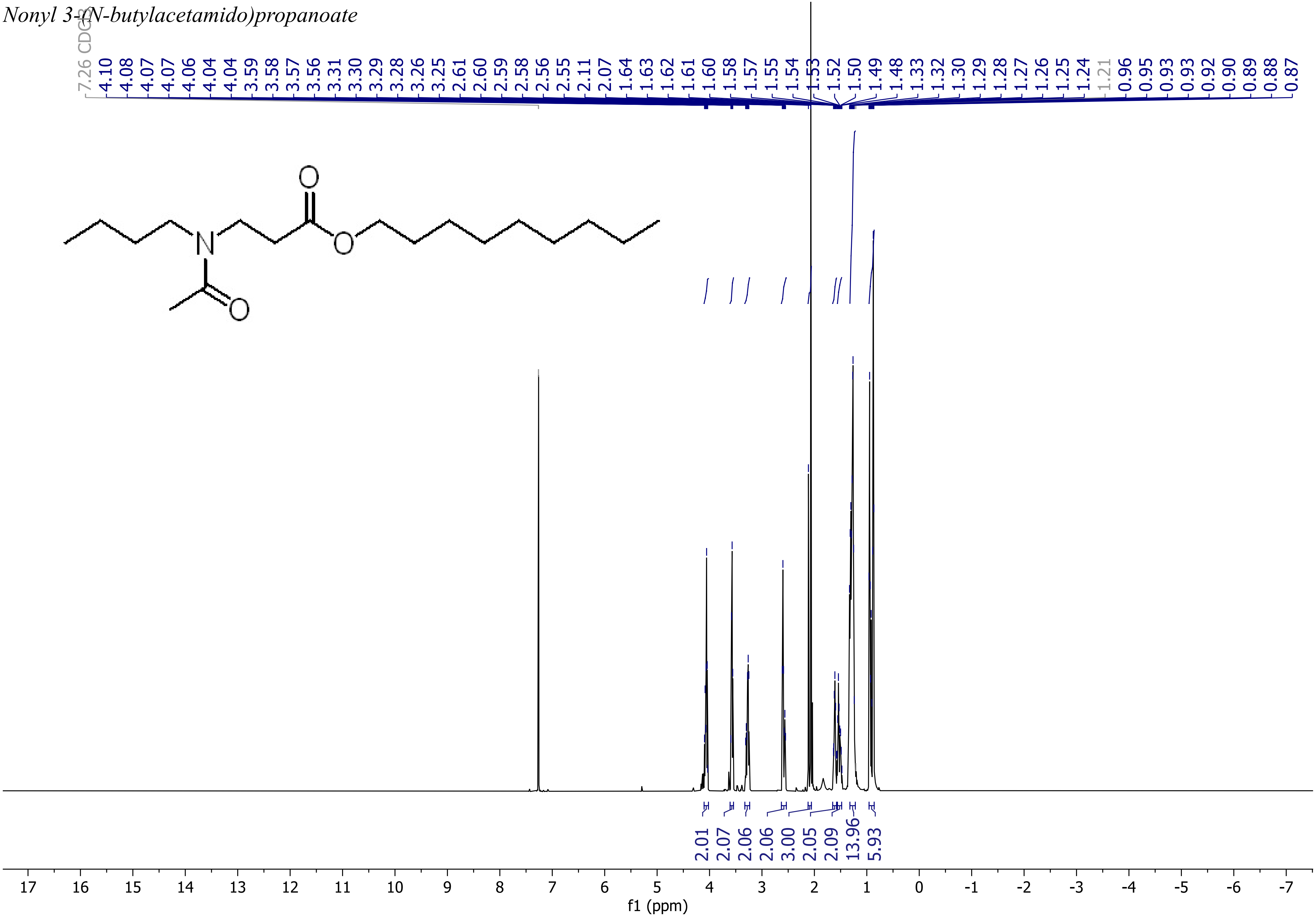

*Nonyl 3-(N-butylacetamido)propanoate*

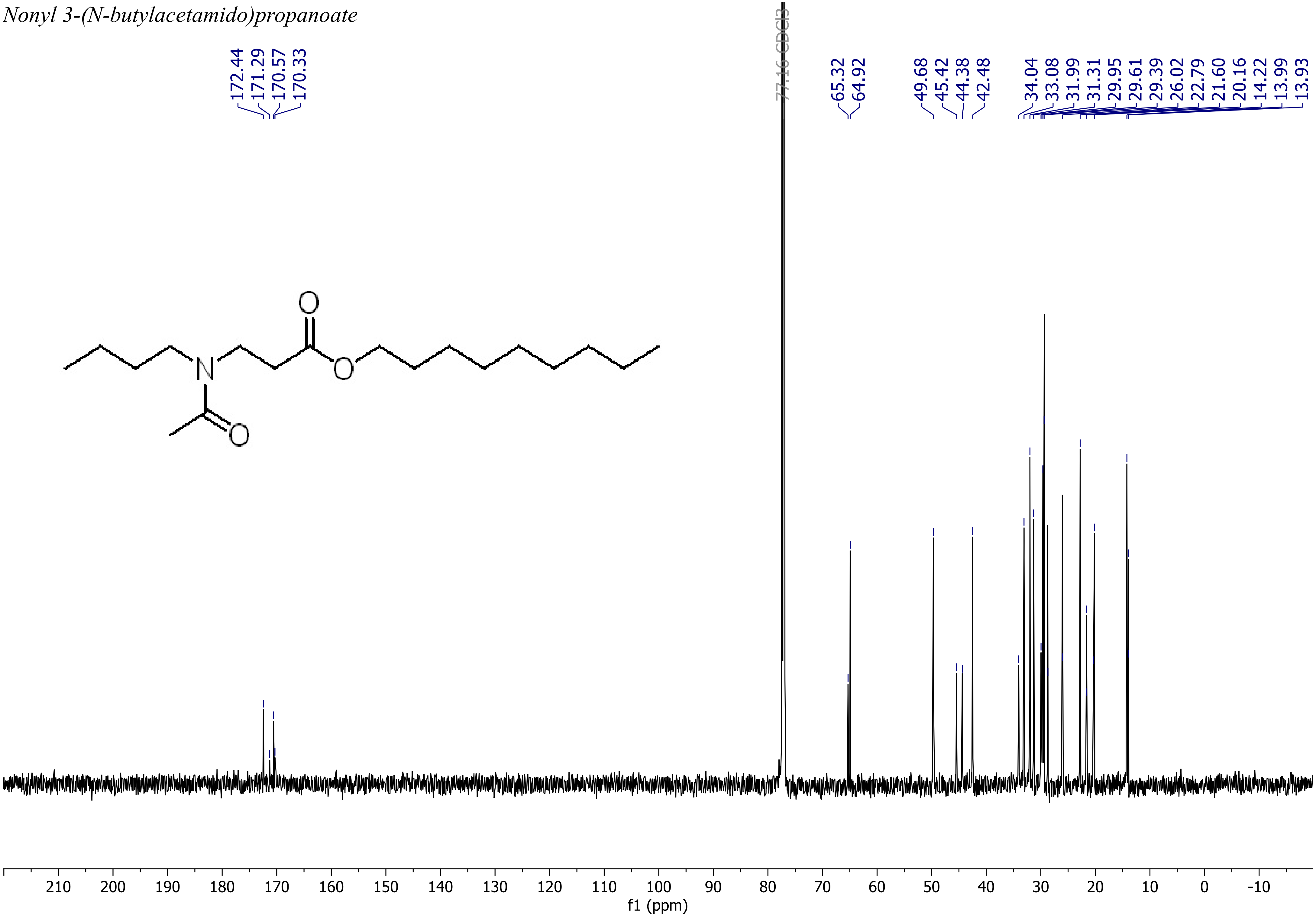

*Decyl 3-(N-butylacetamido)propanoate*

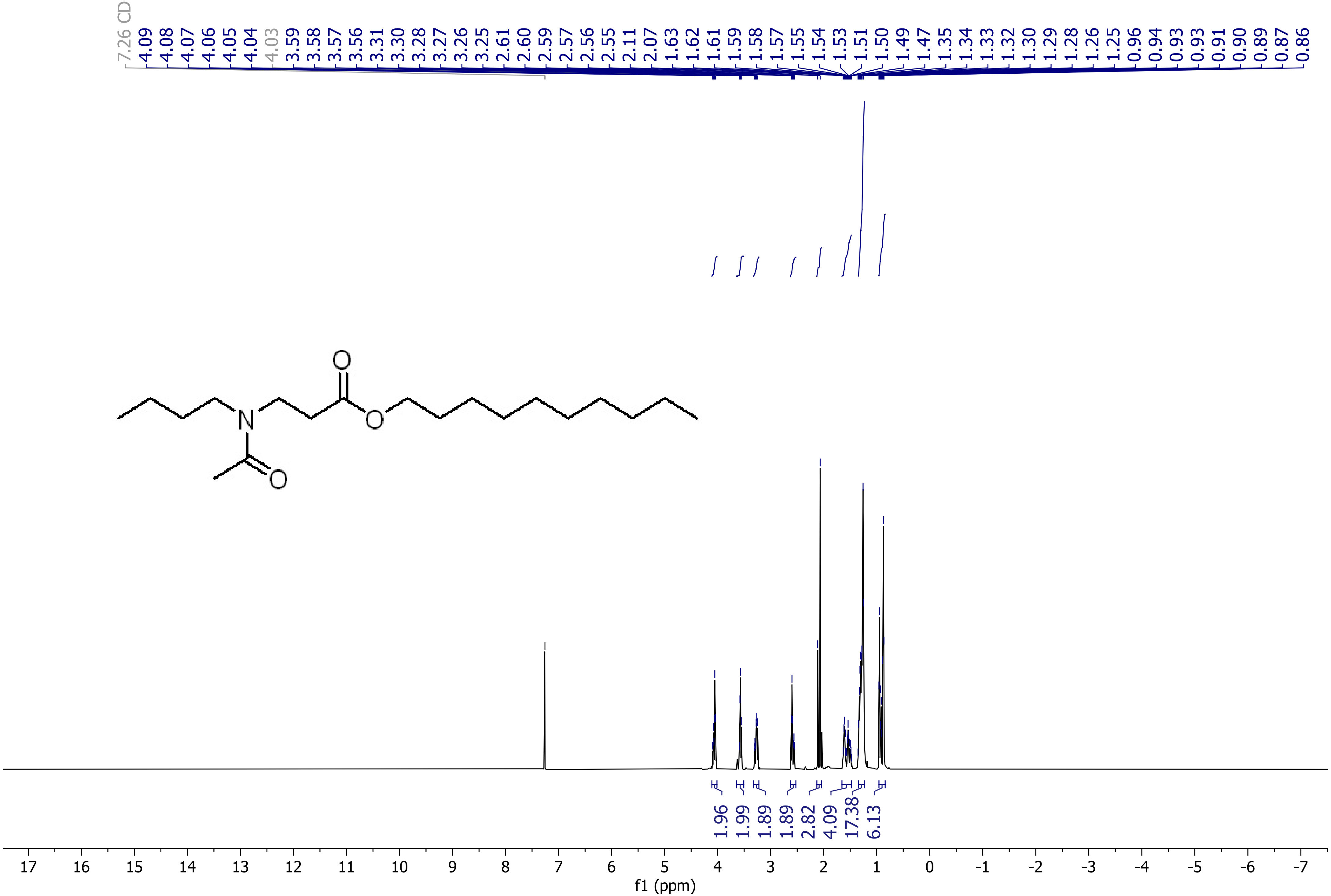

*Decyl 3-(N-butylacetamido)propanoate*

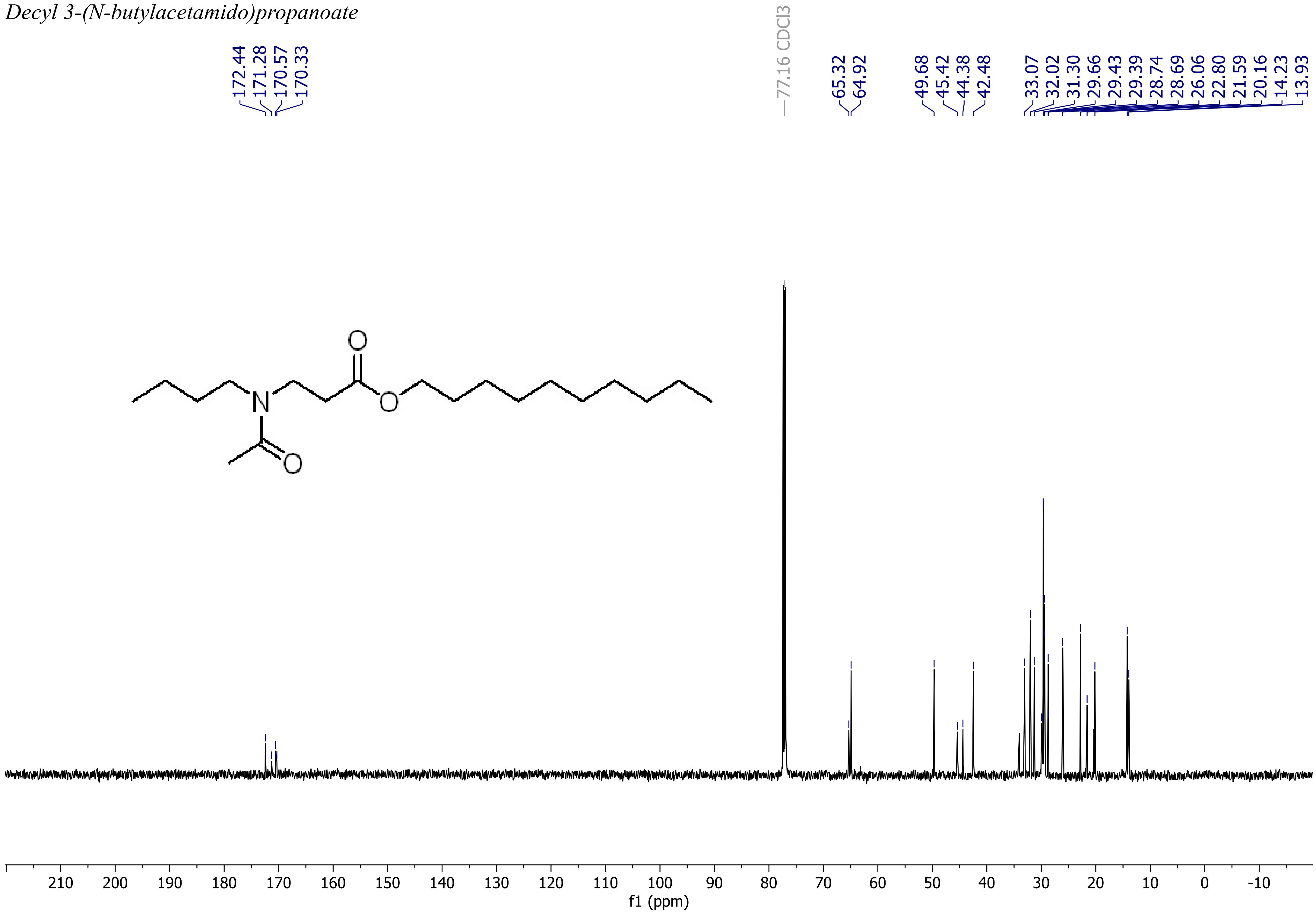

*Undecyl 3-(N-butylacetamido)propanoate*

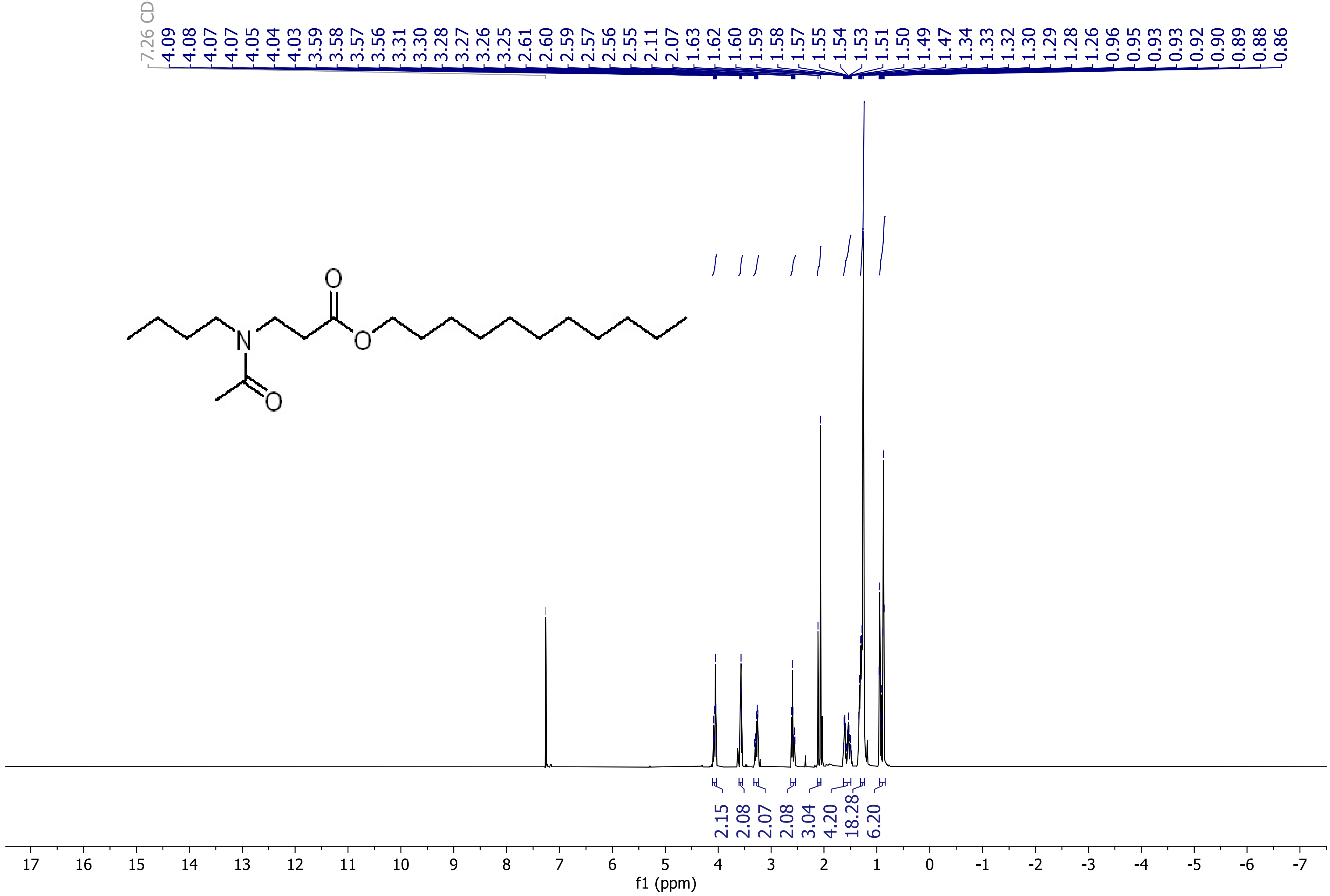

*Undecyl 3-(N-butylacetamido)propanoate*

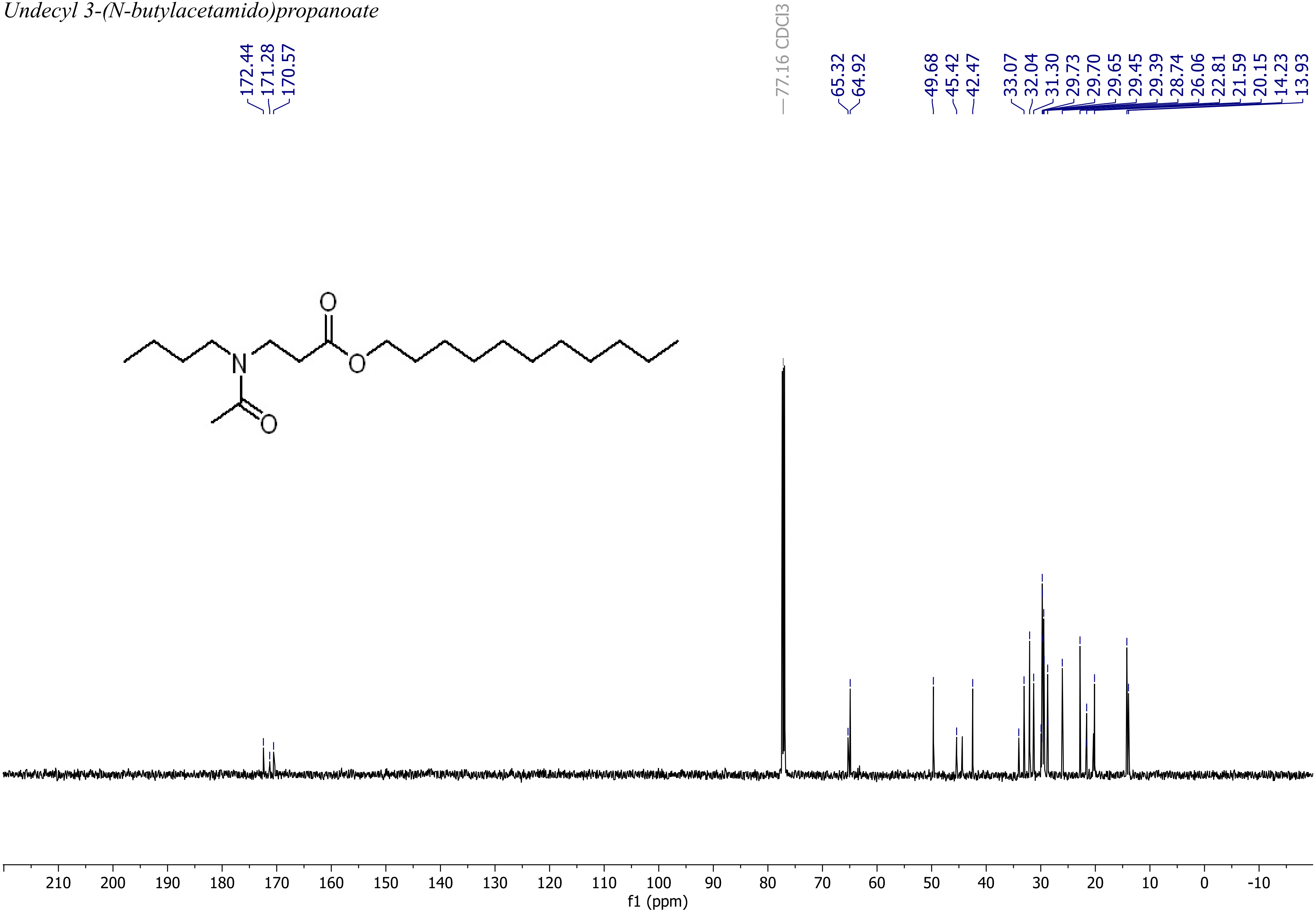

*Dodecyl 3-(N-butylacetamido)propanoate*

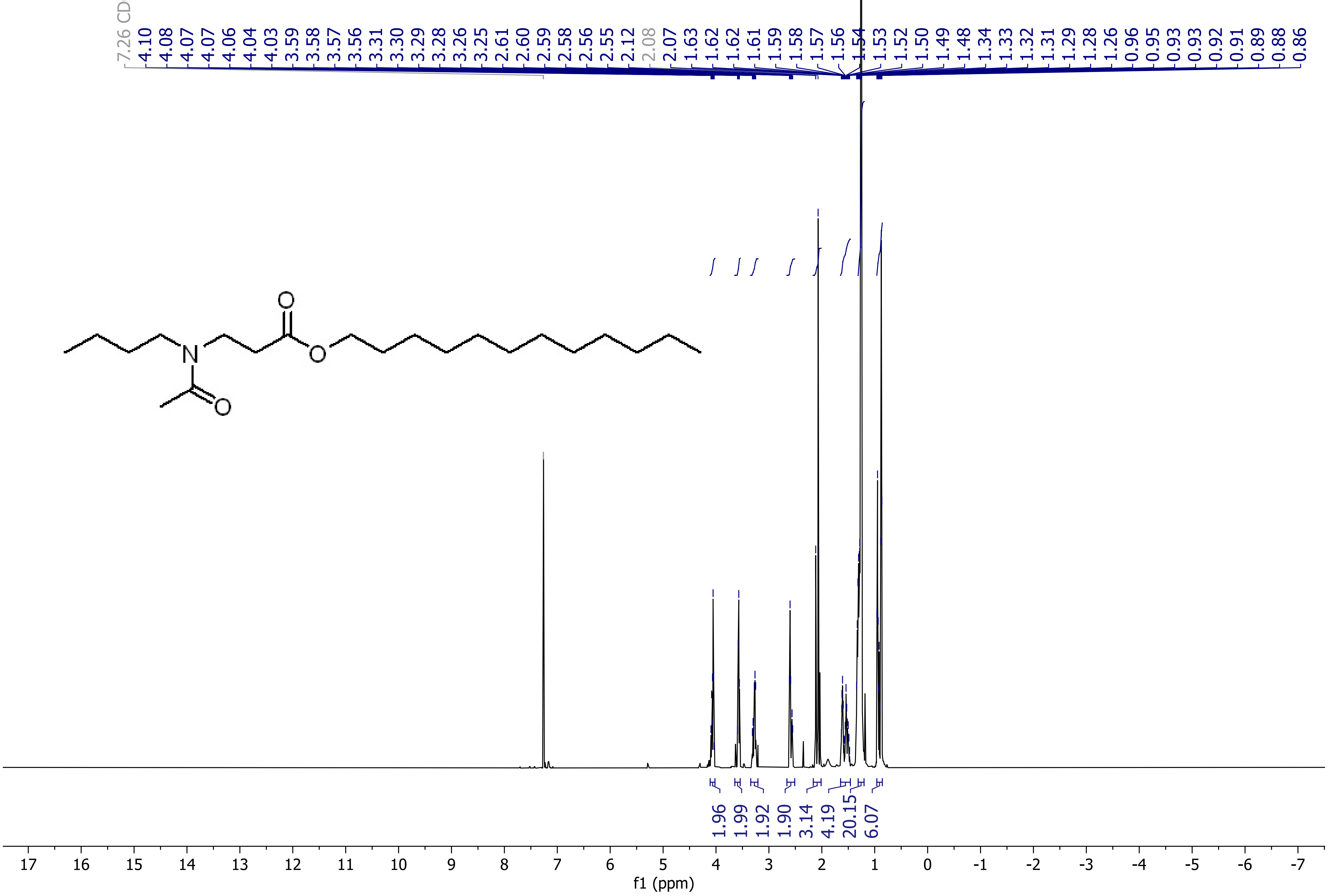

*Dodecyl 3-(N-butylacetamido)propanoate*

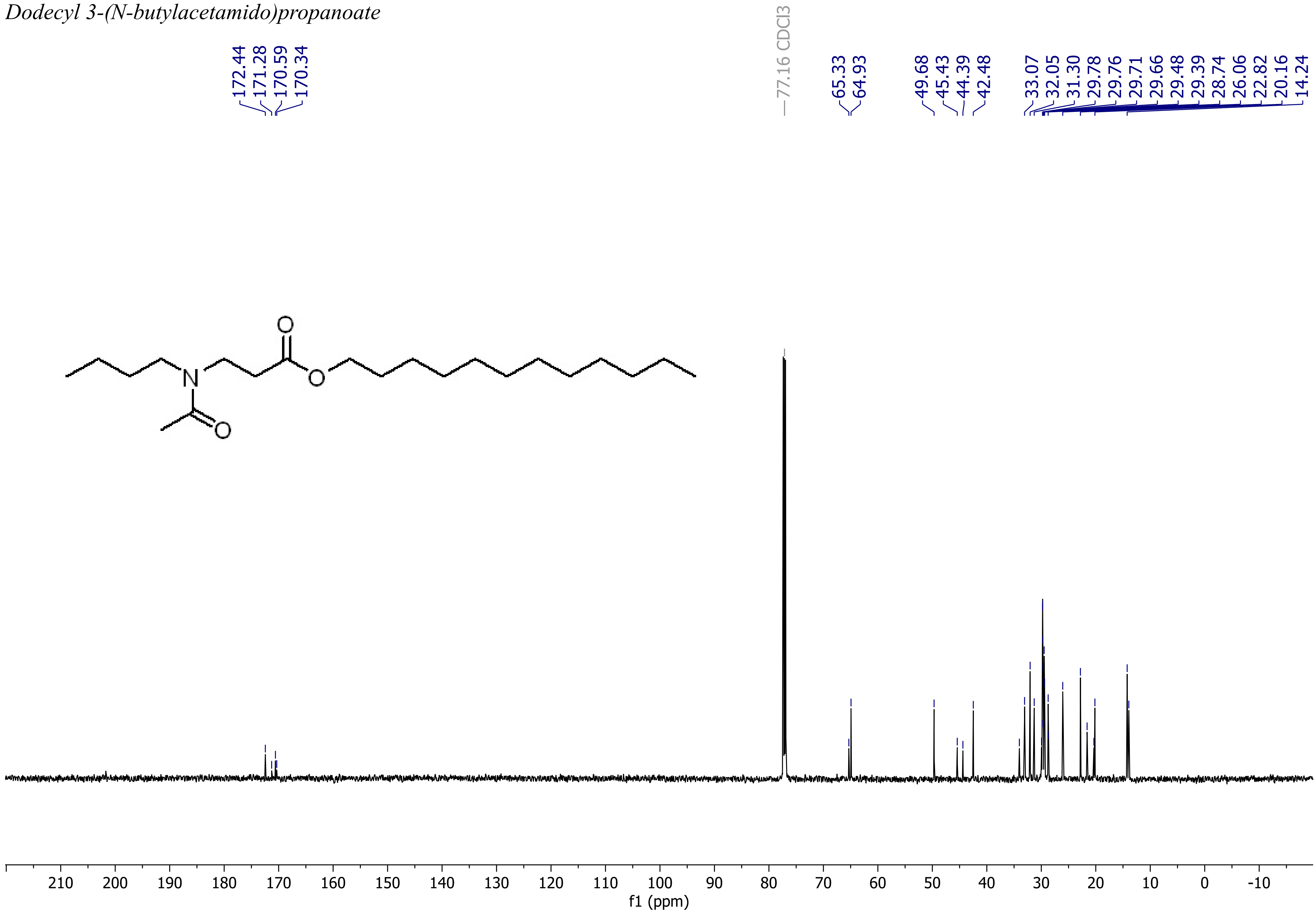

Supplement: Supplementary file 1 [file molecules-31-03570-s001.zip › Supplementary FigureS2_NMRSpectra.pdf]
