# Supplementary material for: Synthesis, Characterization and Biological Activity of Alkyl 3-(N-Butylacetamido)propanoates Against Ixodid Ticks
Source: Molecules. 2026 Oct 8;31(19):3570. doi: 10.3390/molecules31193570 (PMC13666575; doi:10.3390/molecules31193570)
Supplement: Supplementary file 1 [file molecules-31-03570-s001.zip › Supplementary FigureS3_MSspectra.pdf]

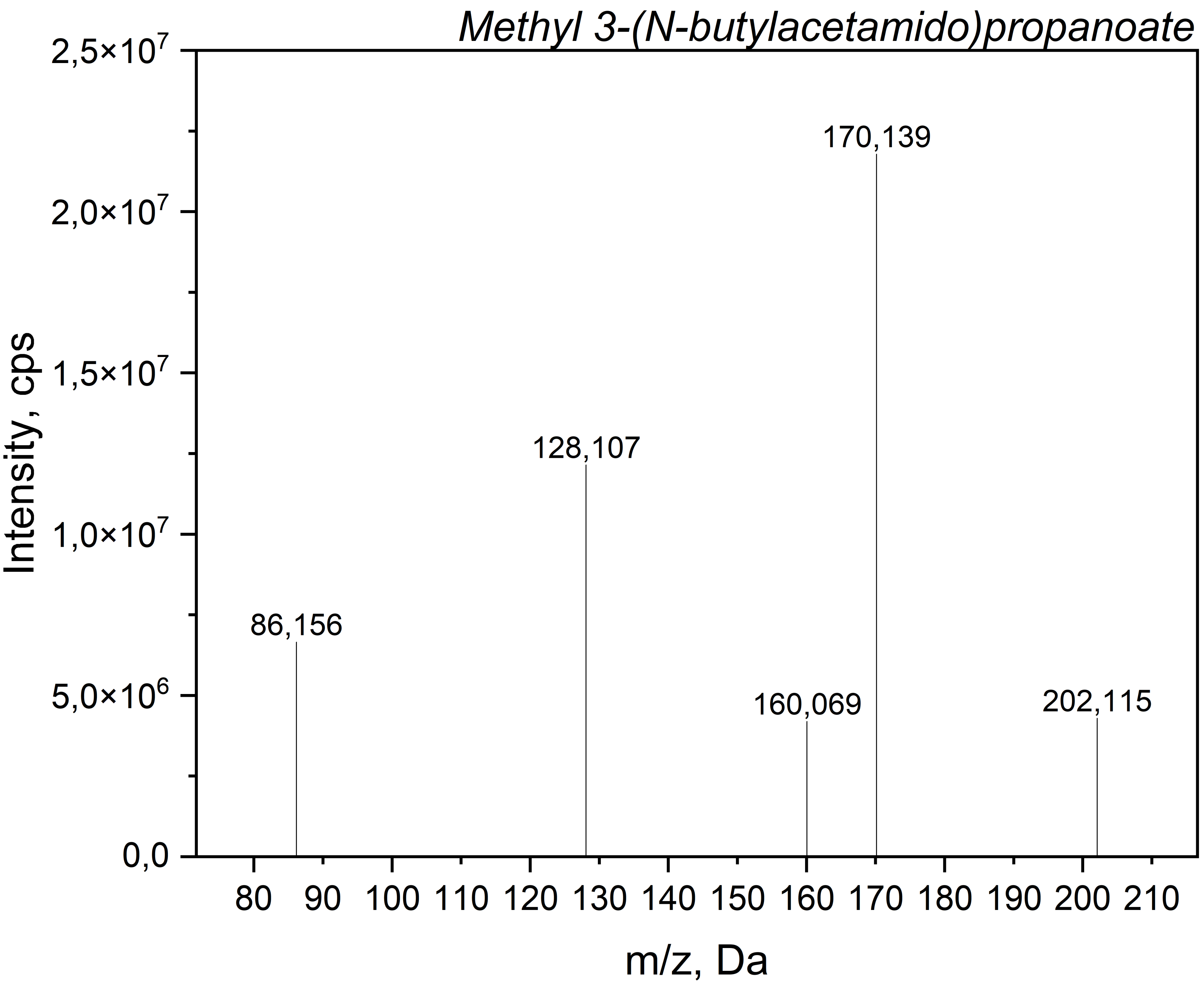
Methyl 3-(N-butylacetamido)propanoate
Intensity, cps
m/z, Da
2,5×10^7
2,0×10^7
1,5×10^7
1,0×10^7
5,0×10^6
0,0
80 90 100 110 120 130 140 150 160 170 180 190 200 210
86,156
128,107
160,069
170,139
202,115

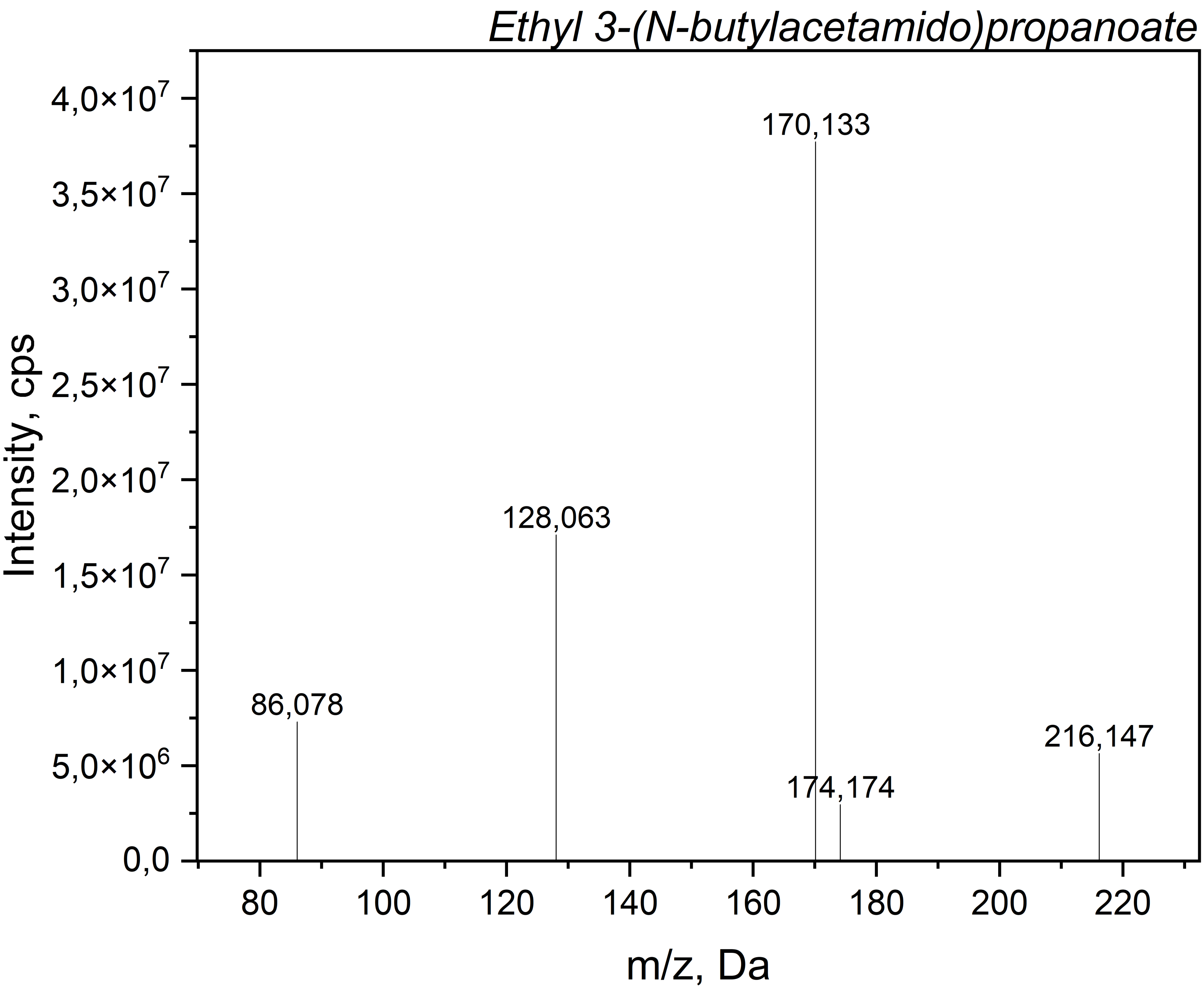
Ethyl 3-(N-butylacetamido)propanoate
Intensity, cps
m/z, Da
4,0×10^7
3,5×10^7
3,0×10^7
2,5×10^7
2,0×10^7
1,5×10^7
1,0×10^7
5,0×10^6
0,0
80
100
120
140
160
180
200
220
170,133
128,063
86,078
216,147
174,174

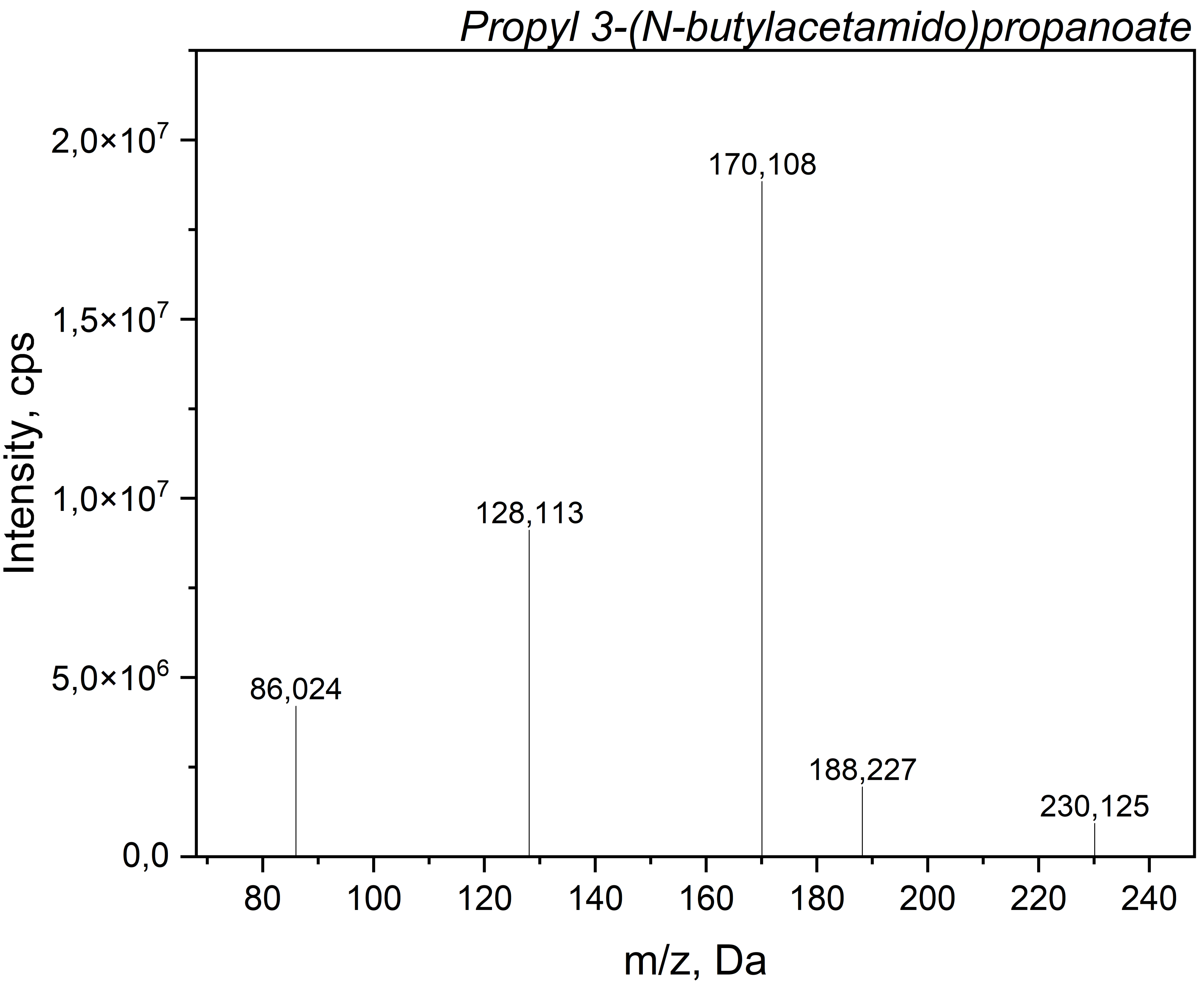
Propyl 3-(N-butylacetamido)propanoate
Intensity, cps
m/z, Da
170,108
128,113
86,024
188,227
230,125
2,0×10^7
1,5×10^7
1,0×10^7
5,0×10^6
0,0
80
100
120
140
160
180
200
220
240

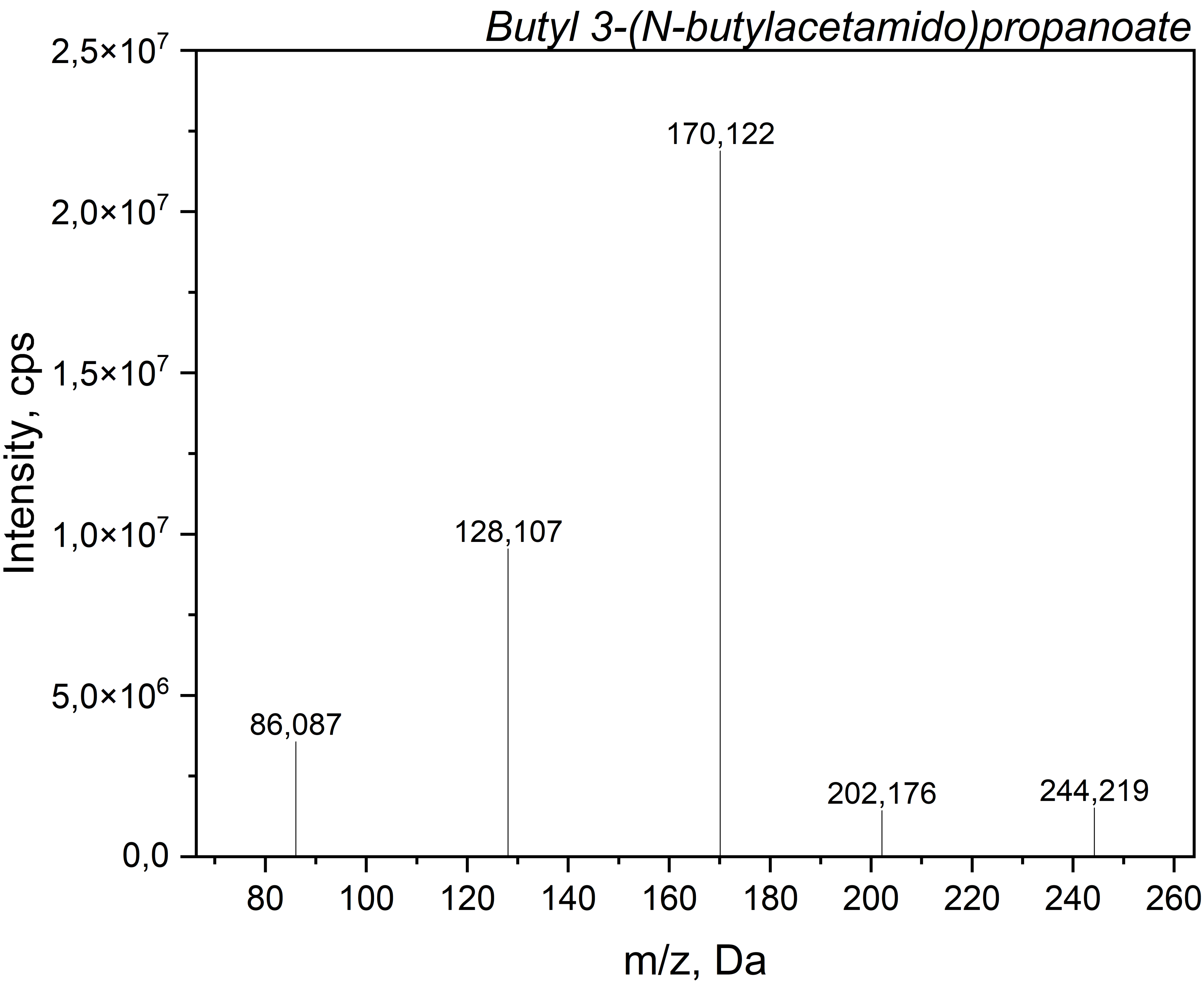
Butyl 3-(N-butylacetamido)propanoate
2,5×10^7
2,0×10^7
1,5×10^7
1,0×10^7
5,0×10^6
0,0
Intensity, cps
170,122
128,107
86,087
202,176
244,219
80
100
120
140
160
180
200
220
240
260
m/z, Da

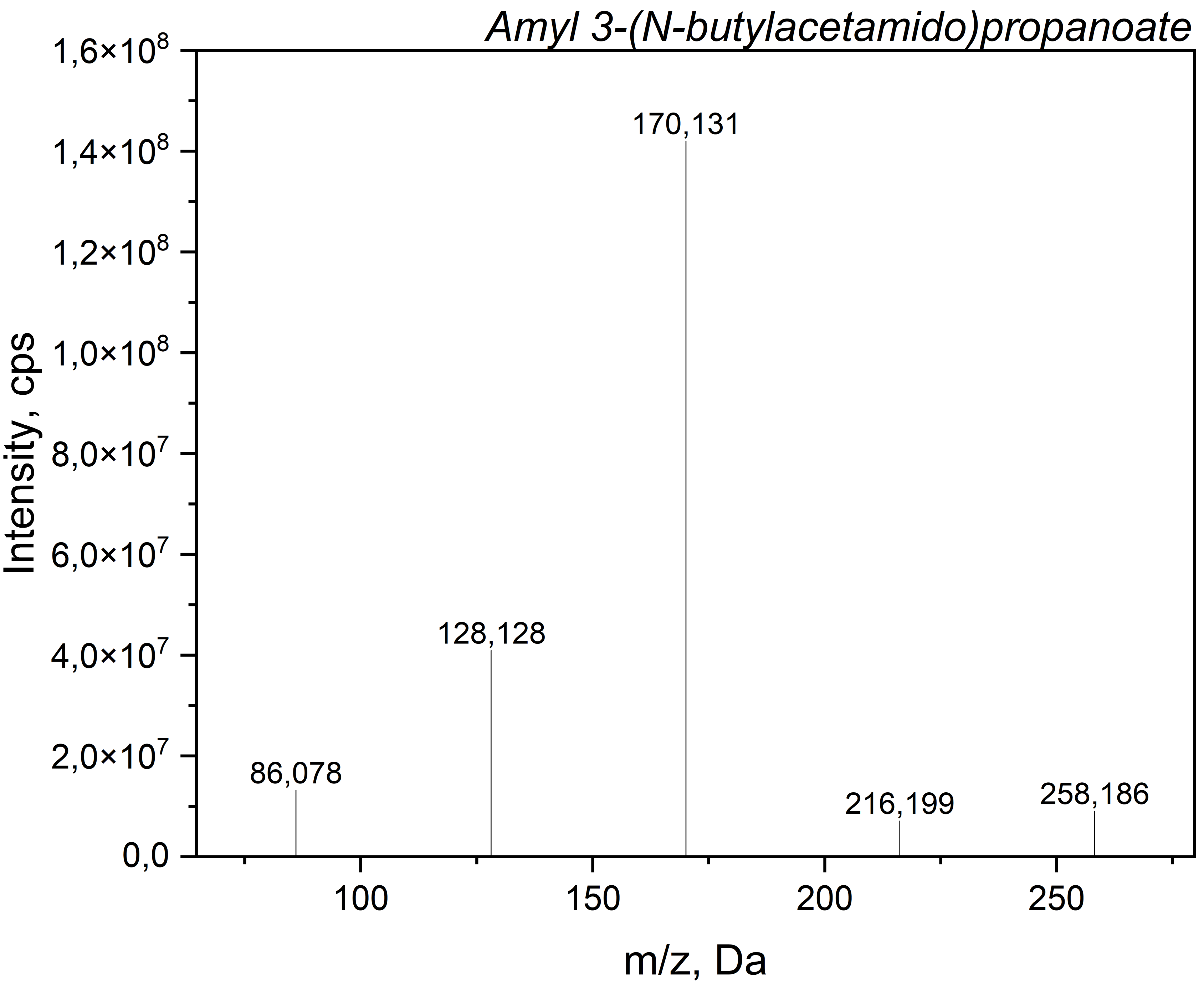
Amyl 3-(N-butylacetamido)propanoate
1,6×10^8
1,4×10^8
1,2×10^8
1,0×10^8
8,0×10^7
6,0×10^7
4,0×10^7
2,0×10^7
0,0
Intensity, cps
170,131
128,128
86,078
216,199
258,186
100
150
200
250
m/z, Da

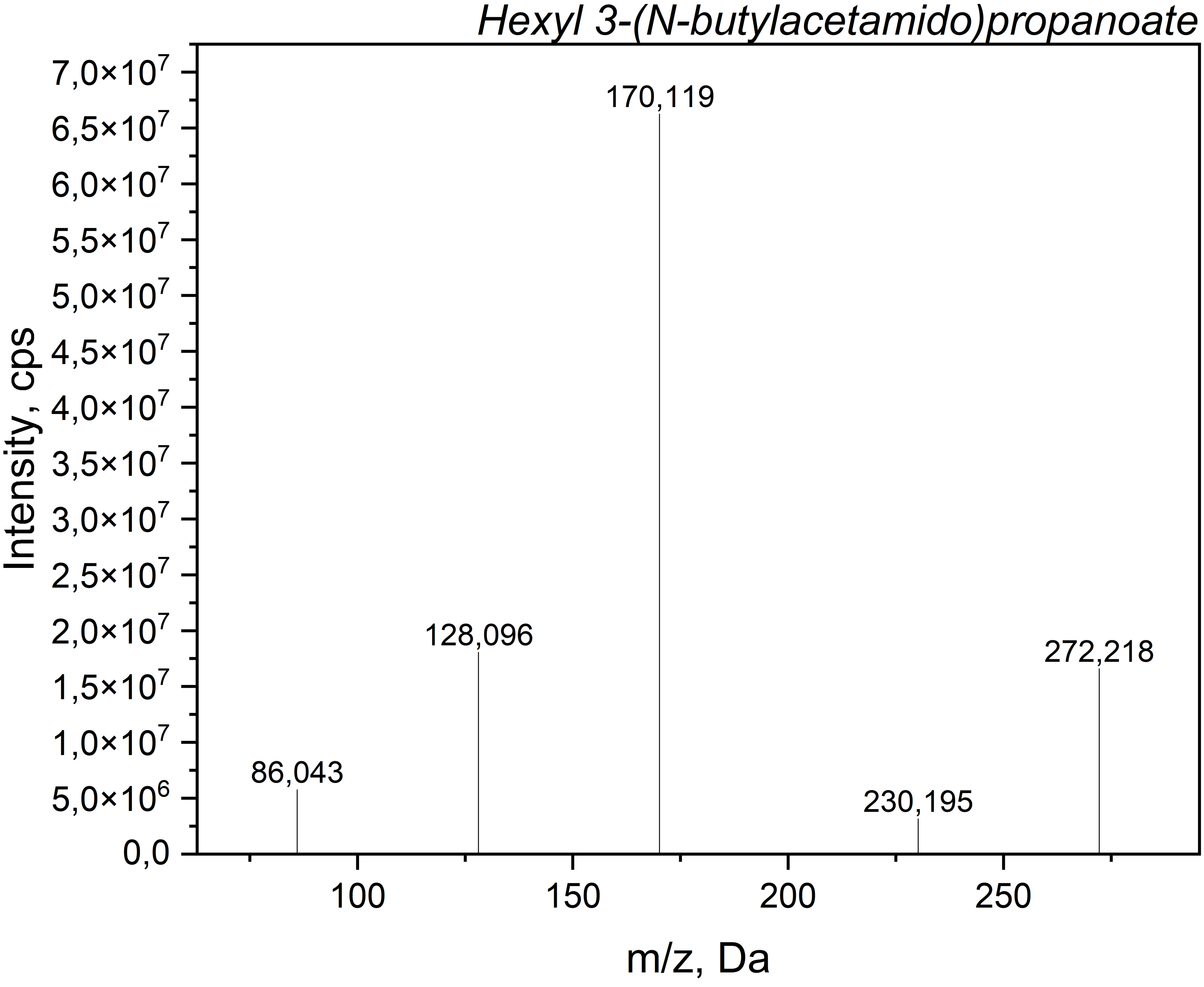

Hexyl 3-(N-butylacetamido)propanoate
Intensity, cps
m/z, Da
170,119
128,096
272,218
86,043
230,195
7,0×10^7
6,5×10^7
6,0×10^7
5,5×10^7
5,0×10^7
4,5×10^7
4,0×10^7
3,5×10^7
3,0×10^7
2,5×10^7
2,0×10^7
1,5×10^7
1,0×10^7
5,0×10^6
0,0
100
150
200
250

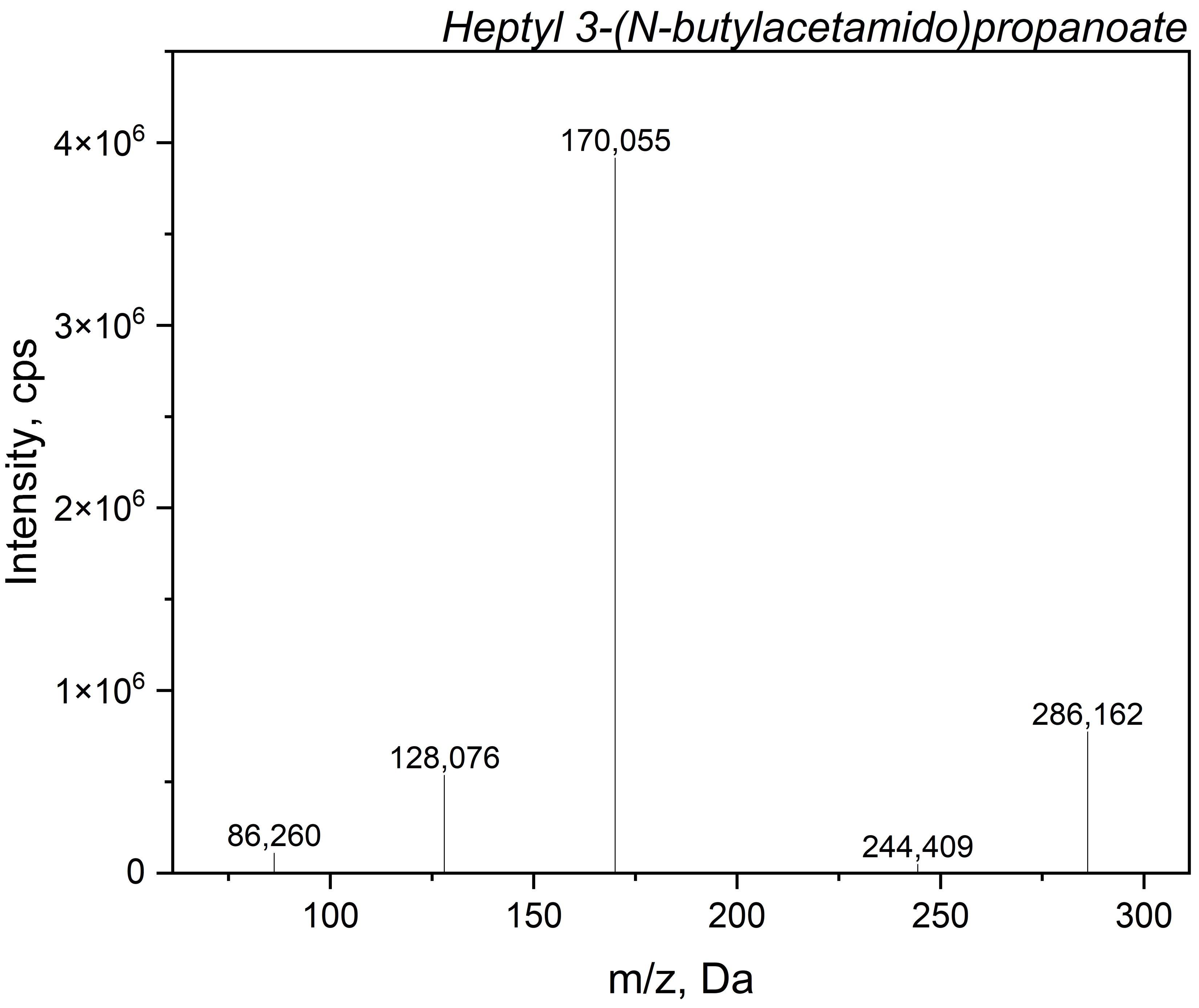
Heptyl 3-(N-butylacetamido)propanoate
Intensity, cps
m/z, Da
4×10^6
3×10^6
2×10^6
1×10^6
0
100
150
200
250
300
170,055
128,076
86,260
244,409
286,162

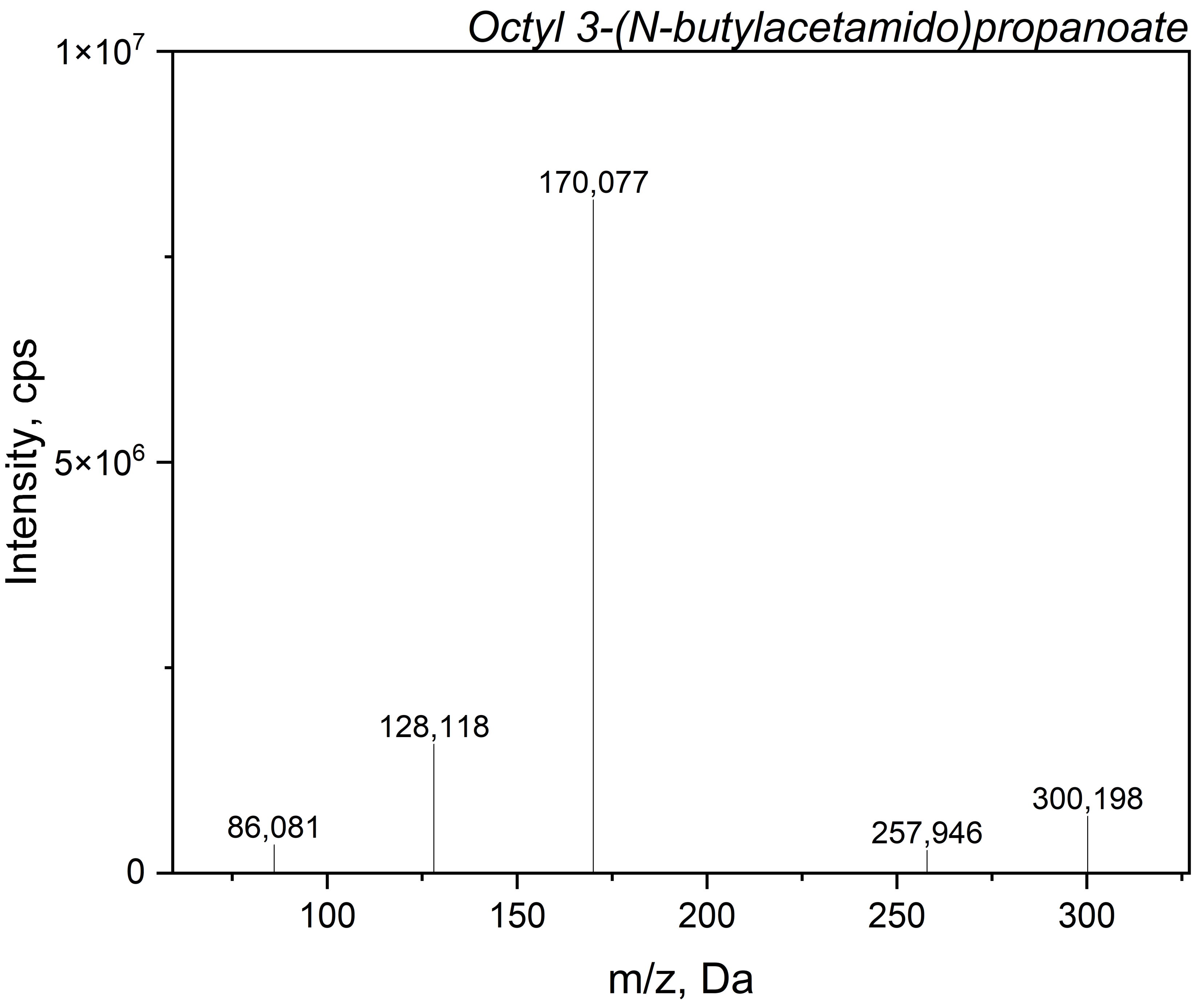
Octyl 3-(N-butylacetamido)propanoate
1×10^7
5×10^6
0
Intensity, cps
170,077
128,118
86,081
257,946
300,198
100
150
200
250
300
m/z, Da

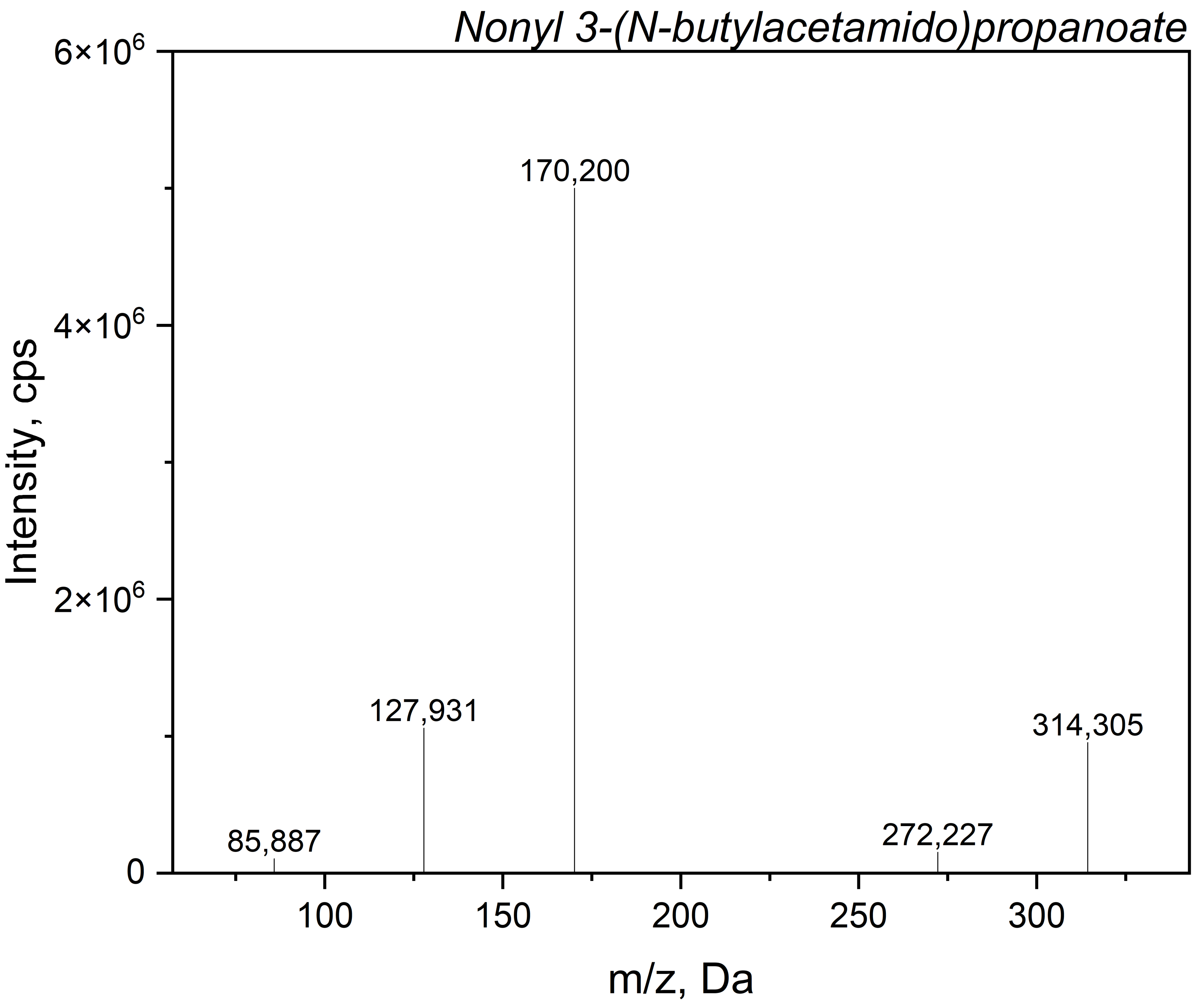

Nonyl 3-(N-butylacetamido)propanoate
6×10^6
4×10^6
2×10^6
0
Intensity, cps
170,200
127,931
314,305
85,887
272,227
100
150
200
250
300
m/z, Da

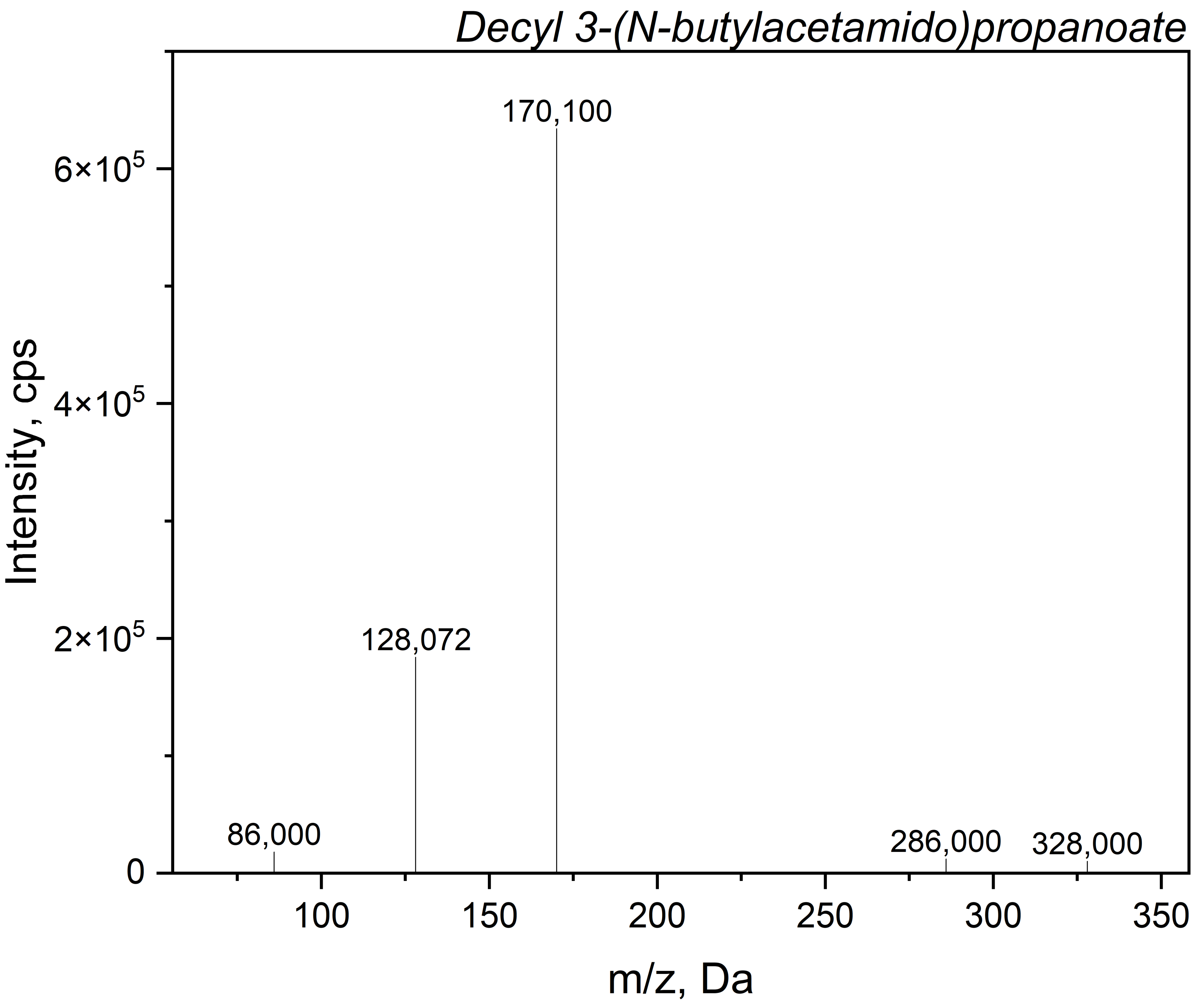

Decyl 3-(N-butylacetamido)propanoate
170,100
128,072
86,000
286,000
328,000
6×10^5
4×10^5
2×10^5
0
Intensity, cps
100
150
200
250
300
350
m/z, Da

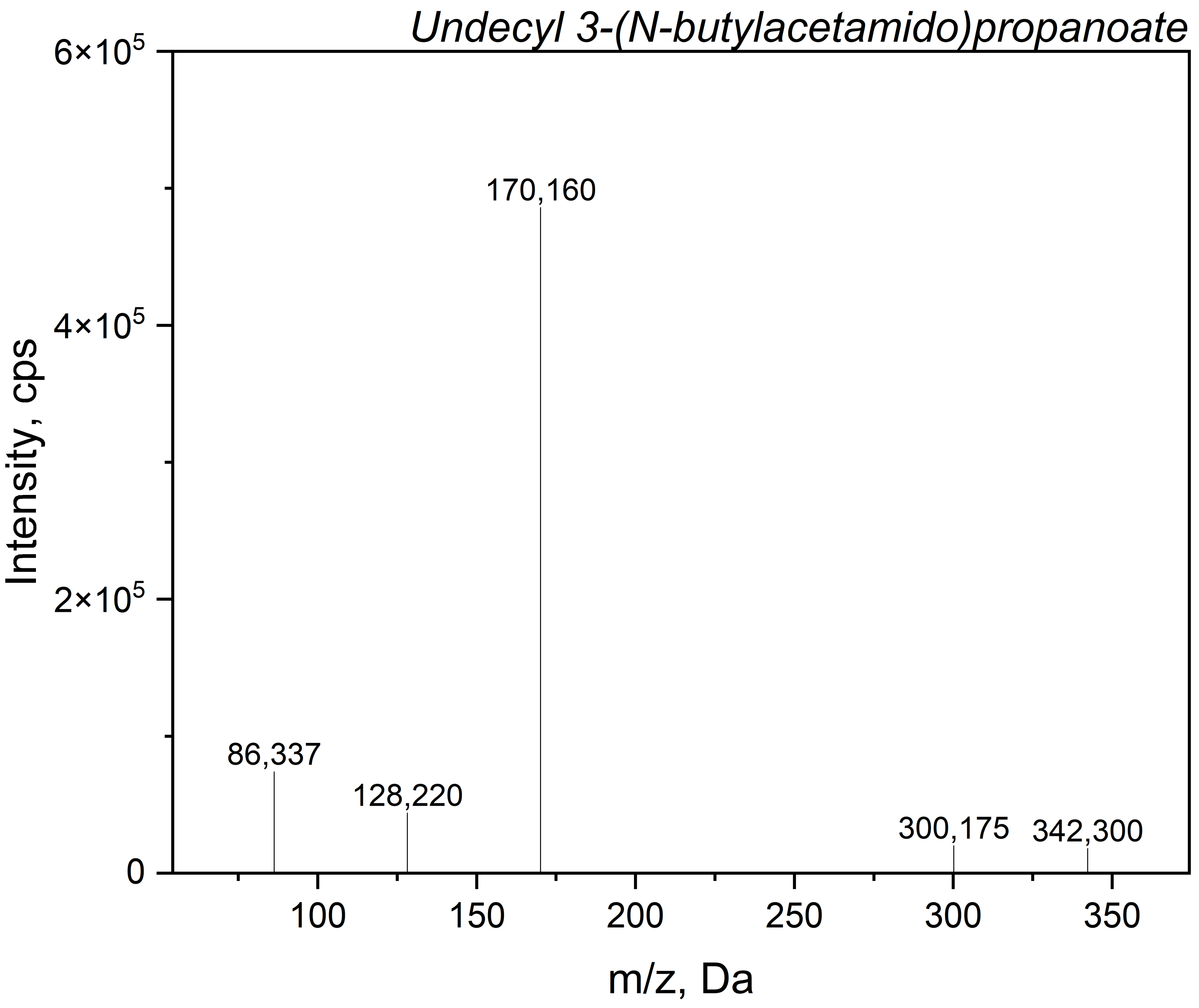

Undecyl 3-(N-butylacetamido)propanoate
6×10^5
4×10^5
2×10^5
0
Intensity, cps
170,160
86,337
128,220
300,175
342,300
100
150
200
250
300
350
m/z, Da

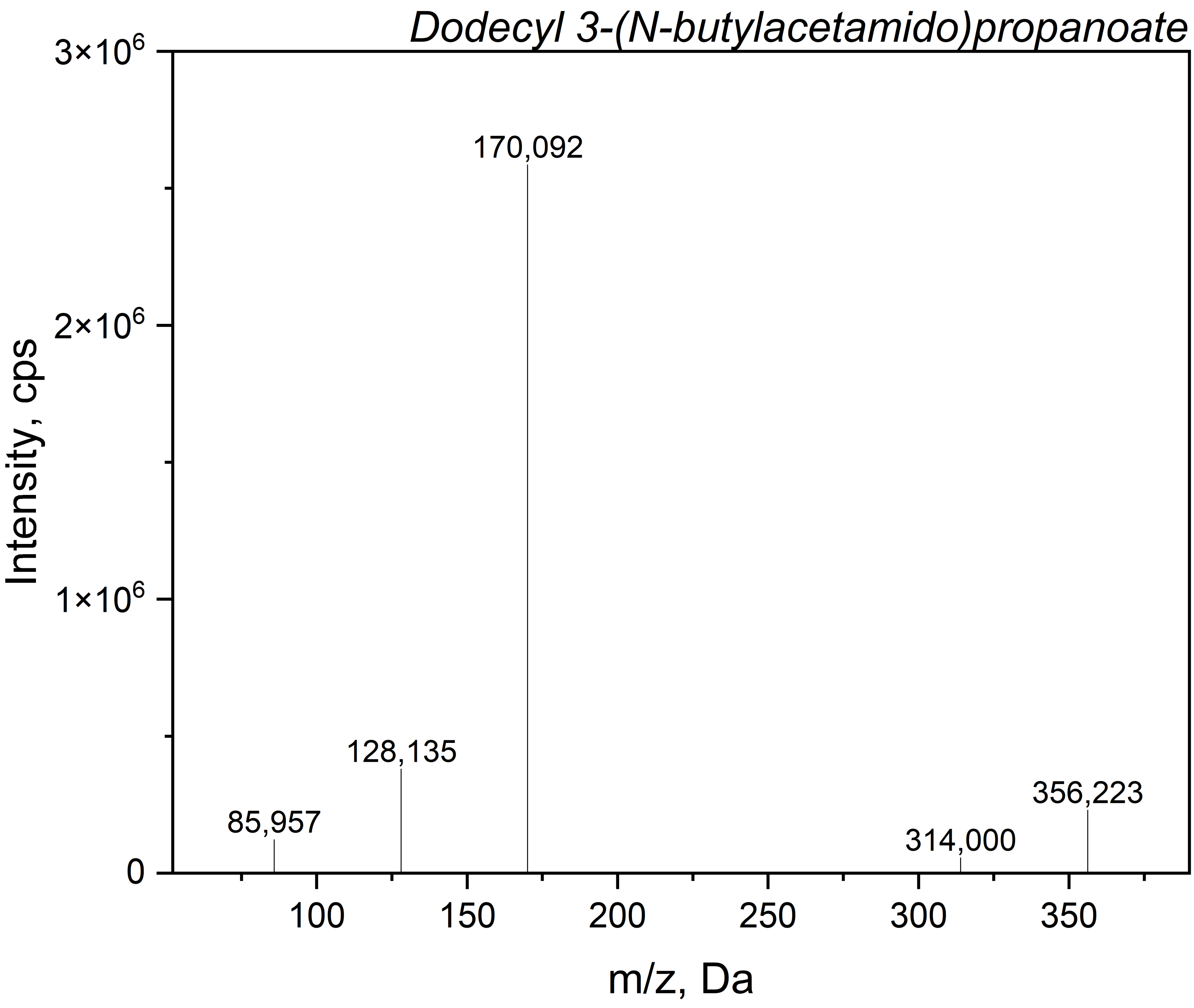

Dodecyl 3-(N-butylacetamido)propanoate
3×10^6
2×10^6
1×10^6
0
Intensity, cps
170,092
128,135
85,957
314,000
356,223
100
150
200
250
300
350
m/z, Da
